# Revolutionizing neural regeneration with smart responsive materials: Current insights and future prospects

**DOI:** 10.1016/j.bioactmat.2025.06.003

**Published:** 2025-06-13

**Authors:** Hongxia Gao, Huoyun Shen, Xunrui Zhang, Yaqiong Liu, Yuqing Shang, Shaolan Sun, Wenchao Guan, Xiaosong Gu, Yumin Yang, Guicai Li

**Affiliations:** a Key Laboratory of Neuroregeneration, Co-innovation Center of Neuroregeneration, Nantong University, Nantong, 226001, China; b Department of Burn and Plastic Surgery, Affiliated Hospital of Nantong University, Medical School of Nantong University, Nantong, Jiangsu, 226000, China; c Medical Science and Technology Innovation Center, Shandong First Medical University & Shandong Academy of Medical Sciences, Shandong, 250117, China

**Keywords:** Smart materials, Stimulus response, Nerve regeneration, Tissue engineering, Nerve grafts

## Abstract

Smart-responsive materials, which possess the capacity to receive signals from the environment and engage in dynamic communication with it, have significantly expanded the frontiers of tissue engineering and regenerative medicine. They hold substantial potential for the evolution of precise and distinctive therapeutic systems within the domain of neural regeneration. In the wake of the continuous advancement of science and technology, researchers have delved deeply into diverse response mechanisms and have successively developed multiple generations of smart responsive materials (SRM) for applications such as drug delivery, graft fabrication, and detection platforms in neural regeneration, yet a lack of a comprehensive and systematic review to provide informative references for researchers in the related fields. Therefore, in this review, we comprehensively summarize the response mechanisms and classifications of smart-responsive materials, with particular emphasis on the research and development progress and application modalities of various SRM in the field of neural regeneration, including peripheral nerve injury, spinal cord injury, and trauma brain injury. Additionally, the limitations of diverse smart-responsive materials in neural regeneration are meticulously dissected, and the efforts for developing new smart-responsive materials for neural regeneration was emphasized. Eventually, the prospects and forthcoming trends of smart-responsive materials in nerve regeneration are prominently spotlighted. It is anticipated that this review will furnish a crucial reference for the utilization of smart-responsive materials across various tissue engineering fields.

## Highlights

• The review provides a comprehensive outlook on SRM applied in neural regeneration.
• The review summarizes the response mechanisms and classifications of SRM.
• The review discusses the research and applications of various SRM in neural regeneration.
• The review analyzes the limitations and future development of SRM in tissue engineering.

## Graphical abstract

**Figure:**
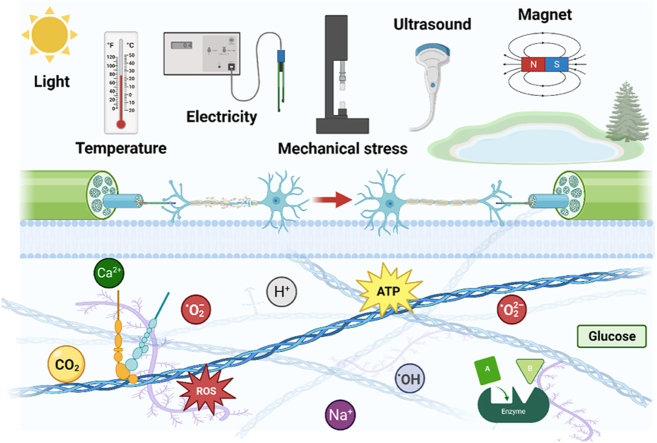

## 1 Introduction

The nervous system is the most complex and highly organized system in an organism. It receives information from the sense organs through the nerves, transmits it through the spinal cord, and processes it in the brain. The nervous system guides an organism's response to the outside world and also controls most of its functions. The nervous system is structurally divided into two parts: the central nervous system (CNS), which includes the brain and spinal cord, and the peripheral nervous system (PNS), which consists of nerves and sensory organs. The CNS serves as a control center, receiving data and feedback from the sensory organs and the nerves throughout the body, processing the information, and then sending back the commands; the neural pathways of the PNS are responsible for the afferent and efferent signals, and work together with the CNS in transmitting and processing sensory information and coordinating body functions [1,2]. The nervous systems of reptiles and amphibians (lizards, salamanders, etc.) have a strong regenerative capacity, and mammals also have a certain degree of regenerative potential after peripheral nerve injury (PNI), whereas CNS injury is difficult to regenerate effectively [3,4].

Nerve regeneration is a long and complex process, and the regenerative microenvironment involves multiple cross-cutting fields such as biochemistry, pathology, biophysics, and bioinformatics, etc. The dynamic process of regeneration involves multiple physiological processes such as inflammatory response, immune response, vascular regeneration, nerve regeneration, and recovery from muscular atrophy [5]. Differences in the type and extent of nerve injury led to very different effects of nerve regeneration. PNI mainly causes damage to the PNS via mechanical or pathological mechanisms, such as physical injury resulting in partial or complete severance, compression, compression, or stretching of the nerve [6]. Among them, the repair effect of stretching or squeezing injury is better than transection, and the recovery effect of distal injury is better than proximal injury [7]. Injuries to the CNS, which include traumatic brain injury (TBI) and spinal cord injury (SCI), can be classified into two categories, primary and secondary injuries, with the former referring to the initial mechanical injury due to local tissue deformation of the injury and traumatic energy transfer, and the latter being the primary injury-inspired including biochemical and cellular alterations, the latter is a chain reaction process including biochemical and cellular alterations, which will further amplify neurological dysfunction in the injured region [8,9]. Although the PNS has a relatively unique regenerative capacity compared to the CNS, regeneration is not equivalent to functional recovery, which makes the technique and strategy of neural repair particularly important.

Based on the perspective of tissue engineering (TE) and regenerative medicine, repair strategies for nerve injury include surgical repair, cell and tissue transplantation, tissue engineering, and gene therapy [10]. Among them, although surgical suture repair is effective, the repair distance is short and cannot be applied to long-distanced nerve defect. Although cell and tissue transplantation can repair damage with long distance, the donor is limited, secondary surgery is prone to infection, and allografts may produce immune rejection [11]. Gene therapy can correct or compensate for diseases caused by defective and abnormal genes, but the ethical and safety issues cannot be denied [12]. TE elevates the traditional treatment model of "trauma repair with trauma" to the level of "manufacturing and regeneration", and steps into a new stage of non-invasive repair, creating a new direction for nerve repair.

TE is the combination of cell biology and material science to construct tissues/organs in vivo and ex vivo to maintain, repair, and regenerate the function of damaged tissues/organs [13]. The key elements of TE include four modules: seed cells, biomaterials, the integration of cells and biomaterials, and the integration of implants and the in vivo microenvironment, among which the development of biomaterials is the foundation, and the three-dimensional scaffold structure prepared by biomaterials can provide a good living environment for cell metabolism [14]. The basic requirement of TE for biomaterials is to possess excellent biocompatibility, and then with the development of society and clinical needs, the performance requirements of biomaterials are more and more clear, such as controllable degradation rate, vascularization ability, anti-inflammatory and antibacterial, providing mechanical properties and hydrophilic properties conducive to the growth of cells, and physiological stimulation required by cells etc [15]. Conventional biomaterials exhibit fixed properties that can only be customized for certain purposes or applications and are not adapted to changes brought about by environmental or biological stimuli [16]. Compared to traditional materials, Smart responsive materials (SRM) with tunable properties can provide individually designed biomedical products that are better adapted to and facilitate peripheral nerve repair in a more advanced way, thus developing new repair strategies for neural regeneration [17].

Stimulus response phenomena are prevalent in nature. Legumes such as mimosa can respond to mechanical stimuli by folding their leaves when touched, and the greater the force of the touch, the faster they close [18]. Cephalopods such as the squid have millions of pigment cells gathered in their bodies, which can respond within a second to adjust the size of the pigment sacs in their bodies to change their color in order to adapt to their environment and escape from enemies [19]. Inspired by nature, researchers have developed a series of responsive materials. There have been several reports on SRM in the field of neural regeneration [[20], [21], [22]], yet there is a lack of a comprehensive and systematic review to provide informative references for researchers in related fields. This review provides a comprehensive overview of SRM from a broad perspective, focusing on the functional types of SRM, and their applications in the field of neural regeneration, including CNS and PNS. In addition, this review emphasizes the efforts that can be made in developing new SRM to promote their applicability and utility in the field of neural regeneration and other tissue engineering fields. Finally, an in-depth analysis of the challenges that need to be addressed in the practical application of SRM is presented.

## 2 General concept and summary of SRM

**Fig. 1:**
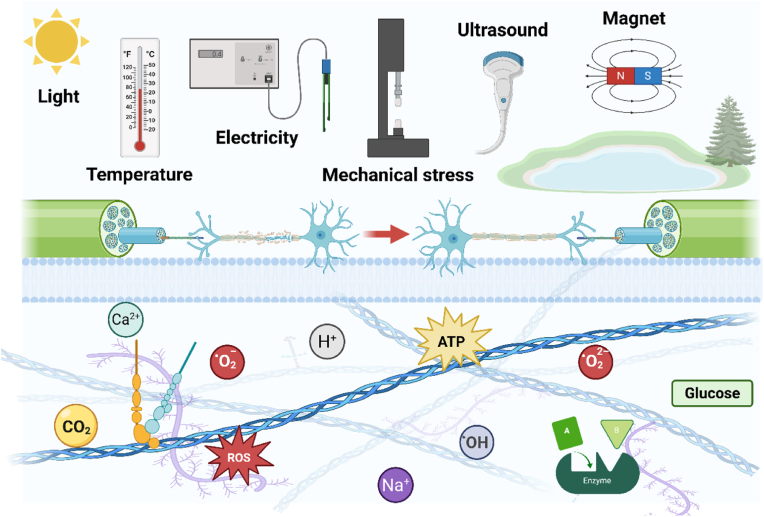
Various external and internal stimulating factors for tissue regeneration via SRM. Created by biorender.com.

Firstly, "Smart" refers to the ability of a material to sense changes in its environment and use physical or chemical cues to guide tissue regeneration. Secondly, "Responsive" refers to the ability of an implant to "communicate" back and forth with surrounding tissues/cells, i.e., to send and receive signals. On the one hand, the material stimulates the living tissue to initiate the regeneration process. On the other hand, the materials can adapt their physical structure to respond to the environment as needed [23]. That is to say, SRM is a new type of material that can sense endogenous or/and exogenous stimuli and can perform corresponding functions itself, with the characteristics of structural functionalization and functional diversification, which is also called stimulus response material [24]. SRM not only mimics the tissues/cells structurally, but also communicates with the living organism's for dynamic communication to achieve intelligent applications [25,26]. The response signals of SRM can originate from outside the organism (temperature, magnetism, electricity, force, light, etc.) or from inside (pH, ROS, ions, enzymes, etc.) as shown in Fig. 1, and stimulus-triggered responses can be based on physical changes, chemical changes, or a combination of the two, and thus choosing the right stimulus response is crucial to achieve the desired application [27]. Therefore, a deeper understanding of the types of responses and the applications of SRM can contribute to the innovation of key fundamental research, which in turn leads to the development of more relevant applications in various tissue engineering fields.

## 3 Various external stimulants

### 3.1 Temperature

**Fig. 2:**
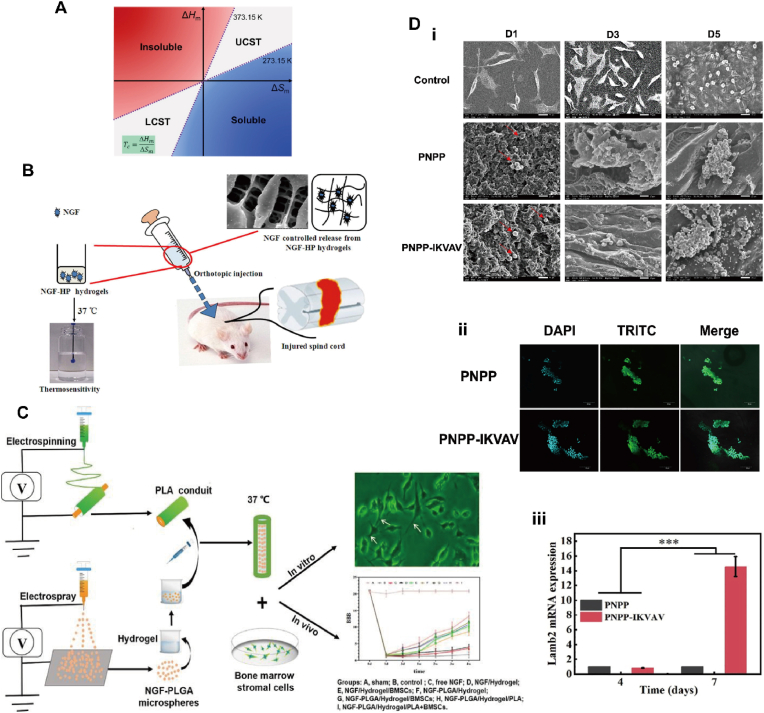
**(A)** A thermodynamic map showing the solubility and solution properties of polymers in water [28]. Copyright 2019, Elsevier. **(B)** A novel heparin-poloxamer (HP) thermo-sensitive hydrogel to enhance the spinal cord regeneration of NGF [64]. Copyright 2016, Elsevier. **(C)** (i)The preparation schematic diagram of the composite nanotube. (ii)BBB score [49]. Copyright 2022, Elsevier. **(D)** The adhesive performance of the NSCs in hydrogel scaffolds. (i) The SEM images of PNPP and PNPP-IKVAV hydrogels in 1 day, 3 days and 5 days. Scale bars = 20 μm. (ii) The nuclear membrane staining of PNPP and PNPP-IKVAV hydrogels at 7 days after seeding. Scale bars = 100 μm. (iii) Relative gene expression level of Lamb2 in the PNPP group (control) and the PNPP-IKVAV group (sample) at 4 and 7 days [53]. Copyright 2020, Elsevier.

Temperature is an easy-to-apply in vitro stimulus and one of the most widely explored elements in the design of stimulus response platforms. Typically, there are two types of temperature-responsive polymers, including the low critical solvation temperature (LCST) type and the high critical solvation temperature (UCST) type [28], as shown in Fig. 2A. Due to the hydrophilic/hydrophobic equilibrium between the polymer chains, LCST polymers undergo phase transitions from hydration to dehydration as the temperature increases. In contrast, UCST polymers undergo the opposite phase transition during heating, where strong interactions between the polymers form in solutions below the critical temperature, and an increase in temperature disrupts these interactions, leading to polymer dissolution [29]. Molecular weight, polymer concentration, termination groups and the presence of co-solubilizers all affect the critical temperature of the material [30]. In addition, biological macromolecules such as proteins and DNA provide a variety of design ideas for temperature responsive materials [31].

LCST is the dissolution temperature of polymer in solution, when the temperature exceeds LCST, the polymer will precipitate from the solution or phase separation phenomenon occurs. LCST type polymers are usually water-soluble polymers, at lower than LCST, hydrophilic groups and water molecules form a stable hydrogen bond. The macromolecular chains in water are sufficiently stretched to present a single-phase soluble state, whereas at higher than LCST, the hydrogen-bonding connection between water molecules and hydrophilic groups is broken, and hydrophobicity becomes the main role, which manifests itself as an insoluble form at the macroscopic level [32]. This phase separation phenomenon can be reversible, i.e., the polymer can be re-solubilized on lowering the temperature, or it can be irreversible [33,34]. Common LCST-type polymers mainly includes poly(N-isopropylacrylamide) (PNIPAM) [35], polyalkyl oxazolines [36], elastin-like polypeptides (ELPs) [37], and poly amino acid hydrogels [38,39].

UCST polymers are polymer systems that exhibit reversible phase separation behavior in a certain temperature range, where solubility decreases with increasing temperature and phase separation occurs when the temperature exceeds a critical value. From a thermodynamic analysis perspective, strong interactions between the solvent and the polymer chains (e.g., hydrogen bonding, dipole-dipole interactions) dominate the solution process in the low-temperature region, whereas elevated temperatures disrupt this equilibrium and initiate phase separation. Compared to LCST-type polymers, UCST-type polymers are relatively less studied and applied, such as poly-N-acryloylglycinamide [40,41], urea-modified polymers [42], and hydrophobically modified poly(propionamide) [43], in addition, carboxyl group-containing polypeptide-like polypeptides exhibit a designable UCST behavior [44].

In neuroregeneration applications, temperature-responsive materials copolymerized with degradable substance can be used to prepare carriers such as microspheres and nanoparticles for drug delivery and slow release, and can also be used to control the rate of drug release by temperature for drug delivery at specific sites [45,46]. Zhao [47] et al. prepared an NGF-HP hydrogel that could be loaded with neurotrophic factor NGF, which had a gel temperature suitable for the human body (≥37 °C), and could release the loaded factor by in situ controlled release according to the temperature change, which not only had a good affinity for NGF, but also prevented the protease degradation and promoted the regeneration of the rats spinal cord, as shown in Fig. 2B. Alizadeh and Moradi [48] used chitosan (CS), β-glycerophosphoric acid disodium salt pentahydrate (β-GP) as the gelatinizing agent, and hydroxyethylcellulose (HEC) as the cross-linking agent, to prepare a CS/β-GP/HEC hydrogel by mixing method. The hydrogel was wrapped with human adipose-derived mesenchymal stem cells (hADSC) and delivered NGF to the damaged area, inducing specific neuronal growth and repairing the damaged spinal cord, providing a protective and effective microenvironment for nerve regeneration. Song [49] et al. prepared temperature-sensitive hydrogels with sodium alginate (SA), poloxamer (P407, P188), and CaCl_2_ co-mixture, and encapsulated NGF-loaded poly(lactic-co-glycolic acid) (PLGA) microspheres and bone marrow mesenchymal stem cells (BMSCs) into poly(lactic acid) (PLA) fibrous nanotubes (Fig. 2C). PLGA microspheres could protect and provide long-term controlled release of NGF to ensure sufficient release time and drug concentration to promote neuronal cell growth and axonal regeneration. The temperature-sensitive hydrogel not only provided an adjustable physiological analog microenvironment, but also a suitable support matrix for cells and drugs. The PLA nanotubes ensured the sealing of released NGF in the nanotubes to enhance its drug bioavailability for supporting and guiding nerve growth.

In addition, temperature-responsive materials after modification with good biocompatibility and degradability can be applied for tissue engineering, such as various scaffolds, nerve conduits, etc., which can play their roles in specific environments to promote tissue/cell regeneration [50,51]. Yin [52] et al. prepared a chitosan (CS) temperature-sensitive hydrogel nerve conduit modified with decellularized neural matrix (DNM) and added polypyrrole (PPy) to improve the mechanical properties of the nerve conduit, and the additions of DNM and PPy resulted in a composite nerve conduit with suitable biodegradation properties to support the adhesion and growth of nerve cells. A temperature-sensitive hydrogel grafted with the bioactive peptide IKVAV (Ile-Lys-Val-Ala-Val) was developed by Dai's group [53]. The hydrogel was prepared by copolymerizing N-prop-2-ylprop-2-enamide (NIPAM) and AC-PEG-IKVAV copolymer by reversible addition-fracture chain transfer (RAFT) polymerization using poly (ethylene glycol) (PEGDA) and N, N′-methylene bis-acrylamide (BISAM) as cross-linking agents. The hydrogel scaffolds exhibited fast (de)swelling properties, good biocompatibility, and regular three-dimensional porous structure, which could guide cell fate and mediate neuronal differentiation as displayed in Fig. 2D. Temperature-responsive materials are also subjected to reversible changes in phase transition after modification. Garbay [54] et al. grafted laminin IKVAV sequence and interfering VKAIV sequence on ELP, respectively. Interestingly, the phase transition of ELP-VKAIV was fully reversible, but ELP-IKVAV underwent irreversible aggregation. Rat primary neurons were cultured on the hydrogel formed by ELP-IKVAV and functionalized polyethylene glycol (PEG), and showed a significantly larger neurites of 20 % sensory neurons.

The neuroregenerative effects demonstrated in the above studies require that the tissues and cells of the organism interact with temperature-responsive materials to facilitate dynamic communication, thus how do the tissues and cells respond to temperature when the materials respond to temperature changes? Transient receptor potential TRP ion channels are a class of channel proteins that are widely distributed in the peripheral and central nervous system and are responsible for a variety of responses to heat, cold, pain, and stress [55]. There is evidence that TRP channels regulate intracellular Ca^2+^ concentration and influence phagocytosis [56]. Among these, the TRPV1 receptor is a molecular integrator of thermal pain stimuli and chemical stimuli that is mainly expressed in non-myelinated injury-sensing neurons with specific thermal sensitivity to inflammation and changes in body temperature (>43 °C) [57]. Upon activation of TRPV1, the ion channels are opened, which increases permeability to cations, mediates the inward flow of Ca^2+^ into the cell to effectively depolarize the mitochondrial Ca^2+^ and caspase activation, and promotes the release of reactive oxygen species (ROS), leading to the expression of pro-apoptotic activity and neurotoxicity [58,59]. Subsequent studies have gradually revealed that TRPV1, TRPA1, TRPM2, and TRPM3 ion channels act together as thermoreceptors, but heat sensation is only effectively transmitted when cold-sensing nerve fibers containing TRPM8 are simultaneously inhibited by heat-sensing temperatures [60].

Although temperature-responsive materials can achieve morphological and structural changes through temperature changes and thus modulate nerve cell growth and regeneration to some extent, their applications still face some challenges and limitations. Firstly, temperature-responsive materials usually have a specific response temperature range. In the field of neural regeneration, the temperature of the cell growth environment needs to be precisely controlled to promote the regeneration and reconstruction of nerve cells. It has been shown that changes in extracellular temperature can affect neuronal growth to varying degrees [61,62], e.g., Shunsuke Chuma et al. investigated the cellular response to localized (<5 μm) heating with infrared laser irradiation and found that local temperature elevation of a certain duration (>20 min) and magnitude (3 °C), especially in the nucleus, could promote or induce neuronal differentiation during neuronal growth [63]. However, the specific response temperature range of temperature-responsive materials may not be able to satisfy this need, and thus more precise methods of temperature regulation need to be developed. Secondly, the materials need to interact with neural tissues/cells, making their biocompatibility a critical factor. When employing temperature-sensitive materials, comprehensive biocompatibility assessments and safety studies are imperative to ensure that their impact on tissues/cells remains within acceptable limits. Given that thermo-responsive materials may exert mechanical stimulation on cells during temperature elevation or phase transition, potentially activating inflammatory pathways, strategies such as incorporating anti-inflammatory drugs/active molecules and implementing biological modifications can be adopted to mitigate associated inflammatory risks. Finally, temperature-responsive materials may face stability issues in long-term in vivo applications. Neural regeneration requires materials to maintain their temperature-responsive properties over time to continuously regulate nerve cell growth and regeneration. Future research needs to address these issues and further improve the performance of temperature-responsive materials to enable more precise, reliable, and effective therapeutic approaches to neural regeneration.

### 3.2 Magnetism

**Fig. 3:**
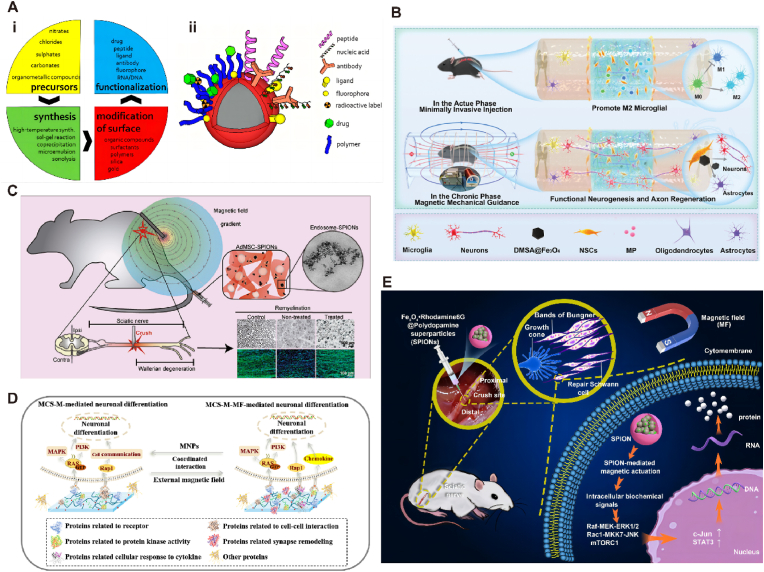
**(A)** The scheme of magnetic particles design workflow (i) and possible modification and functionalization of magnetic particles (ii) [68]. Copyright 2017, MDPI. **(B)** The diagram of the design and fabrication of delivery system loading DMSA@Fe_3_O_4_ and MP for the repair of spinal cord injury. This delivery system alleviated the inflammatory microenvironment in the acute phase and promoted functional neurogenesis and axon regeneration with the static magnetic guidance in the chronic phase [71]. Copyright 2024, Wiley. **(C)** adipose-derived mesenchymal stem cells (AdMSC) were loaded with citric acid coated superparamagnetic iron oxide nanoparticles (SPIONs), systemically transplanted and magnetically recruited to the injured sciatic nerve [72]. Copyright 2021, Elesvier. **(D)** Magnetic chitosan hydrogel and externally-applied magnetic field synergistically promote neuronal differentiation by modulating the formation of protein corona to activate intracellular signaling cascades [75]. Copyright 2023, Elesvier. **(E)** Magnetically driven, SPION-mediated mechanical forces can be sensed by Schwann cells and transduced into intracellular biochemical signals that promote nerve regeneration by inducing and maintaining the repair phenotypes of Schwann cells [74]. Copyright 2022, Springer Nature.

The magnetic properties of matter are not only universal, but also diverse. Magnetically responsive materials are a class of materials that can respond to changes of magnetic fields. They can be stimulated by an external magnetic field to change in shape, size, or magnetic properties. The response mechanism of magnetically responsive materials is usually based on the magnetic properties of the material, such as magnetic moment, magnetic field or magnetization [65]. From TE and regenerative medicine perspective, magnetic nanoparticles (MNPs) have modifiable magnetic, optical, electrical, and thermal properties of magnetically responsive materials due to their nanoscale size, which results in physical and chemical properties different from those of macroscopic-sized materials, and MNPs have a high specific surface area that allows for better interactions with other materials or molecules [66], e.g. Fig. 3A, thus MNP is a potential biomaterial candidate in the field of tissue regeneration. Common magnetoresponsive materials in neural regeneration include iron oxide magnetite (Fe_3_O_4_) and magnetic hematite (γ-Fe_2_O_3_), which exhibit chemical stability, low toxicity, and high magnetization under physiological conditions [67]. Some other magnetically responsive materials, such as chromium, nickel, and manganese, are less biocompatible and need to be applied with a stability coating to avoid leakage of toxic metals that can affect neural tissue regeneration [68].

In the field of nerve repair and regeneration, MNPs can be combined with various bioactive substances, such as nerve cells or growth factors, etc, to form composite materials or serve as carriers for the delivery of growth factors, drugs, or other substances beneficial for nerve regeneration [69]. Chen [70] et al. prepared a biocompatible multilayer composite conduit with suitable stiffness by using poly(epsilon-caprolactone) (PCL) as the outer layer, Fe_3_O_4_-MNPs/PCL as the middle layer, and melatonin (MLT)/PCL as the inner layer, using electrostatic spinning technology. In vivo animal experiments showed that the myelin regeneration, axonal regeneration, and functional restoration including morphological and electrophysiological evaluation of this conduit were very close to that of auto-transplantation, showing greater neuronal regeneration potential. Zhang [71] et al. developed a delivery system containing MNPs and Methylprednisolone (MP). In the acute phase, the delivery system could release MP to promote microglia M2 polarization, inhibit M1 polarization, and reduce neuronal apoptosis, and in the chronic phase, it promoted the differentiation of NSCs into functional neurons under the magneto-mechanical stimulation generated by MNPs, as exhibited in Fig. 3B. MNP could be manipulated by an external magnetic field to achieve the targeting and release of cells/drugs in order to improve therapeutic efficacy and nerve regeneration efficiency. Soto [72] et al. significantly improved nerve conduction by recruiting magnetically targeting adipose MSCs loaded with superparamagnetic iron oxide nanoparticles (SPION) to the sciatic nerve (Fig. 3C). This non-invasive and non-traumatic approach manipulated by a magnetic field improved cellular recruitment in damaged nerves and facilitated neuromyelin regeneration and functional recovery.

MNP can promote neuronal cell repair by cooperating with magnetic fields to produce mechanical stimulation or stretching in neural tissues [73,74]. He et al., [75] prepared a magnetic chitosan hydrogel by introducing MNP into CS hydrogels. In vitro and in vivo studies demonstrated that the MNP-infiltrated chitosan scaffolds exerted significant modulatory effects on neuronal differentiation and promoted neuronal regeneration and enhanced motor recovery. It was also found that magnetic chitosan hydrogels altered the composition of the protein corona and contributed to an increase in the concentration of proteins associated with "cell-to-cell interactions", "receptors" and "protein kinase activity", leading to neuronal differentiation. Magnetic chitosan hydrogel responded to external magnetic fields and produced a synergistic effect that further enhanced the differentiation of neural stem cells (NSCs) into neurons and accelerated neural regeneration, as shown in Fig. 3D. It has been shown [76] that Schwann cells (SCs) can sense and signalize external mechanical stimuli. Wang and Liu [74] et al. designed and prepared a novel fluorescent-magnetic bifunctional SPIONs and established a magnetic field stimulation system to apply the SPIONs to the sciatic nerve for the magnetoactivation of SCs (Fig. 3E).The results showed that the mechanical forces mediated by magnetically actuated SPIONs could be sensed by SCs and transduced into intracellular biochemical signals to promote nerve regeneration by inducing and maintaining the repair phenotype of SCs. In addition, the use of combined magnetic and thermal approaches for tissue engineering has become commonplace and therapeutically effective. Huang [77] et al. targeted manganese ferrite (MnFe_2_O_4_) nanoparticles into cells which expressed the temperature-sensitive ion channel TRPV1 and heated them using a radiofrequency magnetic field. The localized temperature increase could open the TRPV1 channel and cause Ca^2+^ inward flow as proved by using fluorophores as molecular thermometers. The generation of this localized magneto-thermal effect enabled remote neural excitation and promoted the process of neural regeneration.

The organism itself is a medium characterized by electromagnetic distribution, containing magnetic particles that can be connected to pressure-sensitive ion channels in the cell membrane through scaffolds and deflected or moved in response to changes in external magnetic field conditions, and such changes affect the function of membrane proteins, which can lead to changes in the permeability of the cell membrane and cell membrane-specific ions, ultimately affecting the regulation of cellular function [78,79]. In 2015, studies firstly reported the magnetoreceptor gene for the perception of magnetic fields in animals, which encoded a magnetoreceptor protein (MagR) with endowed magnetism that could form a complex with Cry proteins and thus recognized and responded to external magnetic fields. Besides, the specificity of Cry4 proteins for the induction of magnetic fields was also demonstrated in 2021 [80]. Among them, the iron-sulfur cluster protein IscA1 has been shown to be widely present in living organisms and is mainly involved in cellular energy metabolism, electron transfer, substrate binding and activation, iron/sulfur storage, enzymatic reactions, and gene expression regulation [81]. IscA1 is able to regulate the expression of relevant magnetic genes through the activation of MagR by external magnetic field stimulation, affecting neural activity and behavioral localization [82].

Despite the notable enhancing effect of magnetism, certain practical challenges remain. Firstly, Magnetic-responsive materials can modulate the behavior and growth of neural cells by means of an external magnetic field, but due to the limited depth of penetration of the magnetic field, conventional magnetic-responsive materials may not be able to provide sufficient magnetic field strength and range of influence for deeper tissue structures, such as brain tissue. Therefore, when using magnetically responsive materials, the penetration depth of the magnetic field and its influence range on the target tissue need to be considered. Secondly, magnetically responsive materials should have sufficient long-term stability to maintain their magnetic field response properties in long-term applications. For nerve regeneration and repair, magnetically responsive materials with degradability may be more advantageous to facilitate tissue regeneration and bioabsorption. Thirdly, cells and proteins in living organisms have very weakly magnetic properties, thus given stimulating a biological response using magnetic fields, a high magnetic field strengths in excess of 1 T generated by pulsed magnetic materials or magnetic materials converting weak magnetic stimuli (typically less than 100 mT) into a form of energy that can be used to stimulate nearby cells should be used [83]. Lastly, material-based magnetic stimulation techniques that enable millisecond timing would enable many neurotherapeutic and applications that require neuromodulation precisely synchronized with sensory stimulation and behavior. However, the existing magnetic materials struggle to achieve millisecond-level timing accuracy, and therefore, there is a need to develop novel magnetically responsive materials as well as to explore novel stimulation technique.

### 3.3 Electricity

Since ancient times, "electricity" has been utilized by various living organisms, such as neuronal signaling and muscle contraction. Electro-responsive materials are a class of stimulus response system that is capable of changing their mechanical or physical-chemical properties in response to an external electric field. Electro-responsive materials, which are widely used in biomedical engineering and tissue engineering, are mainly referring to electroactive polymers (EAPs) [84,85].

**Fig. 4:**
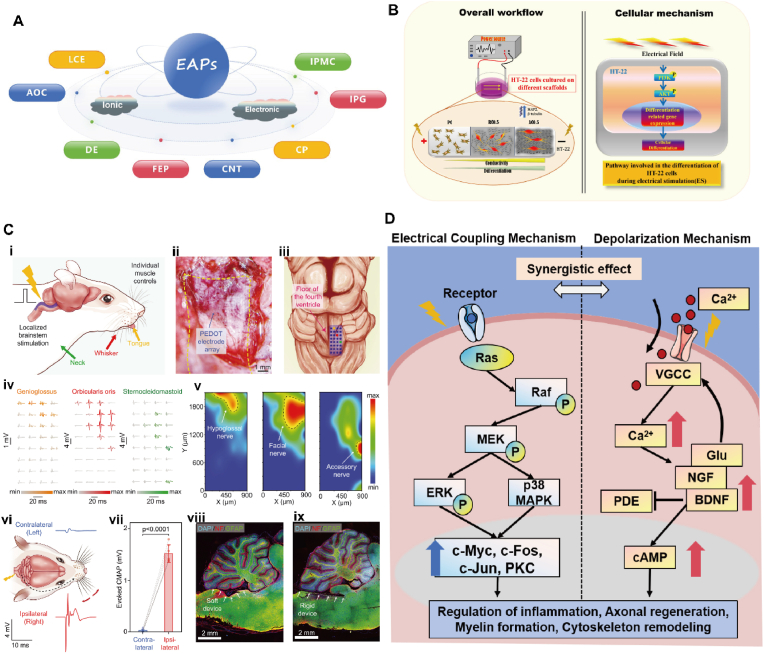
**(A)** Classification of EAPs. **(B)** Schematic representation of the effect of electrical stimulation on HT-22 cells cultured on different types of scaffolds and scaffold combined with electrical stimulation modulates intracellular mechanism [108].Copyright 2022, Elsevier. **(C)** Stretchable high-density array allows localized neuromodulation for precise control of individual muscle activities. (i) Schematic diagram illustrating the application of the stretchable electrode array for precise neuromodulation through localized brainstem stimulation. (ii) Microscopic image of a stretchable electrode array conforming to the curved floor of the fourth ventricle with 50-μm electrode width. (iii) Schematic illustration of a multielectrode array placed on the right side of the brainstem. (iv) Evoked muscle activities recorded at the tongue (left), whisker (middle), and neck (right) after electrical stimulation at the brainstem. (v) Activation maps based on the muscle activities depicting the spatial distribution of different nuclei (marked by dashed lines) in the brainstem with downstream connections to the hypoglossal nerve (left), facial nerve (middle), and accessory nerve (right). (vi) Schematic diagram and representative data traces showing the side specificity of the brainstem stimulation. (vii) Statistical analyses showing the preferred activation of ipsilateral targets. CMAP, compound muscle action potential. (viii,ix) Immunohistological staining of a brain slice after the insertion of the soft and stretchable electrode array (viii) and a rigid device (ix) along the floor of the fourth ventricle between the brainstem and the cerebellum. DAPI, 4′,6-diamidino-2-phenylindole; NF, neurofilament; GFAP, glial fibrillary acidic protein [122]. Copyright 2022, AAAS. (**D)** Two possible mechanisms between the interaction of ES and biological systems.

With the development of functional materials, EAPs have been found to possess a good potential to respond to applied electric fields. The mechanical deformation produced by EAPs in response to electrical stimulation (ES) can be reversible by changing the molecular arrangement within the polymer [86]. Based on the mechanism of action, EAPs are mainly categorized into two main groups in Fig. 4A, electronic-type EAPs and ionic-type EAPs [87]. Electronic-type EAPs include all-organic composites (AOCs) [88], dielectric elastomers (DEs) [89,90], ferroelectric polymers (FEPs) [91], and liquid crystal elastomers (LCEs) [92]. Ionic EAPs include carbon nanotubes (CNTs) [93], conductive polymers (CPs) [94], electrorheological fluids (ERFs) [95], ionic polymer gels (IPGs) [96,97], and ionic polymer-based metal composites (IPMCs) [98].

Electron-based EAPs are driven by Coulombic interactions (electrostatic forces) generated by electric fields or charges on a localized scale. Strain manifests itself as a molecular, microscopic or macroscopic phenomenon in response to an external applied electric field. The Coulomb force induces electrostrictive effects as well as electrostatic, piezoelectric and ferroelectric effects in the presence of an electric field, and such EAP materials can be induced to shift in the presence of a DC electric field. However, at a certain electrostriction effect, electronic-type EAPs require a high excitation electric field (>100 V/μm), which is close to the breakdown electric field of the material [99]. Ionic-type EAPs are composed of two electrodes and one electrolyte, and ionic migration or dispersion effects can cause excitation of such materials at lower voltages (1–2 V) with induced bending displacement [100]. The disadvantages of these EAP materials are including follows: on the one hand, they need to maintain a certain degree of wetting. On the other hand, it is difficult for them to maintain a stable induced displacement under DC field excitation (except for conductive polymers) [99]. But the complexity between various types of EAPs can be used to complement their properties, and several EAPs commonly used in the field of nerve regeneration are described below.

#### 3.3.1 CNT

CNTs are nano-scale tubular structural objects composed of carbon atoms with unique physical and chemical properties. Structurally, CNTs can be single-walled carbon nanotubes (SWCNT) or multi-walled carbon nanotubes (MWCNT). Dimensionally, CNTs typically have a diameter in the nanometer range and can vary in length from nanometers to micrometers, with very high aspect ratios. Mechanically, CNTs possess very high mechanical strength and elasticity modulus, as well as excellent tensile strength and elasticity, and are considered to be one of the toughest materials that currently known [101,102]. In addition, CNTs have excellent electrical and thermal conductivity properties and can exhibit metallic or semiconducting properties, conduct heat efficiently, and display excellent electron transport properties [103].

CNTs, with physicochemical properties adjusted by surface modification, have different effects on cells. Machado [104] et al. produced two different densities of vertically-aligned CNT (VACNT) scaffolds on Ti using Ni or Fe as catalysts. Plasma was used to improve the hydrophilicity of the scaffolds, and the density and hydrophilicity of the CNT arrangement modulated the cellular adhesion behavior. Kunisaki [105] et al. prepared CNT fiber (cYarn®; LINTEC OF AMERICA INC., Richardson TX, USA) scaffolds by oxidizing the carbon nanotubes for high hydrophilicity, which improved the biocompatibility of the scaffolds and facilitated the repair of 15-mm peripheral nerve defects while controlling the foreign body reaction and reducing inflammation. CNT, as a constituent material of nerve regeneration, is highly mechanically strong and flexible, and can provide support and guidance to the growth and regeneration of nerve cells. The nanoscale structure of CNT can also mimic the natural structure of neural tissue, providing a better environment for cell adhesion and growth. Nazeri [106] et al. used vertically aligned laminin-coated poly (lactic-co-glycolic acid)/CNT nanofibrous conduits to evaluate the effects of biochemical cues and ES on sciatic nerve regeneration. The results showed that the tubes were able to promote cell attachment and proliferation and improve sciatic nerve regeneration. Nascimento [107] et al. fabricated a vertically aligned carbon nanotube (VA-CN) with a three-dimensional structure, which resulted in an enhanced cellular adhesion and communication, due to the interplay between its surface roughness and 3D morphology mimicking that of the natural extracellular matrix, facilitating the regeneration of the neuronal protrusions and the ability of network-formation.

CNT has excellent conductivity properties and can be used to assemble neural ES devices. By integrating CNT into the electrode material, precise stimulation of nerve tissues can be achieved to promote regeneration and connectivity of nerve cells. This is important for the treatment of nerve injury and the recovery of nerve function. Ghosh [108] et al. prepared conductive scaffolds by adding carboxyl-functionalized MWCNTs to PCL-collagen polymer matrices, and the carbon nanofiller reinforcement led to an increased tendency of the scaffolds to biodegrade, as well as improved the mechanical and electrical properties of the scaffolds as shown in Fig. 4B. In vitro evidence showed that the growth and differentiation of HT-22 cells on the scaffolds were significantly improved by external ES, which was favorable for nerve regeneration. Liu [109] et al. prepared three-dimensional conductive GelMA-MWCNTs/Co hydrogel scaffolds by doping MWCNTs/Co composites into gelatin methacryloyl (GelMA) hydrogel matrix. The scaffold exhibited high electrical conductivity and good biocompatibility, and the stem cells from apical papilla (SCAP) exhibited significant neuronal cell-like changes and significant higher neuron-specific marker expression levels after 7 days of ES.

In addition, the high specific surface area and drug-carrying capacity of CNTs make them effective drug delivery platforms, which can improve drug stability and efficacy [110]. Mishra [111] et al. prepared composite neural scaffolds with collagen + PCL + MWCNT, which were loaded with rat bone marrow-derived mesenchymal stem cells (rBMSC) and insulin-like growth factor I (IGF-I), respectively. The carbon nanotube scaffolds provided electrical conductivity for proper neuronal regeneration, the loaded rBMSC induced axonal regeneration and cellular transformation, and IGF-I induced stem cell differentiation, myelin synthesis, angiogenesis and muscle differentiation.

#### 3.3.2 CP

CP refers to materials based on polymers that are made electrically conductive by doping them with conductive materials (e.g., carbon nanomaterials, metal nanoparticles, etc.) or by introducing conductive groups (e.g., pyrrolidone, imidazole, etc.). CPs have a conjugated Π-bonding system in their backbone, which creates a large number of loosely bonded chains of electrons. Ionic functional groups such as carboxyl, sulfonic, phosphoric and amine groups, can be introduced into the polymer to produce ionically conducting (IC) polymers [112]. In vivo applications require CPs with good electrical conductivity and biocompatibility that can mimic the electrophysiological properties of neural tissues and promote nerve regeneration and repair [113]. Common CPs include polyaniline (PANI) [114], polythiophene (PTh) [115], PPy [116] and their derivatives.

CP has emerged as a promising class of materials in the treatment of neurological disorders and the promotion of nerve regeneration due to its unique electrical conductivity and biocompatibility [117]. CP can be used as a scaffold material for nerve repair and regeneration, by providing mechanical support and electrical conductivity, and facilitating neuronal cell adhesion, migration, and growth. Yan [118] et al. copolymerized poly(3,4-ethylenedioxythiophene)/polystyrene sulfonic acid/polyvinyl alcohol (PEDOT/PSS/PVA) copolymerization to form an interpenetrating conductive polymer network (ICPN), and the ICPN scaffolds displayed strong interfacial adhesion, excellent mechanical properties, and stable electrochemical properties, and were highly biocompatible, which are well suited to improve the tissue response and the quality of signal acquisition at the electrode-neural tissue interface. Escobar [119] synthesized novel PEDOT nanoparticles (PEDOT NPs) by the microemulsion method, which were then combined with silk fibroin (SF) to prepare a nerve conduit. The conduit had excellent mechanical properties and good flexibility and electrical conductivity, and could promote the proliferation of SCs, inhibit the infiltration of fibroblasts, and avoid the formation of intraluminal scar tissue.

The conductivity of CP enables good electrical coupling with neural tissue, resulting in high quality signal acquisition and stimulation for building neural interfaces and electrophysiological interfaces. CP can also be used to construct biosensors and diagnostic devices for monitoring and detecting neural-related physiological signals. Coating conventional metal electrodes with conductive polymers has achieved essential properties required for bioelectronics. Zhang [120] et al. seamlessly coupled conventional electrodes with conductive hydrogel coatings, which showed tissue-like modulus, highly desirable electrochemical properties, robust interfaces, and long-term reliability. Stable electrophysiological recordings were achieved by in vivo implantation in a freely moving mouse model, and the conductive hydrogel-electrode interface remained robust under long-term low-pressure ES. In addition, a sensor was prepared by doping CNTs in PEDOT: PSS, which was mechanically matched to neural tissues and highly biocompatible, and enabled neurosensing in vivo [121]. A flexible stretch electrode was co-developed by the scientists from China and the United States [122], where polyrotaxanes (PRs) consisting of polyethylene glycol (PEG) backbone and cyclodextrins were systematically introduced into flexible electrodes made of conductive polymers (PEDOT: PSS). As shown in Fig. 4C, the electrode had high conductivity and crack strain in physiological environments, and also allowed for cell-scale photomorphology, which were beneficial for the recording of neural interface electrical signals and the implementation of precise stimulation in brain-computer interfaces.

In addition, CPs can be used to construct in vitro models of neural tissues or to simulate the microenvironment of neural tissues for the study of neurodevelopment, disease mechanisms and drug screening. An organic electrochemical transistor (OECT) based on a hybrid ion-electron conducting polymer possesses the ability to transmit biological, chemical, and physical signals, good biocompatibility, low operating voltage, and coupled ion-electron transport properties. Harikesh [123] et al. utilized the ion-tunable via a stepped poly(benzimidazolebenzofenophenanthroline) (BBL) to antidivergent polarity to mimic the activation and inactivation of sodium channels in biological neurons, thus realizing a conductance-based organic electrochemical neuron (c-OECN). And results demonstrated that the neuron could be used as a signal sensor to drive the nerve for signal conversion.

#### 3.3.3 FEP

FEP is a class of polymeric materials with ferroelectric properties that can be electrically polarized under the action of an external electric field. Ferroelectricity, i.e., in some dielectric crystals, the structure of the crystal cell causes the positive and negative charge centers to not overlap but an electric dipole moment occurs, generating an electrodepolarization strength that is not equal to zero, which gives the crystal a spontaneous polarization and the direction of the electric dipole moment can be changed due to the external electric field, presenting a similarity to that of a ferromagnet [124]. In ferroelectric materials, some microscopic electric dipoles are formed due to ionic excursion and electron polarization, and these electric dipoles are arranged into domains in ferroelectric materials [125]. The size and shape of the ferroelectric domains can be modulated by temperature, stress, and electric field, which have important effects on the ferroelectricity of FEPs [126].

The ferroelectricity of FEP mainly originates from the formation of dipoles pointing from one side to the other on both sides of the molecular chain by atoms or groups with large differences in polarity. FEP is highly flexible, easy to be fabricated into complex shapes, mechanically robust, and polar active [127]. Ferroelectricity in polymers was discovered in 1969 in poly (vinylidene fluoride) (PVDF), which served as a platform for efficient cross-coupling between electrical, mechanical, and thermal energies [128]. Zheng [129] et al. used the organic crystal dimethylhexynediol (DMHD) as a three-dimensional sacrificial template, which was introduced to the surface of the PVDF-based terpolymer, and when the DMHD evaporated, the polarization at the terpolymer surface interface produced a large electrothermal effect. FEPs usually include organic molecular ferroelectrics [130] and polymeric ferroelectrics represented by the PVDF family [131,132].

Although external ES can activate nerve potentials and restore bioelectrical signals in effector organs, invasive implanted electrodes are prone to cause inflammation and gliosis in damaged nerves [133]. Based on its flexibility, biocompatibility and good piezoelectric ability, FEP can be used as an alternative template for tissue-engineered biomaterials and shows great potential application in the field of nerve regeneration [134]. Orkwis [135] et al. prepared a 3D scaffold of poly (vinylidene fluoride)-trifluoroethylene (PVFD-TrFE), doped with decellularized matrix (dECM) by electrostatic spinning technique. The scaffold promoted cell adhesion and induced the expression of regenerative matrix proteins (e.g., fibronectin and laminin) for noninvasive repair of nerve injury. Cheng [136] et al. combined the biodegradable material PCL with PVDF to prepare a PCL/PVDF piezoelectric scaffold that significantly promoted the proliferation and elongation of rat SCs, and in vivo experiments also confirmed that the piezoelectric scaffold contributed to the restoration of the electrophysiology, morphology, and function of the sciatic nerve in rats. A novel nerve regeneration therapy called dynamic mechanical stimulation of FEP was found to induce electromechanical stimulation that promoted neuronal differentiation [137]. In addition, FEP could mimic the in vivo nervous system for external signal processing. Kim [138] et al. proposed a tactile neuromorphic system, and prepared a friction electrosensor based on polydimethylsiloxane (PDMS) and molybdenum disulfide (MoS2)/PVDF-TrFE heterostructure ferroelectric synapses. The system exhibitedexcellent long-term enhancement/suppression characteristics for signal recognition up to 96.17 %.

#### 3.3.4 Piezoelectric materials

Besides the aforementioned, there exist some special electrically responsive materials, although possessing ferroelectric and piezoelectric like FEP, have very different chemical properties from FEP. The voltage will appear at both ends of the crystal material when subjected to pressure for the piezoelectric material. In the year of 1880, Curie brothers found that the surface of quartz crystals could produce an electric charge, when subjected to pressure, the amount of charge and pressure was proportional to the phenomenon known as the piezoelectric effect, and the object with the piezoelectric effect was known as the piezoelectrics. Conversely, when the piezoelectrics is subjected to external electric field and deformation occurs, its deformation is proportional to the strength of the external electric field, this phenomenon is called the inverse piezoelectric effect [17].

FEP belongs to a class of organic piezoelectric materials, while inorganic piezoelectric materials also include piezoelectric crystals and piezoelectric ceramics (ferroelectric ceramics), with piezoelectric crystals generally referring to piezoelectric single crystals, and piezoelectric ceramics referring to piezoelectric polycrystals. Piezoelectric crystals are piezoelectric with the absence of a center of symmetry, such as quartz crystals [139], lithium niobate (LiNbO_3_) [140,141], and lithium tantalate (LiTaO_3_) [142,143]. Piezoelectric ceramics have strong piezoelectricity, high dielectric constant, the presence of ferroelectric domains in the grain, ferroelectric domains by the direction of spontaneous polarization reverse parallel 180 domains and spontaneous polarization direction perpendicular to each other composed of 90 domains, these domains in the artificial polarization (applying a strong DC electric field) conditions, the spontaneous polarization in accordance with the direction of the external electric field is fully aligned and in the withdrawal of the external electric field to maintain the residual polarization strength, and therefore has a macroscopic piezoelectricity [144]. Considering the biosafety, the piezoelectric ceramics commonly used in the field of tissue engineering are mainly lead-free materials, such as chalcogenides (barium titanate (BT) [145], niobium niobate (KNN) [146], sodium bismuth titanate (BNT) [147]) and some inorganic minerals [148] and metal oxides [149] nanoparticles. It should be noted that inorganic piezoelectric materials and ferroelectric polymers often complement each other in nerve regeneration applications to achieve better performance, despite their different structural properties [150,151]. Besides, natural piezoelectric biomaterials, such as amino acids [152], peptides [153], proteins [154], and polysaccharides [155], are also considered as a promising candidate in the field of tissue engineering, which could potentially replace the conventional piezoelectric materials due to their environmental sustainability and biosafety [156,157].

Piezoelectric materials are promising in the design of biomaterials for neural tissue engineering due to their property of providing on-demand, non-invasive electrical signals, which can be used to generate mechanical stress or electric field effects by applying an external pressure or electric field to affect neuronal cell morphology, differentiation and function [158]. A study explored the biocompatibility and antioxidant activity of piezoelectric nanoparticles BT and LiNbO_3_ with neuronal cell PC12, and found that both did not affect the viability, morphologic features, and reactive oxygen species (ROS) production of PC12, and also stimulated the branching of neuronal synapses [159]. In addition, combining electrical conductivity with piezoelectricity to construct conduction channels for in situ stimulation to provide support for the growth and differentiation of neural-like cells has been reported. Javidi [160] et al. developed a neural conduit with an outer shell composed of PCL/PVDF and gelatin containing PANI/graphene (GO) nanoparticles by electrostatic spinning, and the inner part contained a chitosan/gelatin hydrogel loading with ZnO and PAG nanoparticles. The conduit significantly induced the expression of nestin and MAP2 genes in PC12, which could effectively induce cell differentiation and nerve regeneration. Magnetoelectric nanoparticles (MENPs) combining piezoelectric and magnetic materials and their heterostructures had been successfully developed for applications such as neurostimulation [161], neural recording [162], etc. Bok [163] et al. established a finite element nanoscale model of cobalt ferrite (CFO) and BTO core-shell conjugate (CFO-BTO) MENPs, which were injected into neural tissues, and could act as sensors for electrophysiological recordings by quantifying imaging signals through neuronal electric fields.

The process of ES-promoted neural regeneration is exceptionally complex, and the conversion of electrical signals into biochemical cues can stimulate and modulate relevant neural regeneration processes. Cells can sense external ES through electrical coupling, but the plasma membrane prevents ES penetration due to its high electrical resistance [164]. Therefore, there are two possible mechanisms between the interaction of ES and biological systems [165], as shown in Fig. 4D, one is that the electrical coupling mechanism involves asymmetric distribution/diffusion of charged cell surface receptors in response to an electric field, which further activates many downstream signaling cascades. ERK1/2 is a member of the mitogen-activated protein kinase (MAPK) family, which can be phosphorylated and activated by growth factors, and the MAPK/ERK signaling pathway is a key component in a variety of processes, including differentiation, proliferation, migration, senescence and apoptosis in a large number of cell types [166]. It has been demonstrated [167] that ES induces pERK expression and activates ERK and p38 MAPK, and there is a strong correlation between p38 MAPK activation and neurotrophic factor-induced neuronal regeneration. Earlier studies have shown that c-Jun is a major downstream nuclear effector of the MAPK/ERK pathway [168], and also plays an important role in the regulation of inflammatory responses, axonal regeneration, and myelination, and ES also downregulates the catalytic activity of p-JNK. And previous studies reported that in a variety of cells treated with ES, the transcript levels of c-Myc, c-Fos, c-Jun and protein kinase C were elevated to different degrees under different conditions [169].

The other possible model is direct depolarization through the cell membrane by activation of voltage-gated Ca^2+^ channels, leading to a sustained cellular response to ES, Ca^2+^ inward flow, and increased intracellular Ca^2+^ concentration. In neurons, elevated Ca^2+^ concentration upregulates the levels of several neurotrophic factors (NGF, BDNF, glutamate, etc.), which activate L-type voltage-gated Ca^2+^ channels (VGCC) by altering the neuron's membrane potential, thereby inducing Ca^2+^ inward flow [170,171]. Overexpression of BDNF inhibits the phosphodiesterase enzyme that degrades cyclic adenosine monophosphate (cAMP), allowing the cells to maintain relatively high levels of cyclic adenosine monophosphate (cAMP) [170]. Thereafter, increased cAMP levels increase the expression of related genes (e.g., Tα1 tubulin), which triggers cytoskeletal remodeling and promotes axonal regeneration [172].

Although electrically responsive materials have certain advantages in nerve regeneration applications and can promote the growth and regeneration of nerve cells, they inevitably have certain limitations. Some electro-responsive materials may cause immune response and tissue damage, affecting nerve regeneration and repair, thus it is necessary to selectively use electro-responsive biomaterials and appropriately modify them for functionalization, rather than blindly pursuing electro-responsive parameters. The stimulation parameters of the materials need to be optimized and adjusted according to specific application scenarios and individual differences. Inappropriate parameters may cause damage and death of nerve cells. In addition, revealing the possible electrical response mechanisms from multiple perspectives and at multiple levels is beneficial to the development of novel biomaterials and the exploration of new therapeutic approaches for the repair and regeneration of nerve injury.

### 3.4 Mechanical stress

**Fig. 5:**
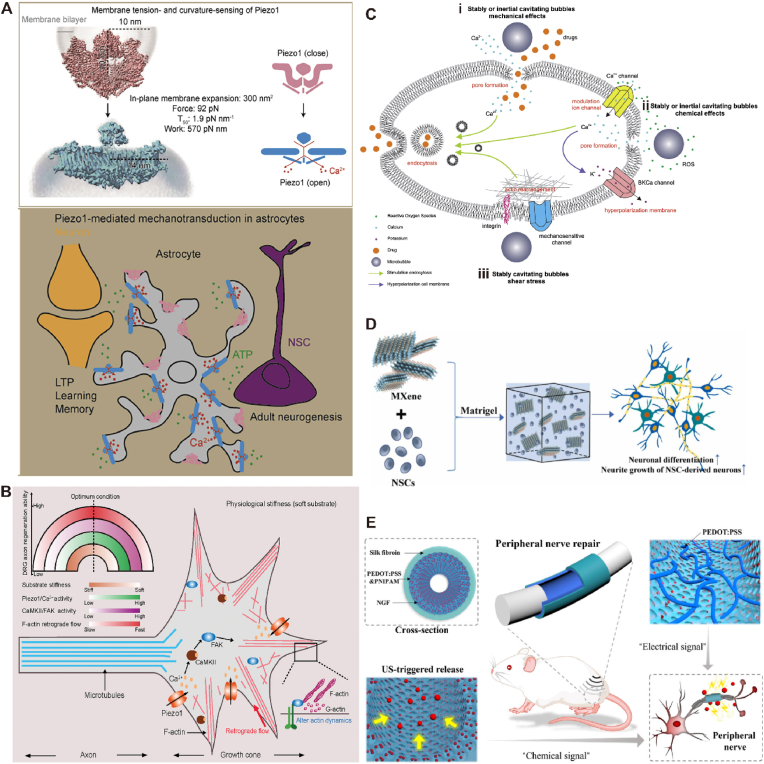
**(A)** A proposed model for the astrocyte Piezo1-mediated mechano-surveillance system in regulating adult brain functions [183]. Copyright 2022, Elsevier. **(B)** Piezo1-CaMKII-FAK-actin signaling cascade regulates DRG axon regeneration in the growth cone. A proposed model for the substrate stiffness-mediated DRG axon regrowth/regeneration through Piezo1-CaMKII-FAK-actin signaling cascade. The diagram in the top left depicts the relationship between substrate stiffness (orange), Piezo1/Ca2+ activity (green), CaMKII/FAK activity (purple), F-actin retrograde flow velocity (red), and DRG axon regeneration ability [191]. Copyright 2024, Wiley. **(C)** Biological effects of stably and inertial cavitating microbubbles [195]. Copyright 2014, Elsevier. **(D)** Conductive MXene-Matrigel hydrogels by incorporating Ti_3_C_2_Tx MXene into Matrigel for the 3D culture of NSCs [210]. Copyright 2024, Elsevier. **(E)** Schematic diagram of PEDOT:PSS composited conductive silk conduit with US-triggered NGF delivery ability for peripheral nerve regeneration [212]. Copyright 2023, Wiley.

Methods such as grinding or pulverization have been recognized as a means of accelerating chemical reactions for more than two thousand years [173]. Until 2005, polymer force chemistry entered a new period when a molecular device was developed, which could selectively activate weak bonds in the polymer backbone in response to mechanical forces [174]. Mechanical stress-responsive materials allow for the conversion from mechanical to chemical energy and even other forms of energy to produce specific responses, including deformation, color change, electrical conductivity, and substance generation [175]. Piezoelectric materials also belong to one of these categories, as described in 3.3.4. Numerous studies have reported that the elasticity of biomaterials affects the growth of cells and tissues [176]. For example, Cheng [177] et al. found that structural protein responses, neurite extension, and protein distribution varied between substrates by culturing DRG on polydimethylsiloxane (PDMS) substrates with different elasticities, as shown in Fig. 5A. The cellular response to morphology-mediated physical guidance can be attributed to changes in the cytoskeleton and signaling pathways, which are generally thought to be closely related to integrin adhesion molecules, which are adhesion molecules widely distributed on the surface of cell membranes and play a role in signaling the mechanical properties of the extracellular matrix to the cell during its adhesion to the extracellular matrix [178,179]. Integrins have been reported to be found in the growth cones of adhesion spot proteins, and activated integrins produce adhesion spots (FA) that induce cytoskeletal assembly, thereby altering cell morphology [180,181]. And there is evidence that upregulated adhesion patch kinase (FAK) expression can participate in a series of signaling cascades to regulate cellular function and promote neural regeneration [182].

Not only do external materials respond to mechanical stress, but in organisms, the Piezo channels located in cell membranes also sense mechanical stimuli such as poking, stretching, shear forces, substrate stiffness, and endogenous cellular tugging forces. Chi [183] et al. revealed that astrocytes could utilize Piezo1 channel-mediated mechanotransduction mechanisms, effectively regulating ATP-dependent hippocampal adult neurogenesis and cognitive function. In addition, since nerve injury disrupts the local mechanical microenvironment, inspired by the dynamic properties of the natural ECM, cell-adaptive hydrogels formed by reversible cross-linking respond to cell-generated forces and remodel the cytoplasmic matrix, which can help to promote cell proliferation, migration, differentiation, and paracrine effects [184]. Gao [185] et al. utilized the dynamic interaction between bisphosphonate-modified alginate (Alg-BP) and Mg^2+^ to co-construct a cell-adaptive hydrogel with SF, in which, the dynamic network structure could respond to cell-generated forces for cell-mediated remodeling. The hydrogel was found to recruit macrophages for M2-type transformation, accelerate migration and axon elongation of SCs, and thus promote nerve regeneration and recovery from muscle atrophy.

The process of nerve regeneration is influenced by genetic cues and environmental signals. The growth cones can bind to substrates present in the environment and respond to secreted nutrient molecules for directing axon growth. At the cellular level, growth cones are highly sensitive to changes in Ca^2+^ concentration, and the spatial localization and amplitude of Ca^2+^ can influence growth cone extension and neurite growth [186]. At the molecular level, diffusible membrane-tethered ligands and their corresponding membrane receptors direct axon growth and dendritic branching [187]. Netrin-1 [188], neurotrophins, and signaling hormones [189] can regulate the dynamic levels of Ca^2+^ in growth cones, direct axonal recognition of synapses, and promote nerve regeneration. Li [190] et al. demonstrated that inhibition of DEG/ENaC channels led to reduced dendritic growth and branching in vivo, whereas overexpression of the mechanosensitive channels PEZO-1/Piezo or YVC1/TrpY1 alleviated this phenotype. During growth, filopodia were subjected to mechanical forces generated by ligand-receptor interactions, which activated the mechanosensitive DEG/ENaC, and triggered transient localized Ca^2+^ influx through L-type VGCC. These localized Ca^2+^ transients further stimulated the continued growth of dendritic branches. Interestingly, excessively high levels of Ca^2+^ activity could paradoxically inhibit axonal extension, a process still mediated by Piezo ion channels. As shown in Fig. 5B, these channels primarily detected substrate stiffness at the growth cone, facilitating Ca^2+^ influx that activates the downstream CaMKII–FAK–actin signaling cascade to coordinate axonal regeneration. Notably, CaMKII and FAK exhibited dual roles in axonal regeneration, depending on their respective activity levels [191]. Song [192] et al. proposed an alternative Piezo-mediated signaling pathway regulating axonal regeneration. During this process, mechanical stimulation activated Piezo channels, triggering localized Ca^2+^ elevation in the growth cone and a Nos-mediated signaling cascade. This subsequently activated the Atr-Chek1 pathway, inhibited Cdc25, and ultimately suppressed axonal regeneration. The above studies suggest that the restorative effect of Piezo on nerve regeneration is two-sided, as reflected by Ca^2+^ expression, and that there exists an optimal activity level interval, below/above which leads to negative regulation of nerve regeneration.

Furthermore, ultrasound (US), a common mode of force application in polymer force chemistry, is promising in the field of neuroregenerative medicine due to its non-invasive nature, non-ionizing radiation, high tissue penetration depth, and spatio-temporal controllability [193,194]. There are two main methods of US-induced mechanical effects: one is the stable cavitation produced by continuous oscillation of microbubbles; and another one is the inertial cavitation produced by rapid growth and rupture of microbubbles [195,196], such as shown in Fig. 5C. Fluid shear triggered by continuous oscillations of microbubbles can disrupt the carrier to release drugs, while at the same time, a certain amount of shear will form transient pores in the cell membrane, increasing membrane permeability and thus enhancing drug uptake [197]. The collapse of inertial cavitation microbubbles generates an instantaneous shock wave greater than atmospheric pressure, which in turn affects the structural stability of the carrier and leads to drug release [198]. Significantly, the tensile flow forces generated by this cavitation effect can also lead to the breakage of chemical bonds with low bonding energies, such as disulfide (S-S) [199], peroxide (O-O) [200], and a variety of ligand bonds [201,202], which result in the transformation of the chemical structure of the drug molecule, and thus can be used to modulate the activity of the drug molecule [203].

US not only triggers mechanical effects, but also thermal effects. The thermal effect is produced by that when ultrasonic waves propagate in a medium, part of the acoustic energy is absorbed by the medium and converted into thermal energy [204]. There are two thermal effects: one is the continuous thermal effect generated by the continuous oscillation of cavitated microbubbles; the other is the transient thermal effect triggered by the collapse of cavitated microbubbles resulting in localized overheating [205].The generation of the thermal effect of US facilitates the release of drugs from carriers containing heat-sensitive materials, such as temperature-sensitive liposomes [206,207], polymer micelles [208], and hydrogels [209], etc.

Mechanical force-responsive materials have a wide range of R&D potential in neural regeneration, which can directly or indirectly affect the growth and development of neural cells through mechanical signals. MXene is a novel 2D nanomaterial with high electrical conductivity and flexibility that makes it a strong candidate for pressure-responsive materials. MXene was doped into Matrigel hydrogel to prepare 3D conductive hydrogel scaffolds (Fig. 5D), and MXene could well improve the mechanical properties of hydrogel, provide an electrical microenvironment for NSCs, and promote the proliferation and differentiation of NSCs [210]. In addition, Ti_3_C_2_T_x_ MXene was found to promote the differentiation of neural stem cells into neurons and astrocytes, and the synergistic interaction with aligned PLLA nanofibers enhanced neuronal maturation and guided cell growth to improve neural regeneration [211]. Zhang [212] et al. developed a hydrogel containing (PEDOT: PSS), poly(N-isopropylacrylamide) (PNIPAM), and NGF in a composite conduit with an anti-opaline structure. The US triggered an increase in temperature, and PNIPAM was temperature-sensitive and received signals to produce contraction, enabling controlled release of NGF (Fig. 5E). In vitro PC12 cell culture and in vivo animal experiments showed that the composite catheter had a positive effect on the regeneration of the peripheral nervous system. Xu [213] et al. encapsulated delivered lidocaine in PLGA microcapsules, and used ultrasound as a triggering switch could promote the rapid release of lidocaine from the microcapsules. The in vitro and in vivo experiments confirmed that the microcapsules could achieve the dual effects of long-term slow release and short-term ultrasound-triggered rapid release, realizing a new type of on-demand drug delivery. In vitro and in vivo experiments demonstrated that the microcapsules could achieve both long-term sustained release and short-term ultrasound-triggered rapid release, realizing a novel on-demand drug delivery. In addition, based on ultrasound's characteristics of deep tissue penetration, high spatial resolution, and high safety, the ultrasound response can also act synergistically with electrical stimulation as an effective energy source for excitation of implantable electrodes [214].Chen and Wang [215] developed a hydrogel nanogenerator (HENG) based on a polyacrylamide/GO conductive hydrogel. This stimulator generated alternating current in response to ultrasound-induced vibrations, and the current frequency and waveform could be modulated by ultrasound pulses. Using endotoxin-induced systemic inflammation as a model, radio stimulation of the vagus nerve by the ultrasound-responsive HENG effectively inhibited pro-inflammatory factors. Pi and Rao [216] et al. developed an acoustic-mechanical electrotherapeutic system combining ultrasound-activated bioelectrical modes with biomechanical modes based on a unidirectional nano-topography, and used electrostatic spinning to prepare PCL/PVDF nanofibrous scaffolds, which significantly promoted SCs growth in In vitro, and significantly promoted the function and neuronal growth of SCs, and in vivo, the scaffolds successfully restored the motor function of 15 mm sciatic nerve in rats.

The prevailing mechanism of US effects on regeneration-associated cells is the oscillatory pressure wave acting on the lipid bilayer of the cell membrane, known as the bilayer sound carrier (BLS) theory, where the oscillatory wave acts alternately on the membrane and further on mechanosensitive ion channels embedded in the membrane, altering their gating kinetics and leading to ion influx [217]. This mechanism is referring to the inertial cavitation effect induced by ultrasound, which leads to transient pore opening and affects cell shape and function [218,219]. It has been demonstrated [220,221] that this mechanism positively mediates the secretion of neurotrophic factors, such as BDNF and NGF from SCs, which promote axonal regeneration and myelin production. In addition, single or long-term US has been shown to attenuate the inflammatory response during nerve regeneration by inhibiting the release of inflammatory cytokine expression (TNF-α and IL-1β) from the spinal cord as a means of attenuating the inflammatory response [222]. And US has also been found to block the upregulation of TNF-α and IL-6 expression and promote IL-10 expression in rat sciatic nerve, effectively relieving neuropathic pain [223,224].

Since various materials may have different mechanical response properties, such as stiffness, modulus of elasticity, tensile strength, etc. Therefore, suitable mechanical response parameters need to be selected according to the specific neural tissue or cell type, and fully adjusted and optimized in the application. The application of some mechanical force-responsive materials requires special experimental conditions and equipment support, such as tensile equipment, pressure transducers, ultrasonographs, etc., which increases the complexity and cost of experiments. Generally, the mechanical response is used in combination with the non-invasive stimulus response to optimize the experimental conditions, which can better achieve various experimental purposes.

### 3.5 Light

Light is critical in the living world and plays an important role in the survival and adaptation of organisms, which utilize it extensively for a variety of biological processes. Plants use photosynthesis to convert light energy into chemical energy and synthesize organic substances. Insects use light signals to perform behaviors such as courtship, warning and predation. Light stimulation is characterized by clean and easy accessibility, high sensitivity, remote manipulation, and precise and rapid response modulation [225]. Therefore, scientists have also shifted their focus to light for developing various types of smart light-responsive biomaterials for human life applications. Light-responsive materials convert light stimuli into chemical reactions, sound, light, heat, electricity, force and other forms of energy through photochemical or photophysical mechanisms [226]. The properties of the materials, such as antimicrobial properties [227], mechanical properties [228], adhesion [229], cross-linking degree [230], solubility [231], and biodegradability [232,233], can be altered by different photoresponses or by adjusting the parameters of the light (e.g., wavelength, intensity, etc.). The above properties make photoresponsive materials very promising for a wide range of applications in the field of neural regeneration, and the two main types of response are described below.

#### 3.5.1 Photothermal materials

**Fig. 6:**
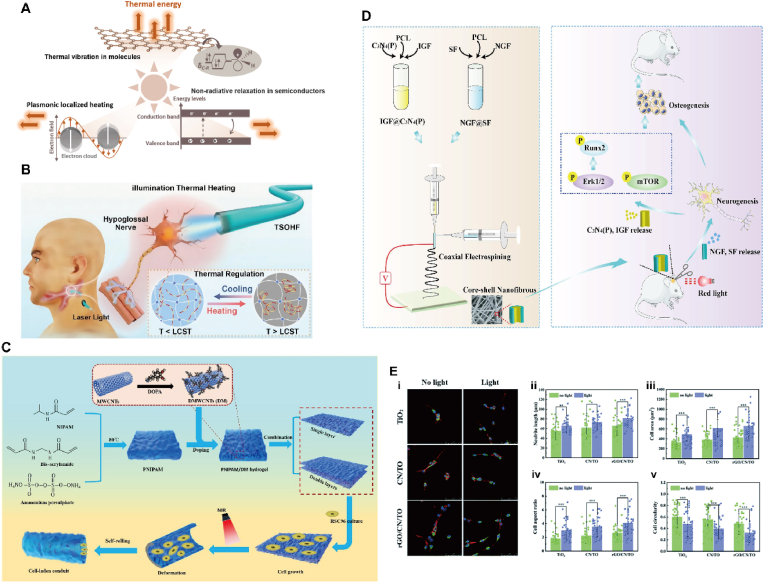
**(A)** Different mechanism of photothermal effect [234]. Copyright 2022, Elsevier. **(B)** Concept of the thermoregulation during optogenetic neuromodulation using temperature-sensitive semi-interpenetrating optical hydrogel fiber (TSOHF) [251]. Copyright 2023, Springer Nature. **(C)** Sketch map of the preparation of photothermal responsive cell-laden PNIPAM self-rolling hydrogel containing dopamine enhanced MWCNTs [50]. Copyright 2023, Elsevier. **(D)** Schematic diagram of red light + IGF@C_3_N_4_(P)/NGF@SF scaffold fabrication, and their bone defect repairment utilizations and molecular mechanism in vivo [257]. Copyright 2024, Elsevier. **(E)** (i) Immunofluorescence images of PC12 cells on the various nanocoatings with or without visible-light stimulation, and the cell staining: F-actin with TRITC phalloidin (red), vinculin with anti-vinculin–FITC antibody (green), and nuclei with DAPI (blue). Analysis results of (ii) neurite length, (iii) cell area, (iv) cell aspect ratio, and (v) cell circularity of PC12 cells on the different nanocoatings with or without visible-light stimulation [260]. Copyright 2022, RSC.

The photothermal effect produced by photothermal materials refers to the phenomenon of heat generation by materials under light irradiation, through which the photothermal effect can not only maximize the energy conversion efficiency by adequately combining light and thermal stimuli, but also break the limitations of the materials in the temporal and spatial dimensions. The conversion mechanisms of photothermal materials include plasma localized heating, non-radiative relaxation, thermal vibration of molecules, and their composite effects (Fig. 6A) [234]. Existing photothermal materials can be categorized into two kinds, inorganic materials such as: carbon-based materials [235] (GO, carbon nanotubes (CNTs)), metallic nanoparticles [236,237] (Au, Ag), metallic and non-metallic compounds (Ti_2_O_3_ [238], MoO_3_ quantum dots [239], CuS [240]), etc., organic materials such as: organic dyes [241] (perylene diimide derivatives, elastin dyes), organic framework materials (MOF [242], COF [243]), organic polymers (polydopamine (PDA) [244], PPy [245], PANI [246]), and some other complexes [247].

It has been reported that the local temperature increase triggered by photothermal materials can stimulate the activity of nerve cells, which in turn leads to the effect of nerve repair. Li [50] et al. constructed a photothermal-responsive self-curling hydrogel scaffold (Fig. 6C) by surface modification of MWCNT and polymerization with PNIPAM. The scaffold deformed to form tubular-like structure under NIR irradiation, promoted the growth of SCs, induced the release of nerve growth factor, regulated the expression of myelin-related genes, and promoted peripheral nerve regeneration. In addition, photoresponsive combined with thermal stimulation can be used to develop non-invasive neuromodulation techniques. PDA nanoparticles have a wide range of light-absorbing wavelengths and excellent light-absorbing properties, and they are also highly biocompatible and sustainably biodegradable for use in neural regeneration. Derami [248] et al. used PDA nanoparticles as a photothermal nano-transducer, and by varying the excitation power density and irradiation duration, they can finely regulate the activity of nerve cells and achieve spatial localization of photothermal stimulation. Based on the advantages of fast response, easy handling and non-invasiveness of photothermal materials, photothermal therapy has emerged for the treatment of neurodegenerative diseases such as Alzheimer's disease (AD) and Parkinson's disease (PD), etc. Yuan and Jia [249] et al. synthesized ruthenium nanoclusters loaded with both NGF and RVG through the -Se-Se-bonding, and the nanoclusters showed good photothermal properties, which could effectively inhibit the aggregation of β-amyloid (Aβ), deliver NGF to the brain, repair damaged nerves and promote neuronal regeneration, thus alleviating AD symptoms. In addition, the use of optogenetics for the regulation, repair and treatment of various neurological diseases has been developed in recent years. It has been shown [250] that optogenetics can improve synaptic density/length and synaptic plasticity and promote axonal regeneration of neurons through the activation of astrocytes. Chen [251] et al. prepared a temperature-sensitive semi-interpenetrating optical hydrogel fiber (TSOHF) by wet spinning as shown in Fig. 6B, which possessed tunable temperature sensitivity, excellent light propagation properties and good biocompatibility, and could be utilized for optogenetic modulation and repair of the hypoglossal nerve.

#### 3.5.2 Photovoltaic materials

Photovoltaic materials convert light energy into electrical energy by forming hole-electron pairs on the surface of the material through the photovoltaic effect, which mainly include inorganic molecules such as silicon, metals and their oxide nanoparticles, chalcogenides, etc. It has been demonstrated [252] that the photovoltaic conversion efficiency (PCE) of lead-free doped chalcogenide solar cells based on Bi_0.8_La_0.2_FeO_3_ can be up to 5.61 %. Organic molecules such as pyrene, carbazole, triphenylamine, fluorescein, phosphine oxide, squaracinine, and dithienothiazole have been widely studied in the field of photovoltaics [253]. In order to further modulate the physicochemical properties of photovoltaic materials, they can be improved by various methods including metal ions dopping, chemical bonding, crystallization, and aggregation induction. In addition, peptide-based materials have also been applied in the field of photovoltaics due to the self-assembly properties and biocompatibility of peptide derivatives, as well as photosensitivity and electroactivity [254].

Common photoactivated materials require short-wavelength light excitation and are accompanied by poor tissue penetration and biohazards. Qiao [255] et al. constructed a p-n heterostructured Bi_2_S_3_/TiO_2_/rGO (BTG) nanoparticles, which provided interfacial adhesion and electron-transfer pathways to matrix materials, and modulated electrophysiology by coupling endogenous electric field (EEF) under visible light irradiation and regenerative microenvironment under visible light irradiation, thus promoting nerve regeneration. Carbon nitride (C_3_N_4_) nanosheets can generate two-photon induced fluorescence via red and blue light excitation due to their conjugated Π-bonds and the strong effect of amino groups to release electrons for electron transfer, thus generating electric current [256]. Wang [257] et al. prepared a nanofibrous electrospun scaffold with with phosphorus doped g-C_3_N_4_ as the core, and under red light stimulation, the scaffold induced BMSCs to promote the high expression of nestin and β-microtubulin III (Fig. 6D) in neural stem cells and induced them to be differentiated into neurons, stimulating axon elongation and favoring neurogenesis. In addition, it has been shown that rGO-coupled g-C_3_N_4_ composites can improve electron transfer and promote neural axon elongation under light irradiation [258]. rGO and TiO_2_ heterostructures act as photoelectric stimulators can effectively promote neural stem cells to neuronal differentiation [259]. Based on this, Yan [260] et al. used g-C_3_N_4_ as a photocatalyst, rGO as a conductive interface, and added a TiO_2_ coating, which not only enhanced the visible light-responsive photoelectric performance, but also significantly promoted the neuronal growth and differentiation of PC12 cells (Fig. 6E).

The light response in organisms originates from photosensitive proteins (retinal proteins), a class of membrane proteins on cell membranes that can sense light stimulation at a certain wavelength and produce specific effects, which are categorized into activating and inhibitory types, and are capable of causing neuronal excitation or inhibition. The more widely used retinal-2 (ChR2) photogated cation channel that can be activated by blue light at 475 nm permits a large inward flow of cations (Na^+^, Ca^2+^, etc.), generating an action potential and placing the neuron in an excitatory state [261]. Moreover, optogenetic technology based on photosensitive proteins, which combines optical and genetic approaches, allows precise spatial and temporal control of specific cell populations in response to different wavelengths of light stimulation to achieve modulation of neural function by utilizing ion channels to selectively regulate cellular excitability [262]. For example, Lee [263] et al. used the pan-neuronal promoter synaptophysin-1 (SYN1) and the excitatory neuron-specific promoter calmodulin kinase II (CaMKII) to express ChR2, and achieved non-invasive and precise control of neural networks via a hydrogel 3D system.

However, light-responsive materials may require high light stability, and changes in light exposure time and intensity reduce the responsiveness of the materials. Some light-responsive materials can only modulate shallow layers of tissue, making it difficult to penetrate into the tissue with high depth. The instability and limited light penetration depth limit the application scope of light-responsive materials in the field of nerve regeneration. Therefore, it is necessary to chose a suitable light source, and reasonably design and improve the light source to increase its light intensity and light penetration depth, thus to achieve the modulation of deep tissues. In addition, the precise regulation and optimization of the nerve regeneration process may be effectively achieved by means of combining materials with multiple functions and investigating novel materials for application in three-dimensional tissue engineering.

## 4 Endogenous signals

**Fig. 7:**
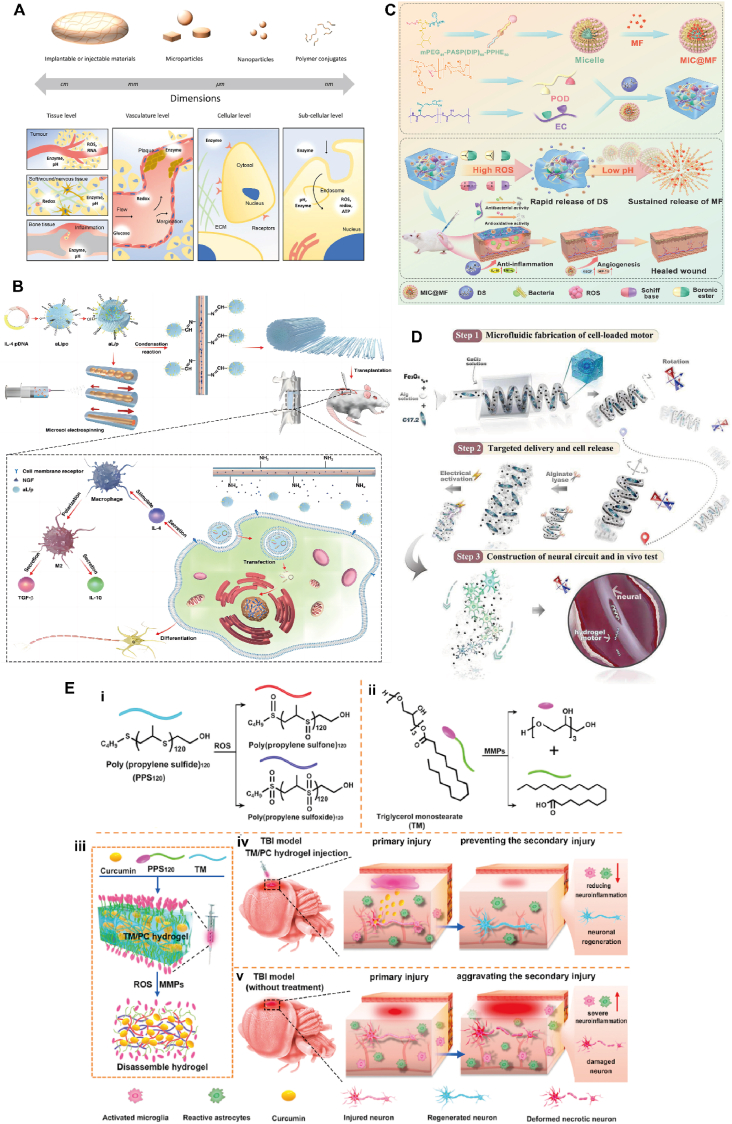
**(A)** Schematic showing design synergies between materials' physiochemical properties and biological environments. Biomaterials varying in shape and size from centimeters to nanometers can be applied in tissue/cell environments having structural features across similar length scales, while also expressing various cues able to be recognized by endogenous stimuli-responsive materials (including enzymes, pH, redox, glucose, hypoxia, ATP, and nucleic acids) [276].Copyright 2021, ACS. **(B)** Scheme illustration of i the construction of bioinspired composite scaffold for the treatment of spinal cord injury along with ii its microenvironment-responsive immune regulation and nerve regeneration effect [280].Copyright 2020, Springer Nature. **(C)** (i) Schematic diagram of the fabrication procedures of the DS&MIC@MF embedded POD/CE hydrogels. (ii) Illustration of spatiotemporally drugs release behavior of the hydrogel, and the mechanism of the hydrogel for accelerating wound healing on the infected diabetic cutaneous wound model [282]. Copyright 2022, Elsevier. **(D)** A Schematic illustration depicting the fabrication process of helical hydrogel micromotors loaded with neural stem C17.2 cells and their application in nerve regeneration. C17.2 cells and Fe_3_O_4_ nanoparticles were encapsulated in the helical alginate hydrogel via a microfluidic approach (Step 1). The cell-loaded motor demonstrated corkscrew motion to the targeted site under a rotating magnetic field and released cells (Step 2). The interconnection of neural cells was reconstructed in vitro/vivo (Step 3) [286]. Copyright 2024, Elsevier. **(E)** Formation and mechanism of the TM/PC hydrogel in TBI. (i) PPS120 switched from a hydrophobic polymer to the more hydrophilic poly (propylene sulfone)120 and poly (propylene sulfoxide)120 in the ROS environment. (ii) TM could be cleaved by MMPs. (iii) Schematic illustration of TM/PC hydrogel preparation procedures and their degradation process under post-trauma MMP and ROS conditions. TM could self-assemble into a hydrogel, and PPS120 and Cur could be encapsulated within the hydrophobic core of TM lamellae to form the TM/PC hydrogel. (iv) In situ injection of TM/PC hydrogels within the postsurgery TBI: TM is degraded, and PPS120 scavenges ROS to release Cur, reducing neuroinflammation and the secondary spread of injury. (v) Without hydrogel treatment, neuronal death and severe neuroinflammation were observed, and the secondary injury was aggravated [293]. Copyright 2021, Elsevier.

Endogenous stimulus response materials with enhanced response sensitivity and spatiotemporal control have received a lot of attention in the development for more effective and precise therapeutic systems [264]. Endogenous stimulus response materials are able to respond to stimuli generated within organism, and their changes can reflect the internal state and function of the organism. As shown in Fig. 7A, systems that respond to internal stimuli can be designed on length scales from nanometer to macroscopic and can respond to endogenous signals by incorporating synthetic biomimetic or natural components such as pH [265], enzymes [266], glucose [267,268], adenosine triphosphate (ATP) [269], spent oxygen [270], reactive oxygen species (ROS) [271,272], redox [273,274], and nucleic acids [275], which are associated with the microenvironment of cells and tissues [276].

pH is a measure of the concentration of hydrogen ions. In living organisms, changes in the pH of intra- and extracellular fluids can affect cellular metabolism and functions, such as enzyme activity, protein structure, and cell membrane permeability. Therefore, the study of pH-responsive materials is also of great importance in the field of tissue engineering. pH-responsive groups, including pyridine, carboxylic acid, phosphate, sulfonic acid and tertiary amine, are susceptible to ionization caused by changes in the pH of the surrounding medium. Common pH-responsive materials include polyelectrolytes, nanoparticles, hydrogen-bonded polymers, and natural macromolecules (gelatin, chitosan, and alginate), which can undergo physical and chemical changes such as swelling, shrinking, degradation, or membrane fusion and rupture upon pH changes [277].

It has been demonstrated [278,279] that pH-responsive materials can be used to construct drug delivery systems to achieve targeted release of drugs required for regenerating tissues. Xi et al. [280] inspired by the inflammatory and acid-enriched features of the microenvironment after acute SCI, grafted aldehyde-modified cationic liposomes loaded with IL-4 plasmid (pDNA) onto the surface of a fibrous scaffold via Schiff base bonds. The scaffold fractured in an acidic environment, releasing pDNA to promote NGF secretion from neuronal cells, thereby promoting CNS regeneration (Fig. 7B). Since the acute and transient inflammatory response after injury is critical for effective tissue regeneration [281]. Therefore, there are some pH-responsive materials that do not act directly on nerves, but are expected to be used for nerve repair by promoting indirect effects such as inflammatory response and angiogenesis. For example, Wu [282] et al. constructed a pH/ROS dual-responsive injectable glycopeptide hydrogel based on phenylboronic acid grafted oxidized dextran and caffeic acid grafted ε-polylysine, and encapsulated anti-inflammatory drugs and nanomembranes carrying pro-angiogenic drugs in a hydrogel (Fig. 7C), which can realize spatio-temporal controlled release of the drugs, and has good antioxidant, anti-inflammatory, and angiogenesis promoting properties. Liu [283] et al. constructed a composite biomaterial to generate pH-responsive hydrogels by loading formyl-methyl-leucine-phenylalanine (fMLP) and FasL-conjugated silica nanoparticles (SiO_2_-FasL). Gel@fMLP/SiO_2_-FasL enabled fMLP burst release to rapidly recruit neutrophils to enhance inflammation, followed by the timely release of SiO_2_-FasL to induce neutrophil apoptosis and subsequent macrophage conversion to an anti-inflammatory phenotype, thereby alleviating inflammation and driving regeneration.

pH-responsive materials inevitably change the pH of tissues/cells, and how do cells respond while the materials respond to pH changes? In 2017, Zhang et al. discovered for the first time how cells respond to changes in the external environment and maintain important cytological events in intracellular metabolic homeostasis by capturing changes in intracellular pH [284,285]. It was demonstrated that Smad5, a transcription factor located downstream of the classical BMP signaling pathway, sensed changes in temperature, extracellular pH and osmotic pressure. High temperature, extracellular acidification and hypoosmotic conditions induce Smad5 to accumulate in the nucleus, whereas under low temperature, extracellular alkalization and hyperosmotic conditions, Smad5 was rapidly translocated from the nucleus to the cytoplasm. Furthermore, ablation of Smad5 led to chronic and irreversible dysregulation of cellular bioenergetic homeostasis and disrupted normal neurodevelopmental processes [284].

Stem cell therapy is a hot spot for nerve injury and neurological disorders. However, the current stage of technology is limited in terms of cell implantation accuracy and neuronal connectivity restoration, etc. Peng et al. [286] proposed an alginate hydrogel micro-nanomotor prepared with microfluidics, which encapsulated neural stem cells and magnetic nanoparticles (Fig. 7D). Alginate is enzymatically responsive and can be degraded by alginate lyase [287]. The nanomotor can be localized and released by an external magnetic field for precise delivery of neural stem cells and activation of intercellular signaling pathways to promote the repair of functional neural interactions in vivo while maintaining their ability to differentiate. Wang et al. [20] prepared a poly(3-hydroxybutyrate-co-3-hydroxyvalerate) (PHBV) nanofibrous scaffold, which contained adipose-derived stem cell (ASC) secreted exosomes and transplanted them into a sciatic nerve injury model. miR-218 enhanced the regulation of ASC differentiation, and the secreted exosomes showed different functional differences in response to different concentrations of CO_2_ stimulation. The results showed that low CO_2_ stimulation of responsive exosomes enhanced PC12 cell activity and promoted regeneration of damaged sciatic nerve.

Differences in redox potentials exist at both tissue and cellular levels, e.g., glutathione/glutathione disulfide has been shown to be the most abundant redox pair in animal cells [288]. ROS are a class of highly reactive oxidizing substances, including superoxide anion (O^2−^), hydrogen peroxide (H_2_O_2_), and hydroxyl radical (-OH), and when the intracellular environment is changed, the enzyme systems of these activity will increase, resulting in an enhancement in ROS production, on the other hand, there are some antioxidant systems such as superoxide dismutase (SOD), glutathione peroxidase (GPx), and glutathione-S-transferase (GST), etc., which are capable of scavenging ROS and maintaining intracellular redox homeostasis [289]. The ROS production and scavenging systems actively maintain the intracellular oxidative reduction state and mediate redox signaling, whereas the overproduction of ROS in inflamed tissues further worsens local tissue damage and causes chronic diseases [290,291].The production of ROS is pleiotropic and persistent, as well as bringing about a series of pathological chain reactions, which have a great impact on the repair of damaged nerves. Therefore, scavenging ROS and inhibiting the inflammatory response of nerve tissues through effective antioxidant interventions for the purpose of protecting nerve tissues has become an effective strategy for the treatment of nerve injuries nowadays [292]. Qian [293] et al. developed a microenvironment-responsive injectable hydrogel (Fig. 7E), which responds to the post-traumatic inflammatory environment of matrix metalloproteinases (MMPs) and ROS, allowing for durable drug delivery. In vivo experiments have also demonstrated that the hydrogel is effective in promoting nerve regeneration after TBI.

Autophagy constitutes a necessary line of defense as a means for cells to cope with oxidative stress (e.g., bursts of high levels of ROS) [294]. Not only oxidized/damaged proteins, but also a large number of ROS-producing organelles (e.g., mitochondria and peroxisomes) can be removed to limit further ROS production. Direct thiol oxidation of key proteins, such as ATG4, ATM, and TFEB, is responsible for specific regulation of phagocytic vesicle expansion, cargo recognition, and autophagy gene transcription, respectively [295,296]. Meanwhile, oxidation of certain redox-sensitive analogous chaperone proteins (e.g., PRDX family members and PARK7) may impair the nonspecific localized reducing environment at the phagosome membrane and affect phagosome nucleation and mitotic recognition involving BECN1, which provides another antioxidant mechanism by SQSTM1 when autophagy is unavailable or impaired [297].

Endogenous stimulus response signals are better biocompatible and biologically safe compared to external stimuli, can trigger different physiological effects depending on the signal, and have better specificity and selectivity, which are sustainable/periodic for better regulation of physiological functions. Unfortunately, materials sensitive to endogenous signals suffer from inconsistencies in target biological parameters across pathological states and between individuals, and this significant inter- and intra-individual variability hinders the application of endogenous signal-responsive materials. Currently, endogenous stimulus response signals are more often applied in the fields of vascularity, bone regeneration, and tumor therapy in tissue engineering, and research on their application for neural regeneration is rarely reported. The development of new endogenous signal-responsive materials that are easy to regulate may be expected to break through the bottleneck of smart materials at the current stage, reduce the organism's rejection reaction and side effects to the materials, and realize multiple regulation of the nerve regeneration process, which is conducive to long-term treatment and repair of peripheral nerve injury.

## 5 Multiple response signals

**Fig. 8:**
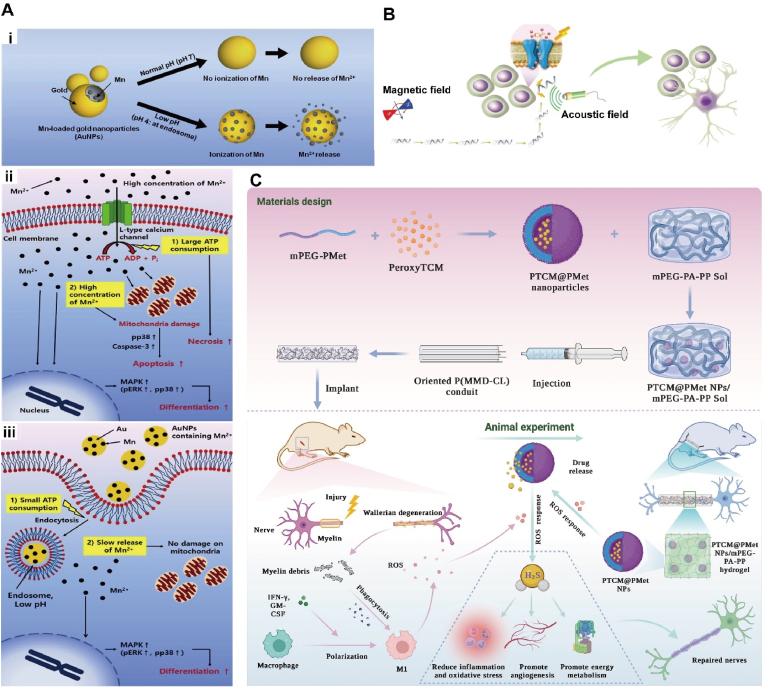
**(A)** Schematic diagram of (i) MnAuNP that can release Mn^2+^ in pH-dependent manner and (ii) conventional free Mn^2+^ uptake by PC12 cells versus (iii) the pH-responsive, intracellular delivery of Mn^2+^ using MnAuNPs for the neuronal differentiation of PC12 cells. (ii) Addition of a high concentration of free Mn^2+^ to the culture medium induces the neuronal differentiation of PC12 cells but results in necrosis caused by the abrupt consumption of a large amount of ATP for the intracellular transport of a high concentration of Mn^2+^ through the ion channels of the cell membrane and in apoptosis due to mitochondrial damage caused by the high intracellular concentration of Mn^2+^. (iii) The addition of an equal concentration of Mn-incorporated AuNPs to the culture medium not only induces the neuronal differentiation of PC12 cells but also prevents necrosis by the consumption of a smaller amount of ATP for the intracellular delivery of MnAuNPs by endocytosis, and apoptosis by releasing Mn^2+^ from the MnAuNPs in the endosomes of the cells in a sustained manner [301]. Copyright 2015, Elsevier. **(B)** Schematic illustration of a highly controllable micromotor to induce the differentiation of the targeted neural stem-like cell [306]. Copyright 2020, Wiley. **(C)** Schematic illustration of constructing an intelligently responsive multi-effect messenger biomimetic nerve scaffold for PNI repair. (Ischemia and inflammation after PNI will cause many ROS, which will trigger the release of H_2_S donors from the H_2_S functional system and further H_2_S production. The pleiotropic effects of H_2_S include anti-inflammatory, anti-oxidative, angiogenesis, and mitochondrial function repair, accelerating the reconstruction of the microenvironment in SD rats' injured nerve regeneration, axons' growth, and motor function recovery after trauma. IFN-γ, interferon-γ; GM-CSF, granulocyte-macrophage colony-stimulating factor.) [307] Copyright 2023, AAAS.

Certain materials inherently have multiple response properties, e.g., photothermal effect, electroactivity and pH response in GO [298], photothermal conversion, electrical conductivity and redox activity in PDA [299], and electroactivity, photothermal properties and photo-mechanical properties in gold nanoparticles (Au NPs) [300], etc., and thus can play different roles in various smart responsive systems. For example, Bhang [301] et al. designed a pH-sensitive Mn^2+^-loaded AuNPs in Fig. 8A. Mn has been shown to influence neuronal differentiation and has a high affinity for calcium-binding molecules, such as calmodulin [302]. The low intracellular pH conditions in PC12 cells after uptake of the Mn-AuNPs can trigger the in-situ release of Mn^2+^ that is used in neural development and promotes neuronal differentiation. In another study, AuNPs were used as a medium to induce light-based neurostimulation through the photothermal effect of localized surface plasmon resonance (LSPR) [303]. Yuan [304] et al. irradiated NGF-functionalized superparamagnetic iron-gold-oxide nucleoshells (NGF-SPIO-Au) NPs using low-intensity LEDs (<2 mW/cm^2^) on PC12 cells, by integrating the magnetic properties of SPIO and the LSPR properties of Au, significant enhancement of neuronal differentiation (83 %) and neurite growth (51 %) was observed, as well as significant upregulation of β3-microtubulin and integrin β1.

In addition, the local regenerative microenvironment of nerve injury/lesion is unbalanced and complex, and it is difficult for a single responsive condition to cross multiple biological barriers to reach the designated localization sites and perform the corresponding functions to meet the needs of the nerve regenerative environment. Generally, designing hierarchical targeting strategies to prepare smart-responsive material systems with multilevel responsiveness is more promising to achieve a more precise response to external stimuli as well as to obtain properties that are more compatible with the internal environment [305]. Liu [306] et al. prepared a magneto-dynamic helical micromotor by sequentially coating Fe_3_O_4_ and BaTiO_3_ nanoparticles on Spirulina platensis by electrostatic adsorption, as shown in Fig. 8B. Fe_3_O_4_ induced the micromotor to autonomously move to the target PC12 cells under a low-intensity rotating magnetic field, and then ultrasonic energy was subsequently converted by BaTiO_3_ into electrical signals to induce the differentiation of the target neural cells through BaTiO_3_. This stimulation method has high precision and continuity, and the micromotor system is non-invasive, safe and controllable, which has great potential for application in nerve regeneration therapy. Dong [307] et al. encapsulated peroxythiocarbamate (peroxyTCM) with methoxy poly (ethylene glycol)-poly (l-methionine) nanoparticles (PTCM@PMet NPs). They encapsulated them into the precursor solution of a thermosensitive hydrogel (methoxypoly (ethylene glycol)-poly(l-methionine)-poly(phenylalanine) (mPEG-PA-PP)) and constructed a ROS-triggered on-demand H_2_S-releasing "multi-messenger" system (Fig. 8C). With the depolymerization of heat-sensitive hydrogel, the accumulation of ROS in the lesion area destroyed PTCM@PMet NPs and further triggered the release of H_2_S, which reduced the apoptosis of rat RSCs and induced the polarization of macrophages toward the M2 phenotype, and such a multifunctional combinatorial strategy effectively regulated the tissue regeneration microenvironment and greatly promoted nerve regeneration. It is worth mentioned that multiple response signals are also included in the synergistic effect of multiple response stimuli that can be generated by the same SRM in a system, such as photothermal therapy and photodynamic therapy. Jing [308] et al. doped magnesium-modified BP nanosheets (BP@Mg) into gelatin methacryloyl (GelMA), and developed a light-sensitive conductive hydrogel, GelMA-BP@Mg (GBM), in which BP possessed high electrical conductivity and positive effects on nerve repair and bone regeneration, and Mg promoted osteogenic differentiation of BMSC and production of calcitonin gene-related peptide (CGRP) in localized nerves. By treating BP with near-infrared irradiation, which converted light energy into heat energy based on photothermal properties and kills pathogenic microorganisms, and generated ROS based on photodynamic properties to destroy bacterial structure and increased bacterial thermal susceptibility, the hydrogel possessed strong antimicrobial activity and improved the inflammatory microenvironment. Conducting nanosheets and bioactive ions released from BP@Mg synergistically improved the migration and secretion of chervonia cells, and promoted neuronal synaptic growth.

Despite a growing number of studies reporting a variety of design options for multiple response systems, there are still limitations to the clinical application of multiple SRM in the field of neural regeneration. While SRM is able to respond to certain pathological features, these features may not be exclusively present at the site of injury, which leads to a reduction in the sensitivity and accuracy of the material in a highly complex and dynamic environment in vivo. Then, polymeric materials with multiple response mechanisms usually require complex synthesis procedures that are difficult to translate clinically and produce on a large scale. Therefore, if different response functions of the same SRM can be developed and achieved, the intelligent response system can be simplified and programmed as far as possible, and its application to neurotherapy may have important potential applications.

## 6 AI for nerve system

Artificial Intelligence (AI) refers to the simulation of human intelligence processes by machines, particularly computer systems. These processes include learning (acquiring and applying knowledge), reasoning (drawing logical conclusions), and self-correction. AI systems are typically designed to perform tasks that would otherwise require human cognitive functions, such as decision-making, problem-solving, natural language understanding, perception, and adaptive behavior. Being inspired by the organization of the nervous system, almost all modern AI systems rely on artificial neural networks (ANNs) [309]. ANNs model neural computation uses simplified units that loosely model the integration and activation properties of real neurons [310]. From past studies, scientists generally prefer to be able to explain as much neural activity as possible through a simple model, such as Hubel and Wiesel's 1958 experiments on the cat visual cortex [311],which firstly observed that neurons in the visual primary cortex were sensitive to moving edge stimuli, and subsequently launched a series of modeling explorations of stimulus responses. However, neural networks contain the involvement of countless individual neurons, and with that, simple models have become difficult to explain deeper neural networks. The inclusion of deep learning models for AI is therefore inevitable.

In the past few years, the development of AI has become increasingly sophisticated in the field of regenerative medicine, for example, AI algorithms can be able to detect diseases at an expert level from medical images by analyzing complex images, detecting small abnormalities and extracting quantitative features [312]. Goubran's team [313] constructed a mapping technique (ACE) using a 3D deep learning segmentation model and advanced cluster statistical algorithms, and applied this integrated ACE in two different neurobiological contexts to achieve unbiased mapping of local neuronal activity and connectivity, revealing the ability of localized or laminar neuronal activity throughout the brain. In addition, AI can integrate machine learning (ML) and histology techniques. Alganmi [314] et al. combined ML and histology-related keywords for structured searches and found that ML algorithms were increasingly used for biomarker discovery and diagnosis of rare neurological disorders (RNDs), with a major focus on genomics and radiomics, which enhanced the diagnostic process for rare neurological disorders. In addition, AI-based virtual screening and predictive modeling can also be used to analyze and predict interactions between drugs and their targets to find possible therapeutic molecules [315]. Zanini [316] et al. developed a morbidity prediction model by integrating measurements of about 3000 plasma proteins, and found that the prediction of 52 diseases including motor neuron disease, the predictive of this protein model performance were better than the model combining clinical test data and basic information, and also predicted specific expression in proteins, which will be beneficial for the prediction of disease protein factors. Overall, this has the potential to fundamentally change the paradigm of personalized medicine for neurological disorders by using AI to reveal hidden patterns and connections in complex data.

AI can not only help researchers better parse neurological diseases, but can also be used to assist in the design of SRM. SRM is capable of changing their chemical and physical properties in response to external stimuli in adaptive, interactive and self-regulating modes, however, there are still many difficulties with scalability, reproducibility and robustness [317,318],and combining with AI technology may overcome these challenges. For example, based on hydrogel-based biomaterials, AI can analyze thousands of combinatorial hydrogels with different chemical structures, and accelerate the design of novel hydrogels by calculating a large library of molecules with different chemical structures and millions of parameters that effectively link the molecular backbone to the properties of stimulus response materials [319]. SRM is usually structurally more complex and less stable, which may affect their drug release kinetics, and by using training data based on the type, size, and structure of the material and drug, material-drug interactions, type of external stimulus, and other factors, the drug release behavior of SRM can be accurately predicted using an AI-based approach [320]. Balan [321] et al. successfully synthesized novel poly (NIPAAm-co-VSA)/alginate IPN hydrogels with dual pH and temperature response by free radical polymerization, and simulated the complex release behavior of the loaded adriamycin (DOX) by ANN technique, and it was in good agreement with the experimental results, which indicated that there is a large potential for the development of AI in the application of drug controlled release systems.

The rise of deep learning has prompted us to revisit ANNs. neuroscience will inevitably require both bottom-up guided and top-down theoretical work. Given the ability of modern ML to address both the AI set and numerous brain sets, it would be fruitful to use ML insights to guide a top-down framework for systems neuroscience research [322]. “NeuroAI” is the intersection of neuroscience and AI, and has been interpreted to mean that a better understanding of neural computation will reveal the fundamental components of intelligence and catalyze the next revolution in AI. Therefore, AI might also drive the next revolution in neuroscience with SRM.

## 7 Perspectives and conclusion

**Fig. 9:**
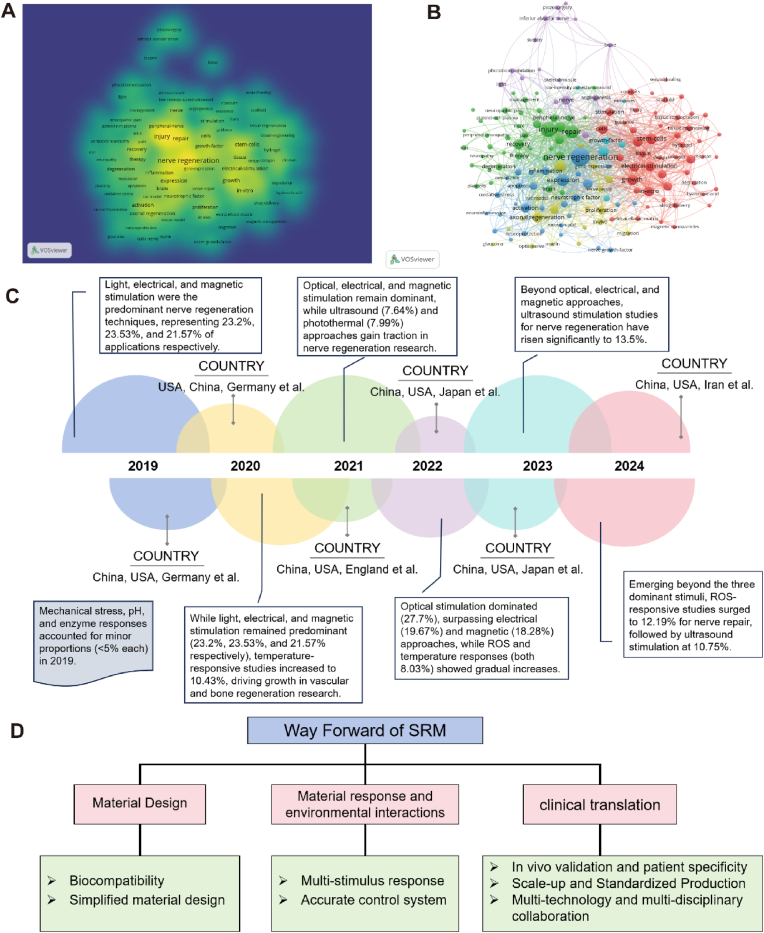
Visual analysis of literature search on SRM in the field of neuroregeneration. **(A)** Density visualization analysis. **(B)** Network visualization analysis. **(C)** Timeline plot of SRM applied to neural regeneration in recent years. **(D)** Future Research Directions for SRM.

In order to obtain a more comprehensive understanding of the research progress of SRM in the field of nerve regeneration, more than 1500 documents related to SRM and nerve regeneration in the past 5 years were retrieved from Web of Science, and the visualization and analysis diagrams were drawn using WORDART and VOS-viewer. From the density visualization and analysis diagram (Fig. 9A), it can be observed that the stimulus response technology in the field of neural regeneration occupies a not-so-small proportion, and the network visualization and analysis diagram (Fig. 9B) show that electrical stimulation, as the backbone of the intelligent response system, has gained rapid momentum in its research and application. In addition, light, magnetic nanoparticles and ultrasound are also not to be underestimated, and have certain R&D potential in the field of nerve regeneration. Then statistics on the main research trends of SRM applied to nerve regeneration and the countries where the articles are published each year (Fig. 9C), optical, electrical and magnetic stimulation has always been a popular research in nerve regeneration technology, accounting for no less than 20 %, and with the further deepening of the research and technological development, the direction of ultrasound and ROS response signals is gradually increasing, and scientists are not only focusing on the nerve regeneration, but also on the complex physiological environment of vascular regeneration, bone regeneration, immune response and other multi-layered and multi-directional research, bringing a new field of research.

Compared to traditional nerve regeneration methods/techniques: autologous/allogeneic nerve grafts and static nerve conduits are all limited by the length and diameter of the injury, immunosuppression, hemodialysis reconstruction needs, and functional matching [323,324]. SRM, can overcome the above challenges by performing structural or functional tailoring or modification to reduce the associated side effects and promote functional reconstruction to match the regenerative needs of the injured nerve. In this review, the categories of SRM and their response mechanisms are summarized, highlighting the responsive materials commonly used in the field of neuroregeneration. To further specify the application areas of SRM, case studies of various types of SRM in the field of neural regeneration are presented according to different design strategies. Finally, the limitations of each type of SRM are discussed separately, and the focuses and improvements of SRM applied to nerve regeneration are pointed out (Fig. 9D). The specific summary is shown in Table 1. Despite the growing number of relevant scientific publications in recent years, few technologies have successfully entered clinical trials in neuroregeneration. Therefore, unfortunately the current review has not been able to summarize the application of SRM for neural regeneration in the clinic.

**Table 1:** Summary of SRM for neural regeneration applications.

Signal Source	Response Type	Key components	Design strategy	Functional application	Ref
**Exogenous signals**	Temperature	PNIPAM, curcumin, β-cyclodextrin	A modified nanogel delivery system was used to graft PNIPAM and β -cyclodextrin onto hyaluronic acid (PNCDHA) to encapsulate curcumin.	According to its thermosensitive property, curcumin was released from cur-loaded nanogel at 37 °C for 12 h. And, curcumin-loaded nanogel exhibited a degree of biocompatibility.	[46]
		HP, NGF	lyophilized HP powder was mixed with NGF solution (phosphate buffered saline, pH = 7.6) at 4 °C under gentle stirring. The mixture was kept in a refrigerator at 4 °C overnight until a clear solution was formed.	NGF-HP hydrogel combination treatment significantly increased the efficiency of cellular uptake of NGF (P < 0.05) without significant cytotoxicity. Improved neuronal function and histomorphology in SCI rats. Significantly inhibited the formation of neuroglial scars in extremely extruded SCI rats.	[64]
		IKVAV, PEGDA, BISAM	The hydrogel was prepared by copolymerization of N-propan-2-ylprop-2-enamide (NIPAM) and AC-PEG-IKVAV copolymers via reversible addition-fracture chain transfer (RAFT) polymerization, using polyethylene glycol (PEGDA) and N, N′-Methylenebis-acrylamide (BISAM) as cross-linking agents.	The prepared hydrogel scaffold demonstrates a series of excellent properties such as rapid (de)swelling performance, good biocompatibility, regular three-dimensional porous structure, and in particular good biological activity, which can guide cell fate and mediate neuron's differentiation.	[53]
	Magnetism	MNPs, CS	Introducing magnetic nanoparticles (MNPs) with different contents into carboxymethyl chitosan (CS) hydrogels through a crosslinking approach, which could provide magnetically active microenvironment.	The intrinsically-present magnetic cues in hydrogel contributed to the activation of intracellular RAS-dependent signal cascades, thus facilitating neuronal differentiation.	[75]
		PCL, MLT, Fe_3_O_4_-MNPs	A multilayered composite NGC (ML-NGC) loaded with MLT and Fe_3_O_4_-MNPs was fabricated by electrospinning.	The multilayered composite NGC loaded with MLT and Fe3O4-MNPs is designed for sequential and sustainable drug release, creating an appropriate microenvironment for nerve regeneration. The composite scaffold shows sufficient mechanical strength and biocompatibility in vitro, and evidently promotes morphological, functional, and electrophysiological recovery of regenerated sciatic nerves in vivo.	[70]
		SPIONs, citric acid, AdMSC	AdMSC were loaded with citric acid coated SPIONs, systemically transplanted and magnetically recruited to the injured sciatic nerve.	With magnetic targeting, there is a significant increase in AdMSC reaching the injured nerve with beneficial effects that exceed the regenerative properties of stand-alone cell therapy. and supported intact myelinated axons. Improved nerve conduction showed restoration of function.	[72]
	Electronic	PLGA, CNT, PCL	A series of multichannel nerve conduit was made using longitudinally-aligned laminin-coated poly (lactic-co-glycolic acid) (PLGA)/carbon nanotubes (CNT) nanofibers (NF, mean diameter: 455 ± 362 nm) in the lumen and randomly-oriented PCL NF (mean diameter: 340 ± 200 nm) on the outer surface.	The results of motor and sensory tests in addition to histopathological examination of the regenerated nerves demonstrated the formation of nerve fibers in laminin-coated PLGA/CNT NF-embedded PCL conduits.	[106]
		PCL, collagen, MWCNTs	The study took the effort of modulating the pattern (arrangement) of reinforced phase, namely multiwalled carbon nanotubes (MWCNTs), in a biodegradable scaffold made of PCL-collagen mixture, by applying an external electric field during curing.	These casted carbon nanostructure reinforced scaffolds facilitated neural differentiation in vitro. And the scaffold with aligned MWCNTs promotes the bidirectional growth of the neuronal cells in a direction parallel to the direction of the MWCNTs alignment.	[108]
		PEDOT NPs, SF	Novel poly(3,4-ethylenedioxythiophene) PEDOT nanoparticles (PEDOT NPs) were synthetized via the mini-emulsion method and were combined with silk fibroin (SF) to create conduits for PNI repair.	The conduits demonstrated to be a good surface for SCs proliferation, and moreover, they did not allow BJ fibroblasts infiltration, avoiding scar tissue formation in the lumen.	[119]
	Mechanical stimuli	PDMS	Prepared PDMS substrates with different base to curing agent ratios at room temperature.	Higher cell densities for both DRG neurons and glial cells grown on semi-rigid polydimethylsiloxane substrates (PDMS ratio of base to curing agent of 35:1) than found on more rigid (15:1) or more flexible (50:1) substrates, indicating a localized bimodal response within a very small difference of elasticity on PDMS.	[177]
		SF, Alg-BP, Mg^2+^	Owing to highly dynamic interactions between bisphosphonate-modified alginate (Alg-BP) and Mg^2+^, the obtained hydrogels exhibited remarkable dynamic properties, and the thiol-ene click reaction between glutathione (GSH) and methacryloyl (MA) groups on SF created the interpenetrating polymer network (IPN).	The adaptable hydrogels facilitated favorable cell-material interactions, including SCs migration and neurite outgrowth, contributing to muscle recovery from atrophy and nerve functional recovery.	[185]
	Ultrasound	PAM, graphene, PLA, PDMS	The composite hydrogel was prepared by dispersing graphene into PAM, and subsequently, the composite hydrogel was encapsulated with a PDMS template through a 3D printing system, and then Au wires were embedded in the PAM/graphene hydrogel to serve as the collection electrodes.	The subcutaneously implanted HENG can be used directly as a wireless neurostimulator. Ultrasound-responsive HENG has shown the ability to inhibit pro-inflammatory cytokines by radio-stimulation of the vagus nerve.	[215]
		PCL, PVDF	Through electrospinning, linearly aligned piezoelectric nanotracks composed of PCL and polyvinylidene fluoride (PVDF) were fabricated.	Sono-electro-mechanical promotion of pro-regeneration SCs functions and neuronal growth in vitro and restores complex motor function and axonal maturation in rats.	[216]
	Light	PDA, collagen	The use of biocompatible and biodegradable polydopamine nanoparticles and a novel highly porous biofoam as photothermal agents to stimulate excitable cells such as neurons and cardiomyocytes with NIR light in a non-disruptive manner.	The modulation of the activity (i. e. spike rate of the neurons and beating rate of cardiomyocytes) of excitable cells could be finely controlled by varying the excitation power density and irradiation duration.	[248]
		MEO_2_MA, OEGMA	The highly temperature-sensitive semi-interpenetrating optical hydrogel fiber (TSOHF) was fabricated by using the integrated dynamic wet-spinning technique. The semi-interpenetrated structure of core hydrogel fiber ensured that the 2-(2-methoxyethoxy) ethyl methacrylate (MEO_2_MA)/oligo (ethylene glycol) methacrylate (OEGMA) random copolymer endowed the fiber with temperature sensitivity while maintain its' excellent light propagation property.	TSOHF exhibits a structural tunable diameter, clear core/sheath structure, tunable temperature-sensitivity, excellent light propagation property (0.35 dB cm^−1^, 650 nm laser light), and good biocompatibility (including tissue-like Young's modulus, stable dimensional stability, and low cytotoxicity).	[251]
		PNIPAM, MWCNT	Surface modification technology and in-situ free radical polymerization were used to prepare a photothermal responsive self-rolling PNIPAM hydrogel containing dopamine hydrochloride modified MWCNTs.	The hydrogel here possesses a promoting effect on nerve cells growth, and is expected to further improve the capability of nerve grafts on repairing PNI.	[50]
**Endogenous signals**	pH	IL-4 pDNA, liposomes, microsol	The aldehyde-modified cationic liposomes loading IL-4 plasmid DNA (pDNA) were grafted onto the surface of amino-modified oriented microsol electrospun fiber scaffolds through Schiff base bond that could break under acidic environment.	This strategy provided a delivery system through microenvironment-responsive immunological regulation effect so as to break through the current dilemma from the contradiction between immune response and nerve regeneration.	[280]
	Enzyme	MNPs, NSC, Alginate	Alginate hydrogel micromotor with a capillary microfluidic chip to encapsulate NSCs and MNPs.	This miniaturized machine supported the growth of neural stem cells, and precisely transported them by external magnetic fields. Enzyme-responsive cell release in the targeted regions was allowed, facilitating reconstruction of neural connection.	[286]
	Redox	PPS120, Cur, TM	Hydrophobic poly (propylene sulfide)120 (PPS120) was synthesized, with a ROS quencher and H_2_O_2_-responsive abilities, to embed Cur. Matrix metalloproteinase (MMP)-responsive triglycerol monostearate (TM) was used to cover the PPS120 to form a TM/PC hydrogel.	TM/PC hydrogel effectively protected against brain injury by decreasing the ROS level and enhancing nerve functional recovery.	[293]
**Multiple signals**	Thermo/redox/pH	NIPAM, AA, BAC, MBA	Redox-sensitive/degradable NGs (PNA-BAC) and nondegradable NGs (PNA-MBA) were prepared through in situ polymerization of N-isopropylacrylamide (NIPAM) and acrylic acid (AA) in the presence of sodium dodecyl sulfate (SDS) as a surfactant, using N, N′-bis(acryloyl)cystamine (BAC) as a biodegradable crosslinker or N, N′-methylene bisacrylamide (MBA) as a nondegradable crosslinker, respectively.	The thermo/redox/pH multi-sensitive NGs can quickly be taken up by CAL-72 cells (an osteosarcoma cell line), resulting in a high doxorubicin (DOX) intracellular accumulation and an improved cytotoxicity when compared with free DOX and DOX-loaded nondegradable PNA-MBA NGs. The developed NGs can be possibly used as an effective platform for the delivery of cationic therapeutic agents for biomedical applications.	[45]
	Temperature/pH/ROS	peroxyTCM, mPEG-PMet, mPEG-PA-PP	This H_2_S delivery system consists of an H_2_S donor (peroxyTCM) encapsulated in a ROS-responsive polymer (mPEG-PMet) and loaded into a temperature-sensitive poly (amino acid) hydrogel (mPEG-PA-PP).	This multifunctional strategy successfully inhibited inflammation and oxidative stress, protected neuronal cells, promoted angiogenesis, and restored normal mitochondrial function, which greatly facilitated the regeneration of PNI.	[307]

PNIPAM, Poly(N-isopropylacrylamide); HP, Heparin-poloxamer; NGF, Nerve growth factor; IKVAV, Ile-Lys-Val-Ala-Val; NIPAM, N-propan-2-ylprop-2-enamide; PEGDA, Polyethylene glycol; RAFT, Reversible addition-fracture chain transfer; BISAM, N, N′-Methylenebis-acrylamide; MNPs, Magnetic nanoparticles; CS, Chitosan; PCL, Polycaprolactone; NGC, Nerve guidance conduit; MLT, Melatonin; Fe_3_O_4_-MNPs, Fe_3_O_4_ magnetic nanoparticles; AdMSC, Adipose-derived mesenchymal stem cells; SPIONs, Superparamagnetic iron oxide nanoparticles; PLGA, Poly (lactic-co-glycolic acid); CNT, Carbon nanotubes; NF, Nanofibers; MWCNTs, Multiwalled carbon nanotubes; PEDOT, Poly(3,4-ethylenedioxythiophene); SF, Silk fibroin; SCs, Schwann cells; PDMS, Polydimethylsiloxane; Alg-BP, Bisphosphonate-modified alginate; GSH, Glutathione; MA, Methacryloyl; IPN, interpenetrating polymer network; PAM, Polyacrylamide; PVDF, Polyvinylidene fluoride; PDA, Polydopamine; NIR, Near Infrared; TSOHF, Temperature-sensitive semi-interpenetrating optical hydrogel fiber; MEO_2_MA, 2-(2-methoxyethoxy) ethyl methacrylate; OEGMA, Oligo (ethylene glycol) methacrylate; pDNA, plasmid DNA; PPS120, Poly (propylene sulfide)120; MMP, Matrix metalloproteinase; TM, Triglycerol monostearate; ROS, Reactive oxygen species; Cur, curcumin; AA, Acrylic acid; SDS, Sodium dodecyl sulfate; BAC, Bis(acryloyl)cystamine; MBA, Methylene bisacrylamide; DOX, Doxorubicin.

From materials design perspective, the ultimate goal of SRM is to achieve and maintain reliable and effective performance in highly complex in vivo environments while ensuring biosafety. Although SRM systems are usually constructed based on synthetic materials, an increasing number of natural polymers exhibit functional smart responses, such as piezoelectricity, electrical conductivity, and photothermal properties. Therefore, biocompatible materials or formulations, e.g., natural materials, are considered first in the design strategy. However, it should be noted that SRM applications are often limited by their structural and mechanistic complexity. The design of SRM for neural regeneration applications should be based on the principle of simplicity and translational feasibility, rather than focusing on complex structures or incorporating new approaches. Most "over-designed" SRM systems make clinical translation highly challenging [27].

Secondly, in terms of material response-environment interactions, since single stimulus responses are difficult to satisfy complex physiological microenvironments, the promotion of combining dual or multiple stimulus responses contributes to the dynamic recovery process of neural regeneration in order to make the ideal SRM meet all the critical quality attributes required for successful translation to meet actual clinical needs. However, for programmed SRM systems that can produce synergistic or sequential effects, the accuracy of the spatiotemporal control of each trigger also needs to be evaluated and, if necessary, also used in conjunction with simulation and modeling to refine the control system.

Thirdly, for clinical translation of SRM, their biocompatibility, long-term safety, and stimulus responsive efficiency must be further verified in vivo under complex physiological conditions while accounting for patient-specific variability. For example, the acidity and alkalinity of the microenvironment after injury may cause fluctuations in targeting efficiency for pH-responsive materials due to individual differences, and the dispersion and target aggregation ability of MNPs in physiological environments are also affected by individualized differences. In addition, bottlenecks for clinical translation include quality control for scale-up production, standardization of response parameters (e.g., external field stimulation intensity), and adaptability for regulatory approval. Currently, a few SRM (e.g., temperature-sensitive hydrogels) have entered clinical trials, but most of them remain in the laboratory stage. With the progress of the society and the innovation of technology, the systematic research and application of SRM will also require new characterization and computational methods, the optimization of material design through multidisciplinary collaboration, the real-time monitoring of material behaviors by combining with imaging technologies (e.g., MRI/fluorescence imaging), and the establishment of models that are closer to the human body in order to accelerate the clinical landing.

In summary, although the application of SRM is challenging and its future development cannot be separated from the deep integration of basic research, engineering and clinical medicine, it still provides a revolutionary tool for precision medicine and is expected to show a broad application potential in the field of tissue engineering and regenerative medicine.

## CRediT authorship contribution statement

**Hongxia Gao:** Writing – original draft, Methodology, Investigation, Formal analysis, Data curation. **Huoyun Shen:** Validation, Methodology, Investigation, Formal analysis, Data curation. **Xunrui Zhang:** Data curation, Investigation, Methodology, Validation. **Yaqiong Liu:** Software, Methodology, Investigation, Formal analysis. **Yuqing Shang:** Validation, Methodology, Investigation, Formal analysis. **Shaolan Sun:** Resources, Methodology, Investigation. **Wenchao Guan:** Validation, Software, Resources. **Xiaosong Gu:** Visualization, Validation, Software, Resources. **Yumin Yang:** Validation, Supervision, Resources, Project administration. **Guicai Li:** Writing – review & editing, Supervision, Project administration, Funding acquisition, Conceptualization.

## Data availability

The authors declare that the data supporting the findings of this study are available within the paper and its Supplementary Information. Raw data generated for this study are available from the corresponding author on reasonable request.

## Declaration of competing interest

The authors declare that they have no known competing financial interests or personal relationships that could have appeared to influence the work reported in this paper.
