# Supplementary material for: Revolutionizing neural regeneration with smart responsive materials: Current insights and future prospects
Source: Bioact Mater. 2025 Jun 13;52:393–421. doi: 10.1016/j.bioactmat.2025.06.003 (PMC12206011; doi:10.1016/j.bioactmat.2025.06.003)

***Supplementary file***

**Revolutionizing Neural Regeneration with Smart Responsive Materials: Current Insights and Future Prospects**

**Copyright of the figure 2 to figure 8**

**Fig2 A**


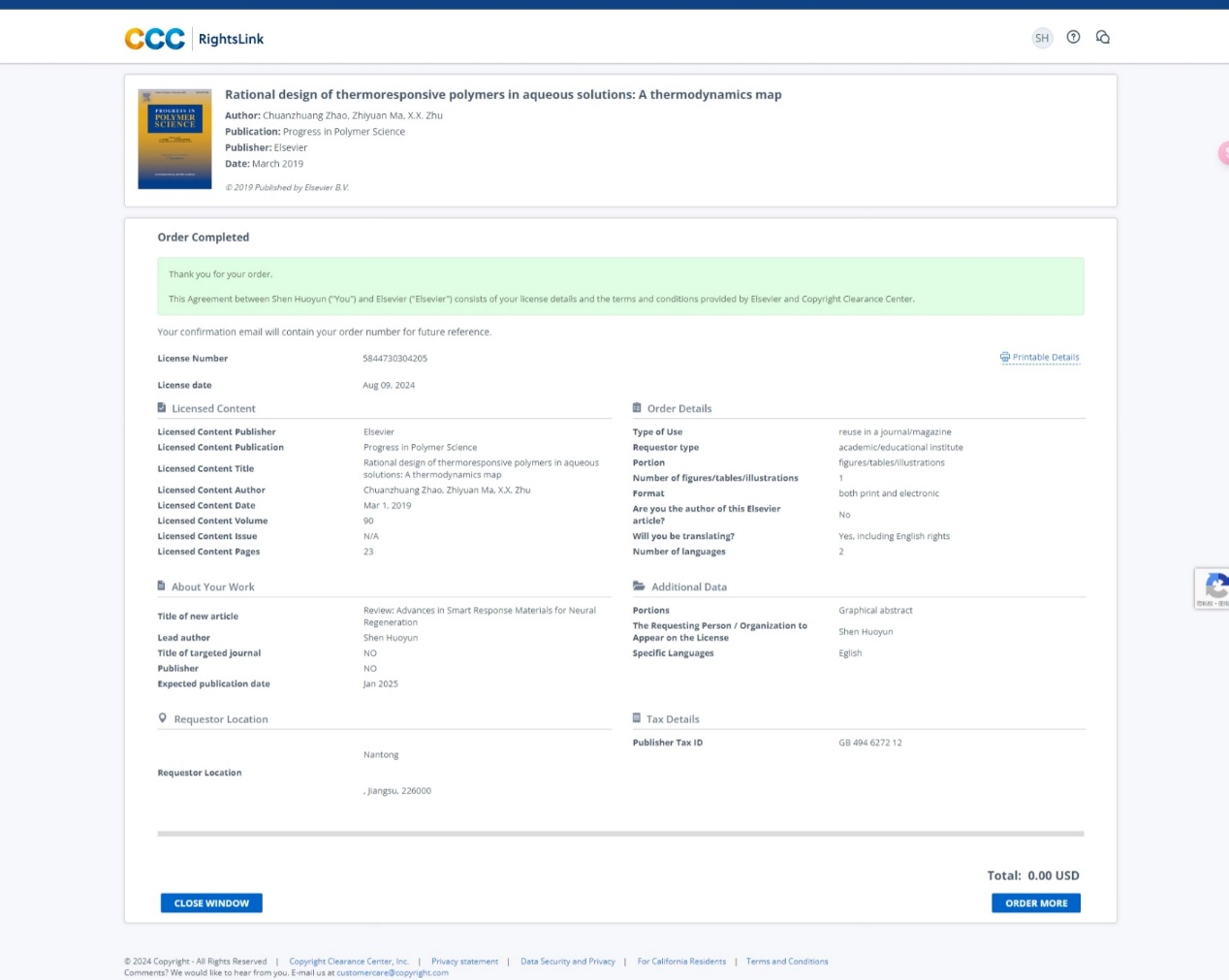


**Fig2 B**

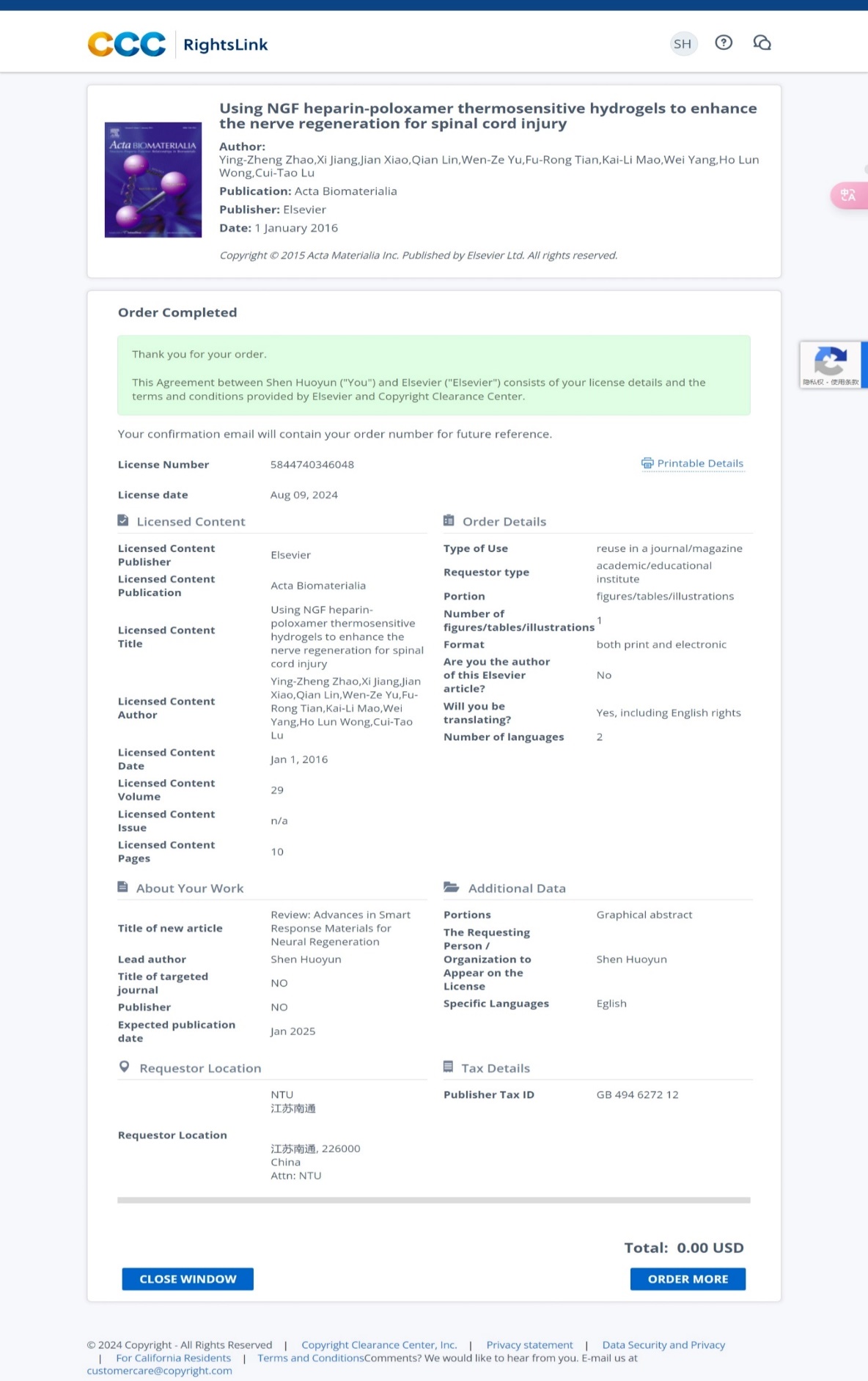


Fig2 C


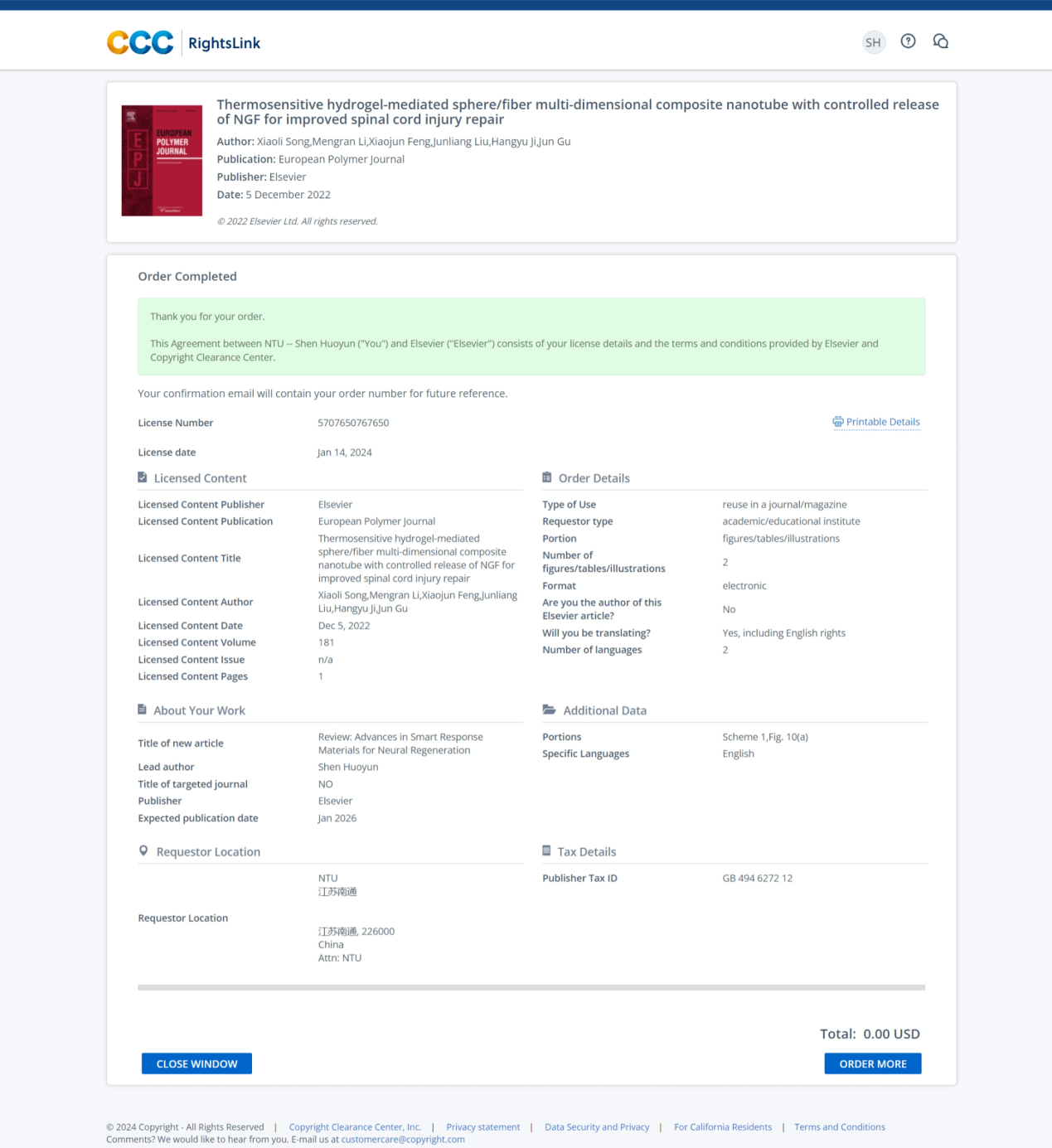


Fig2 D


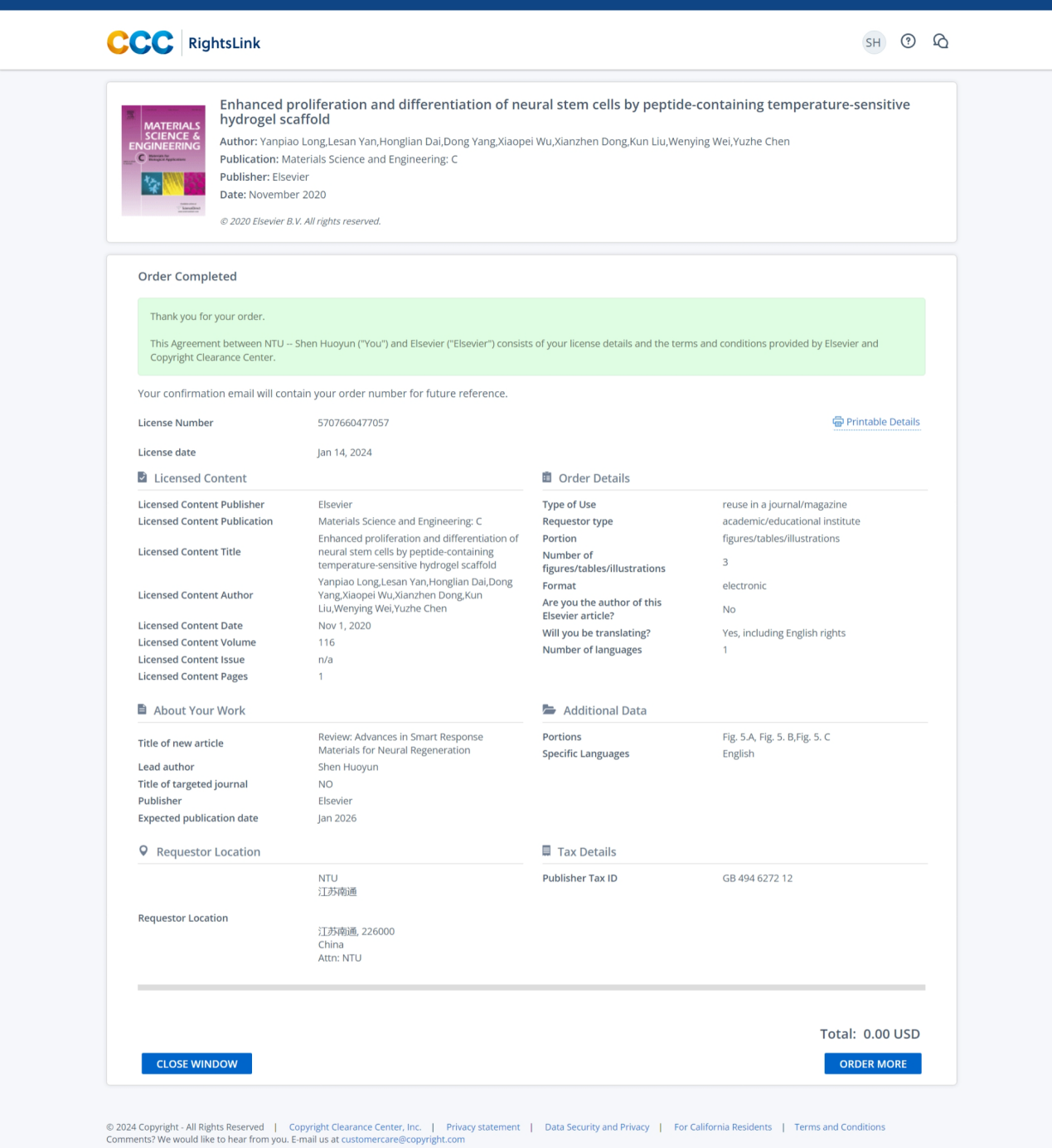


Fig3 A


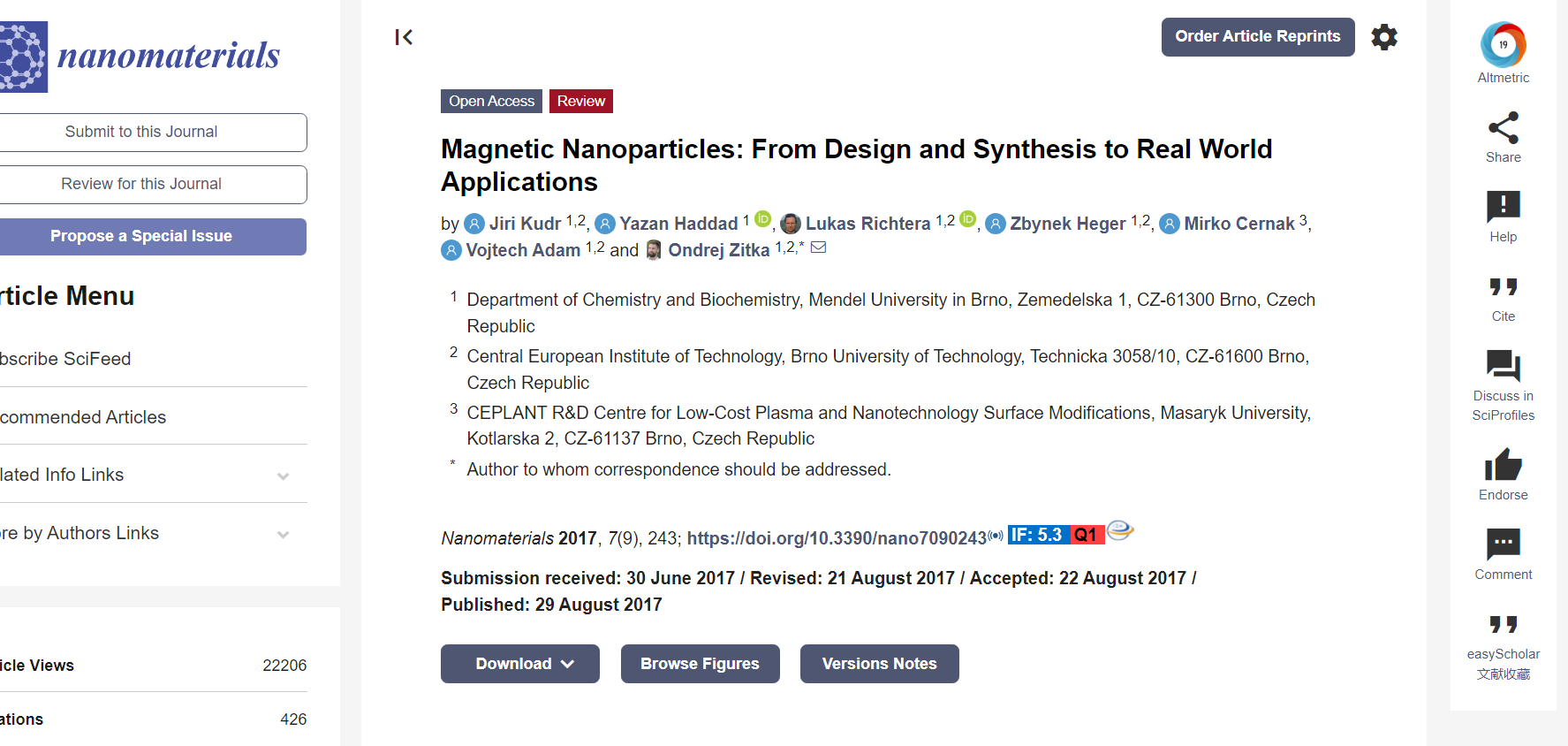


Fig3 B


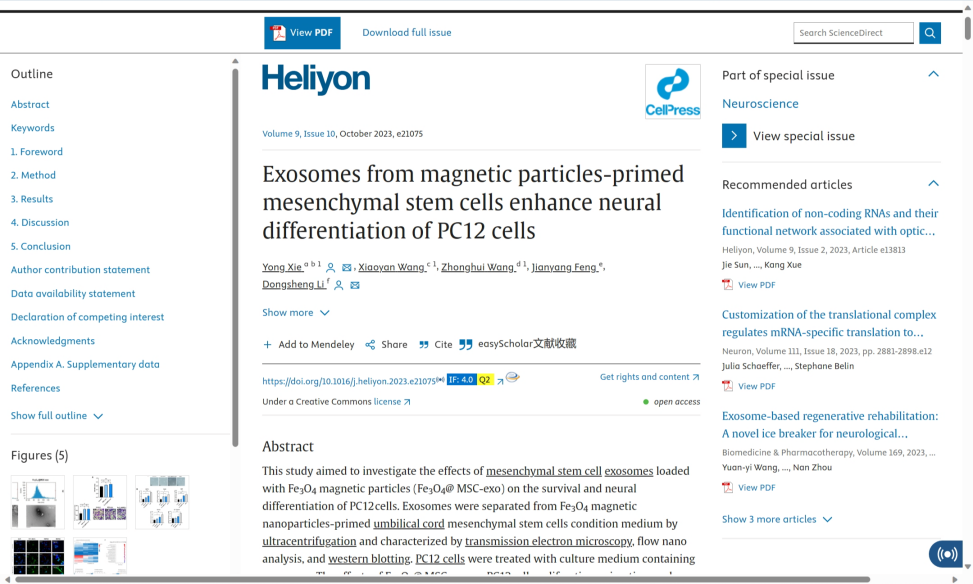


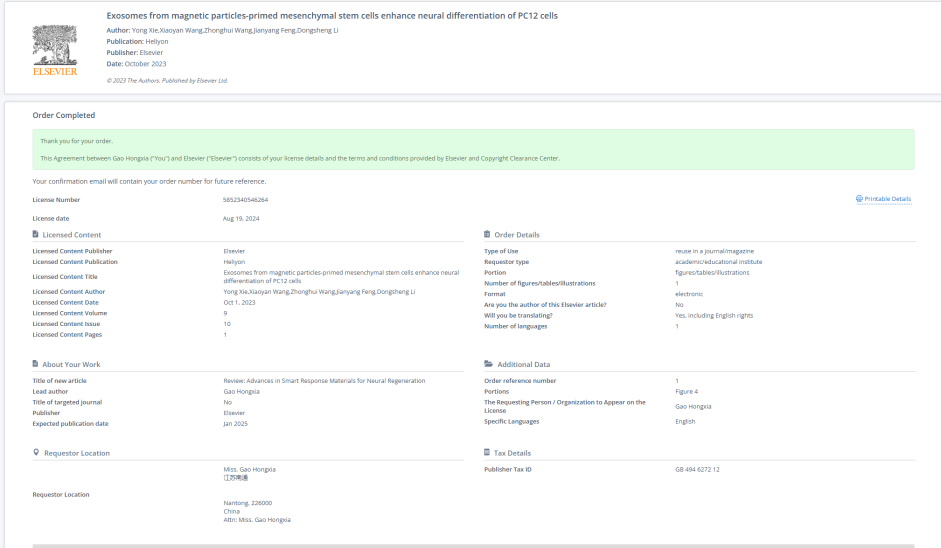


Fig3 C


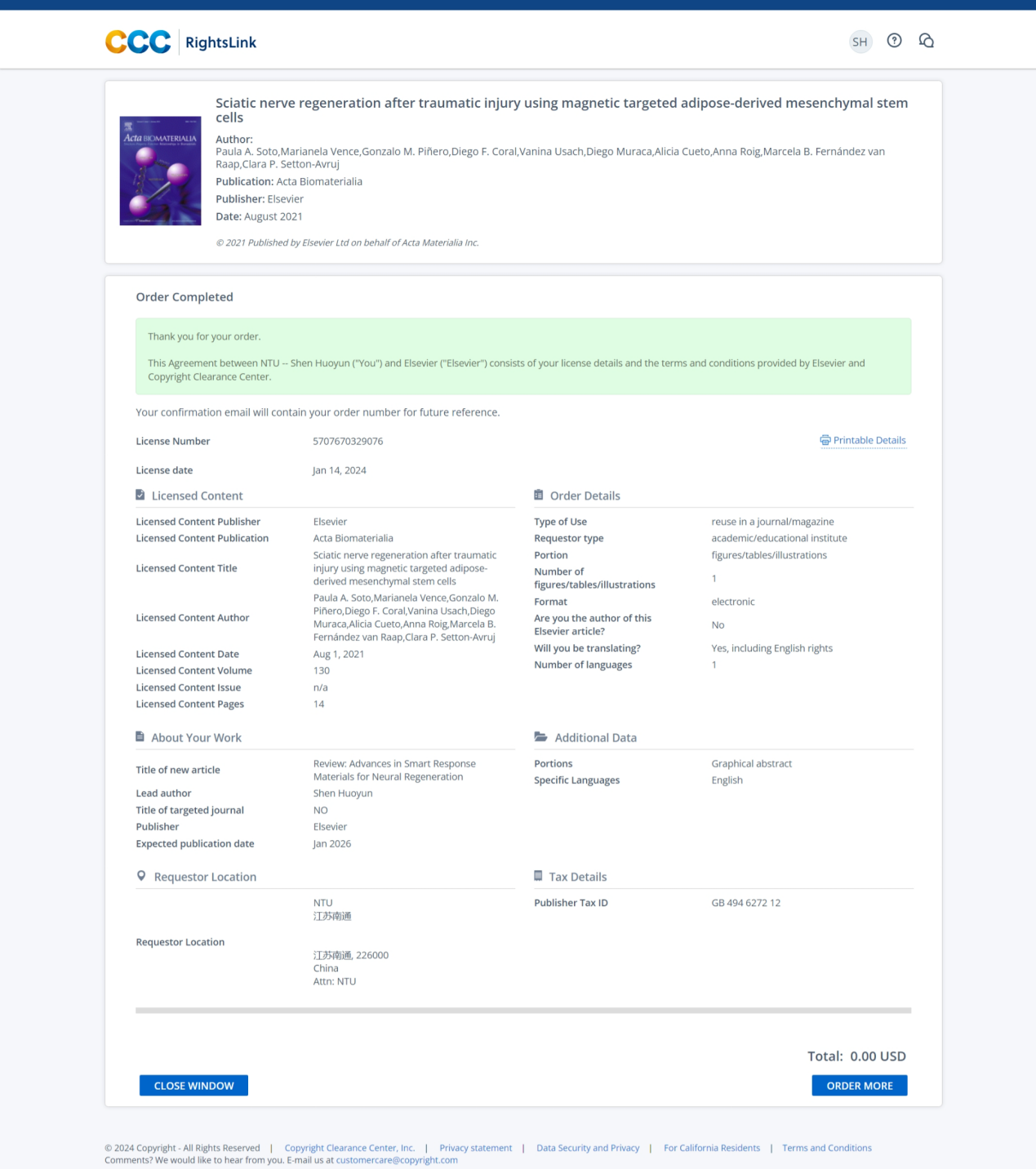


Fig3 D


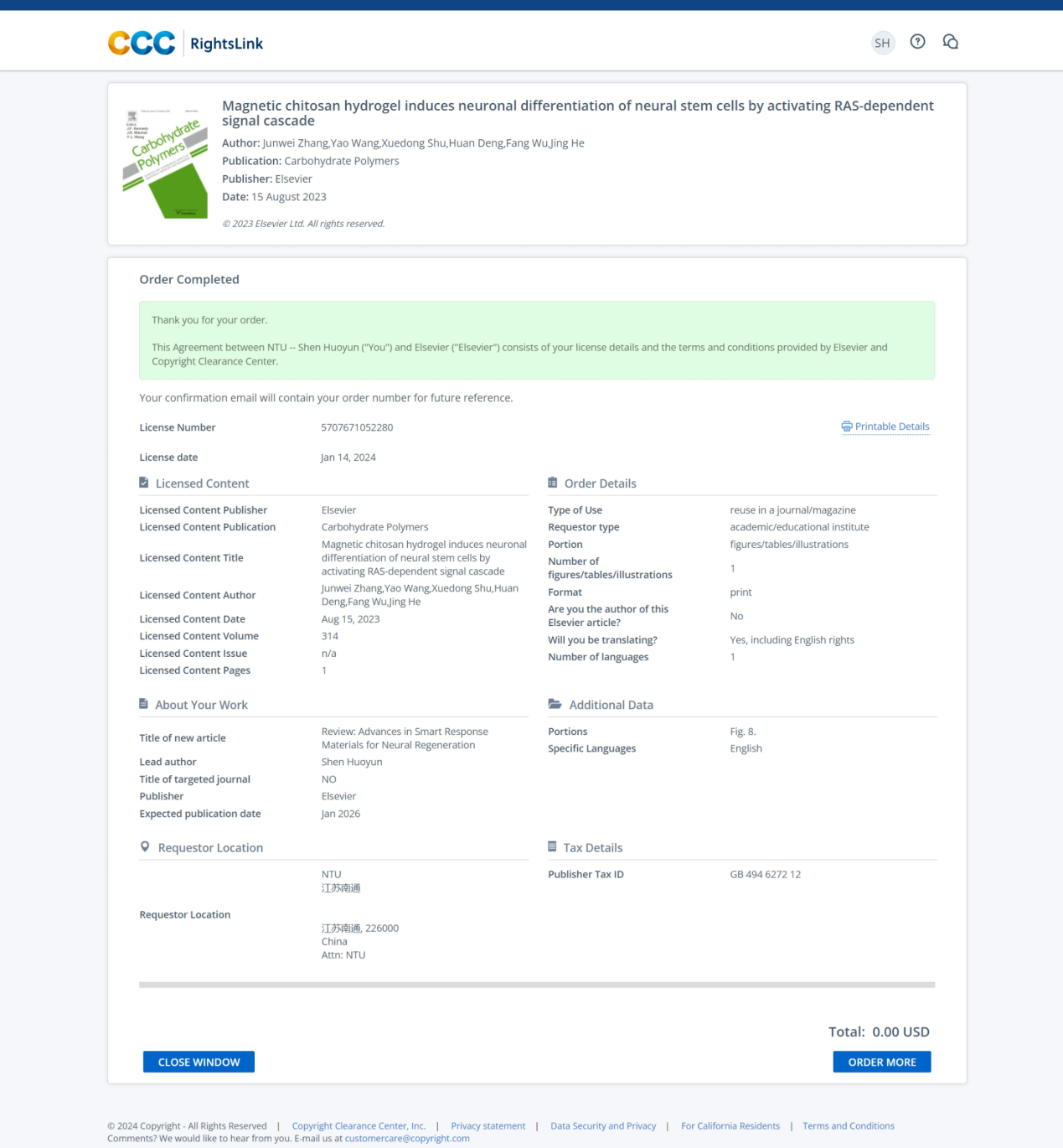


Fig3 E


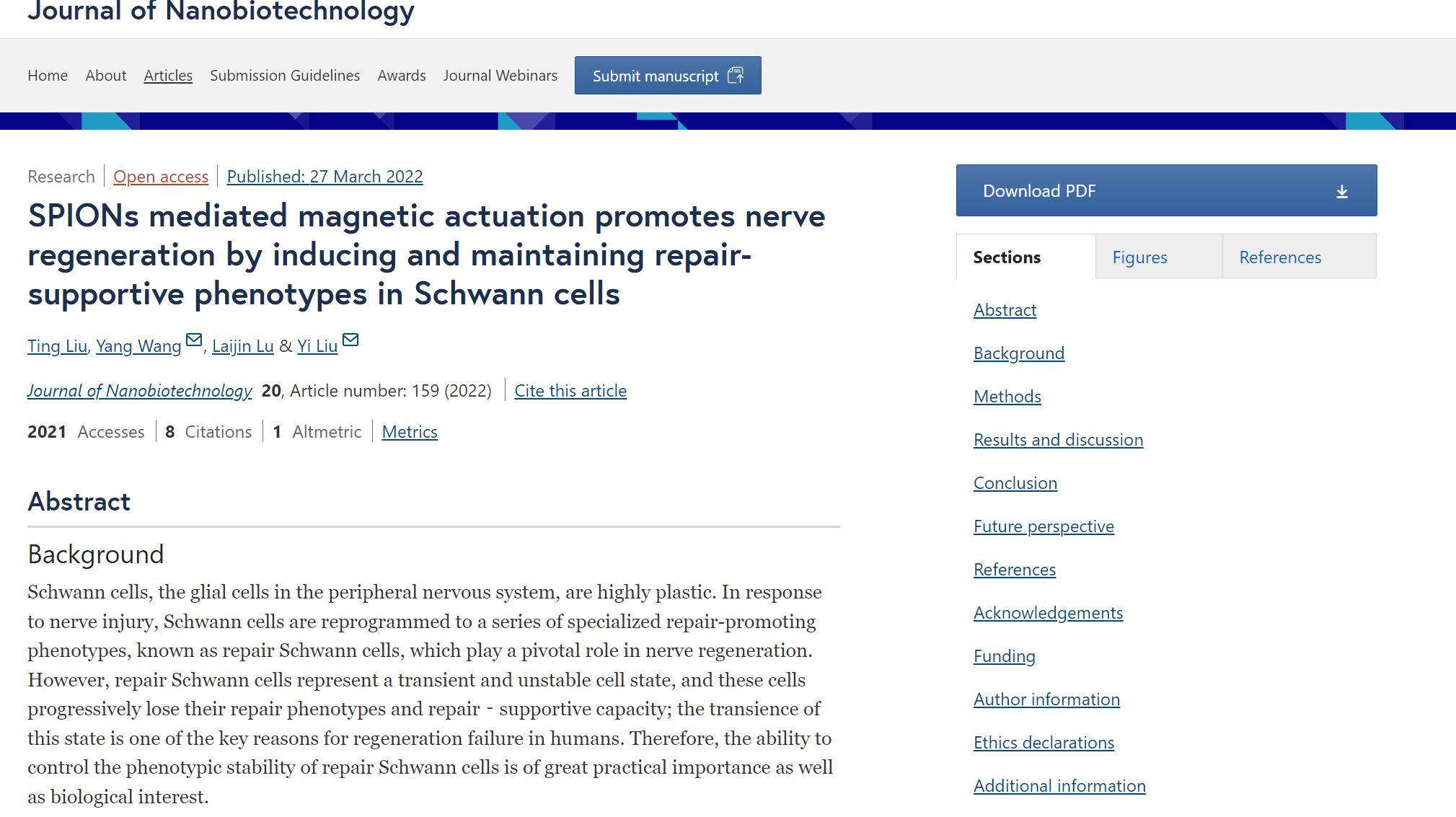


Fig4 B


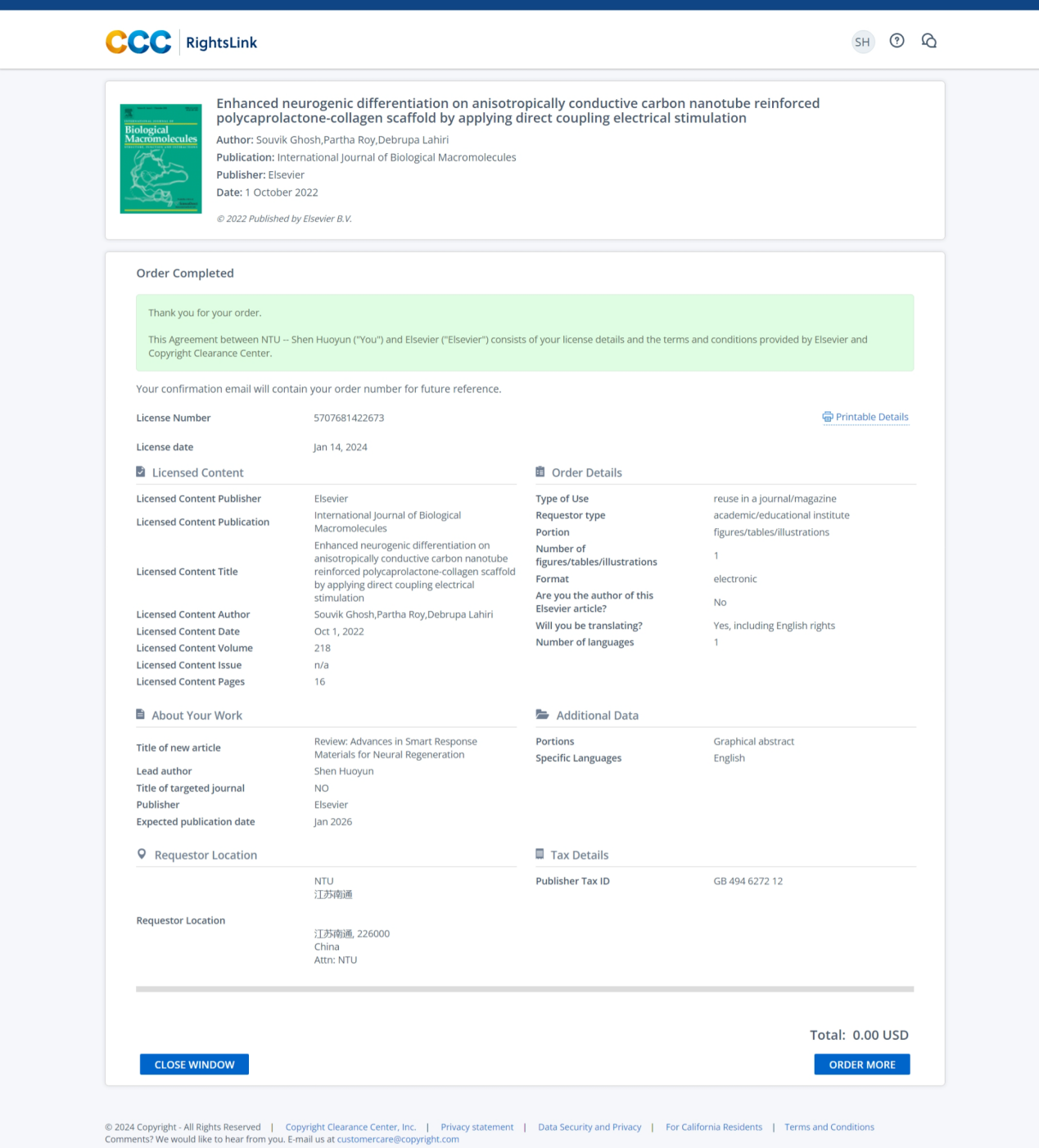


Fig4 C


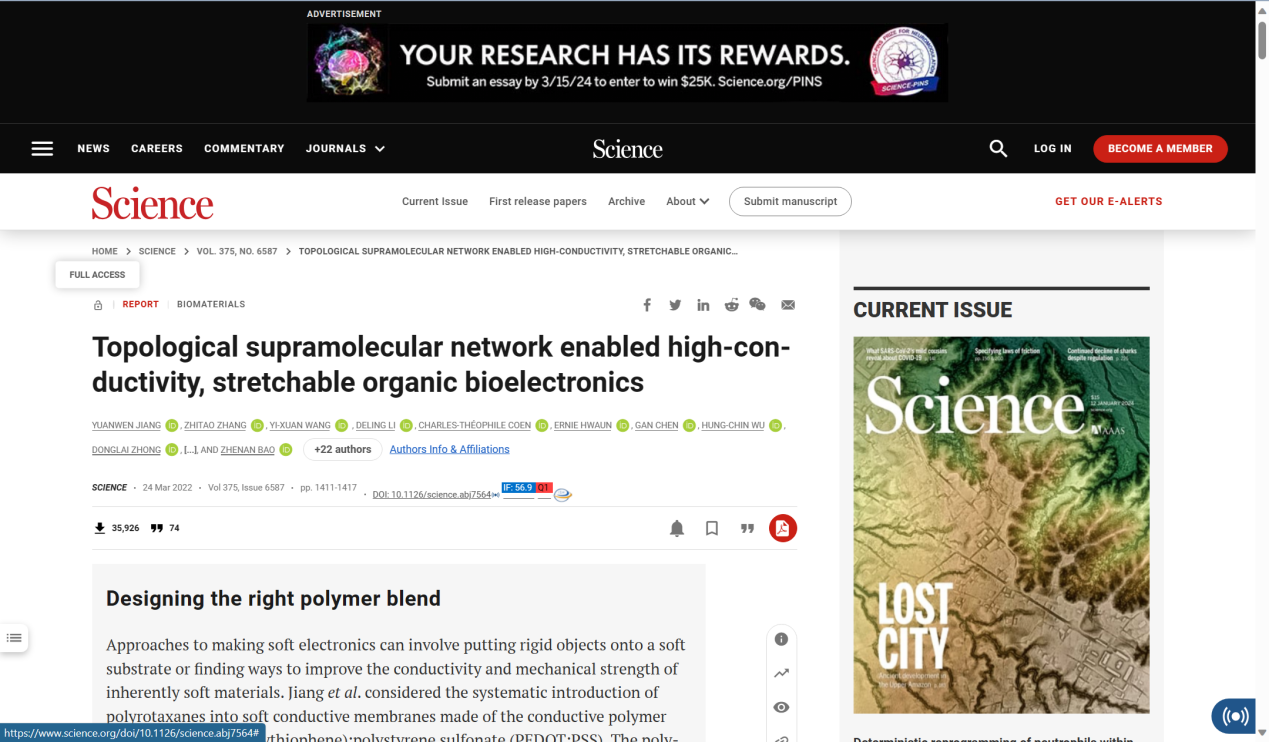


Fig5 A


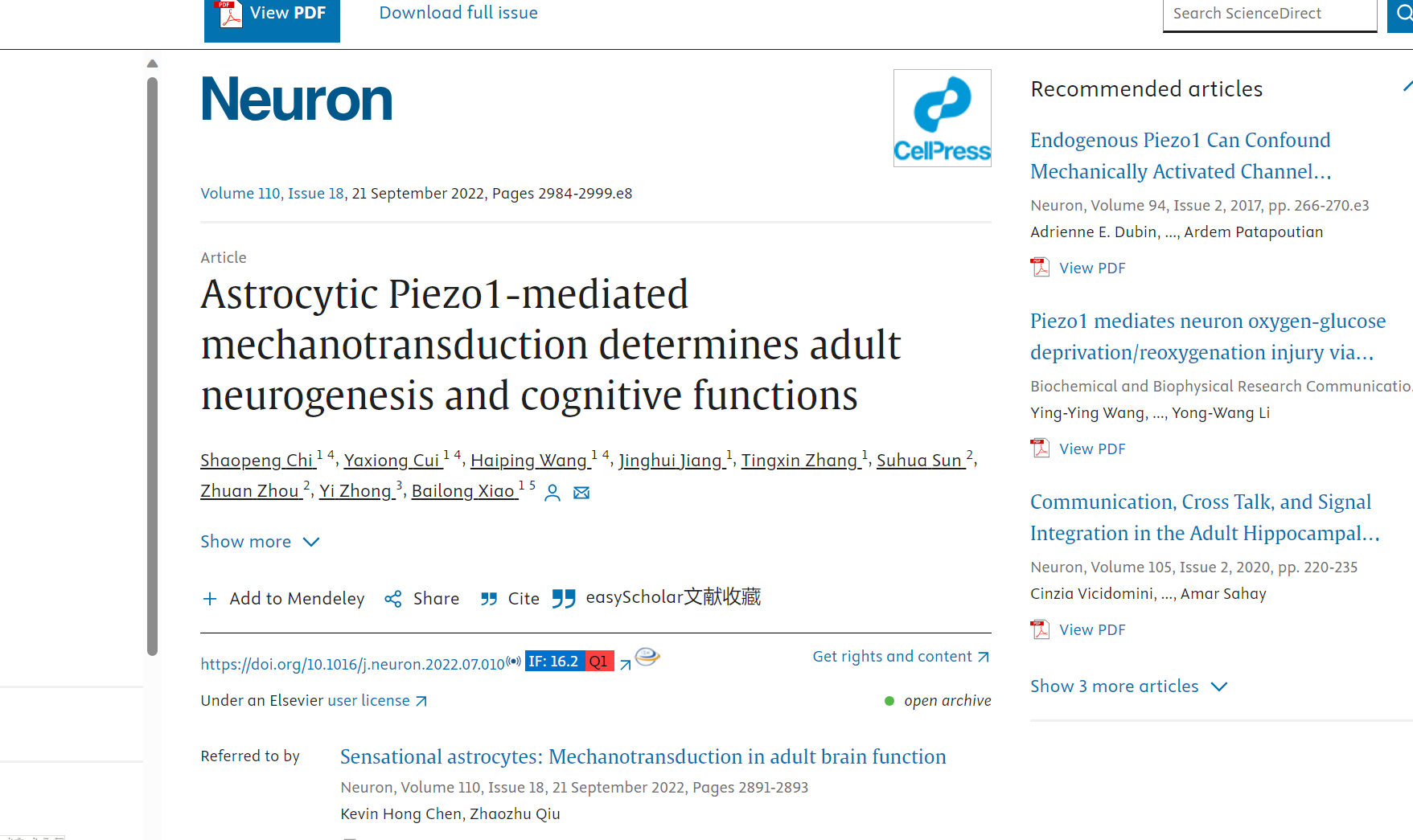


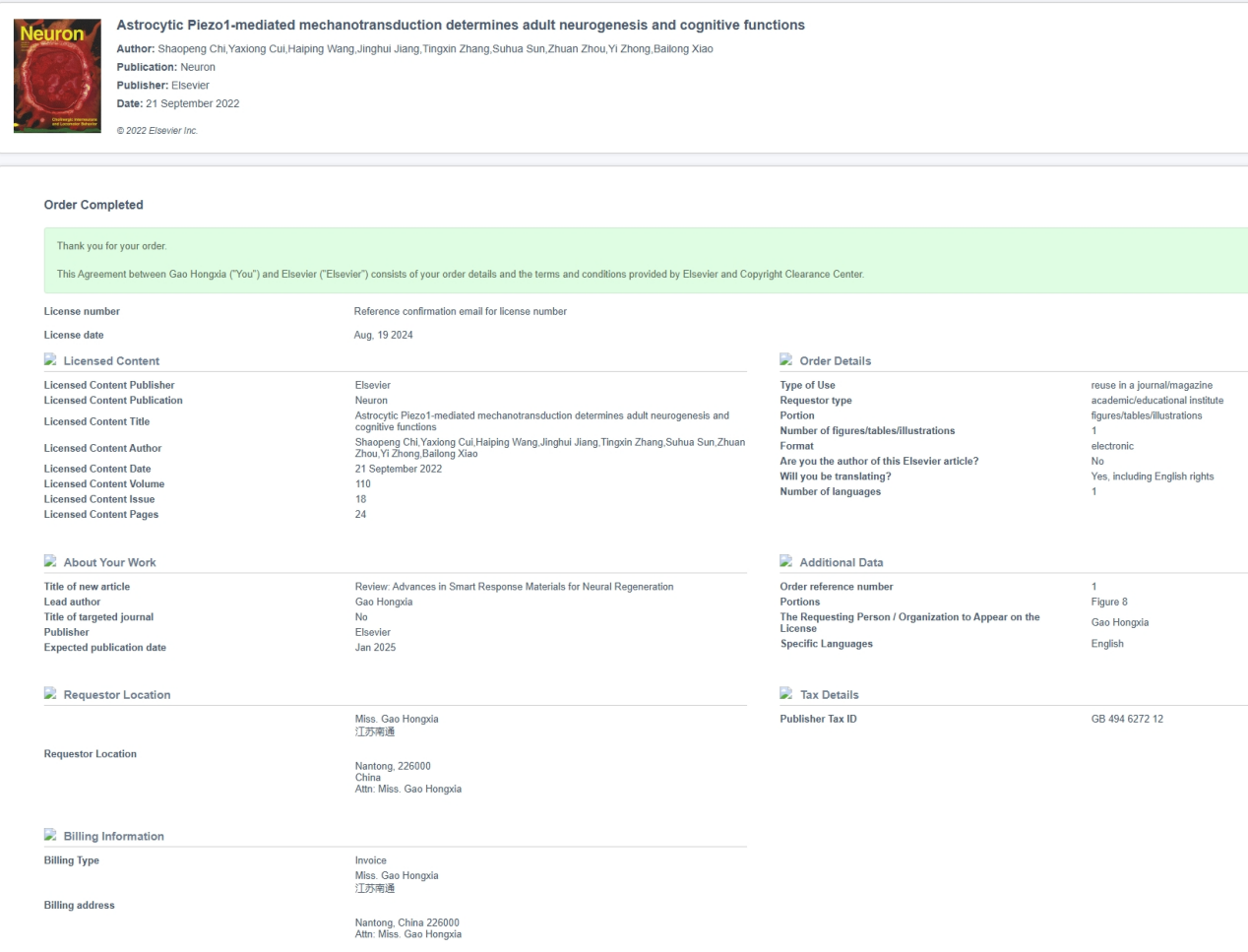


Fig5 B


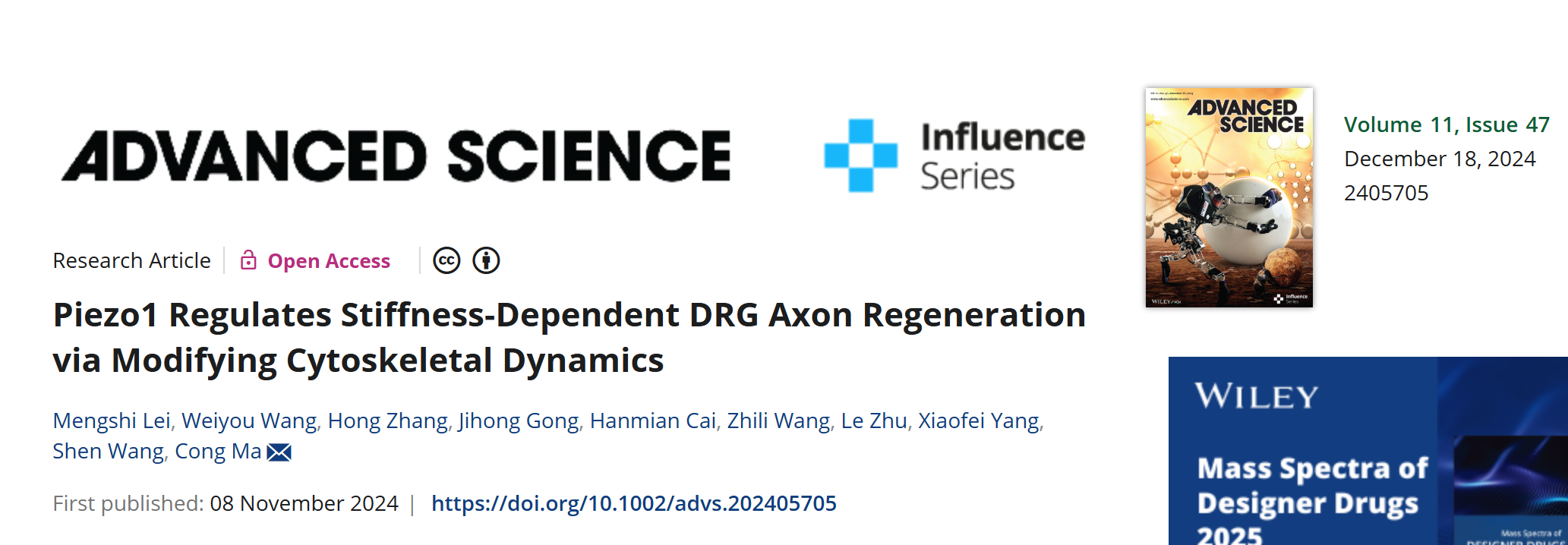


Fig5 C


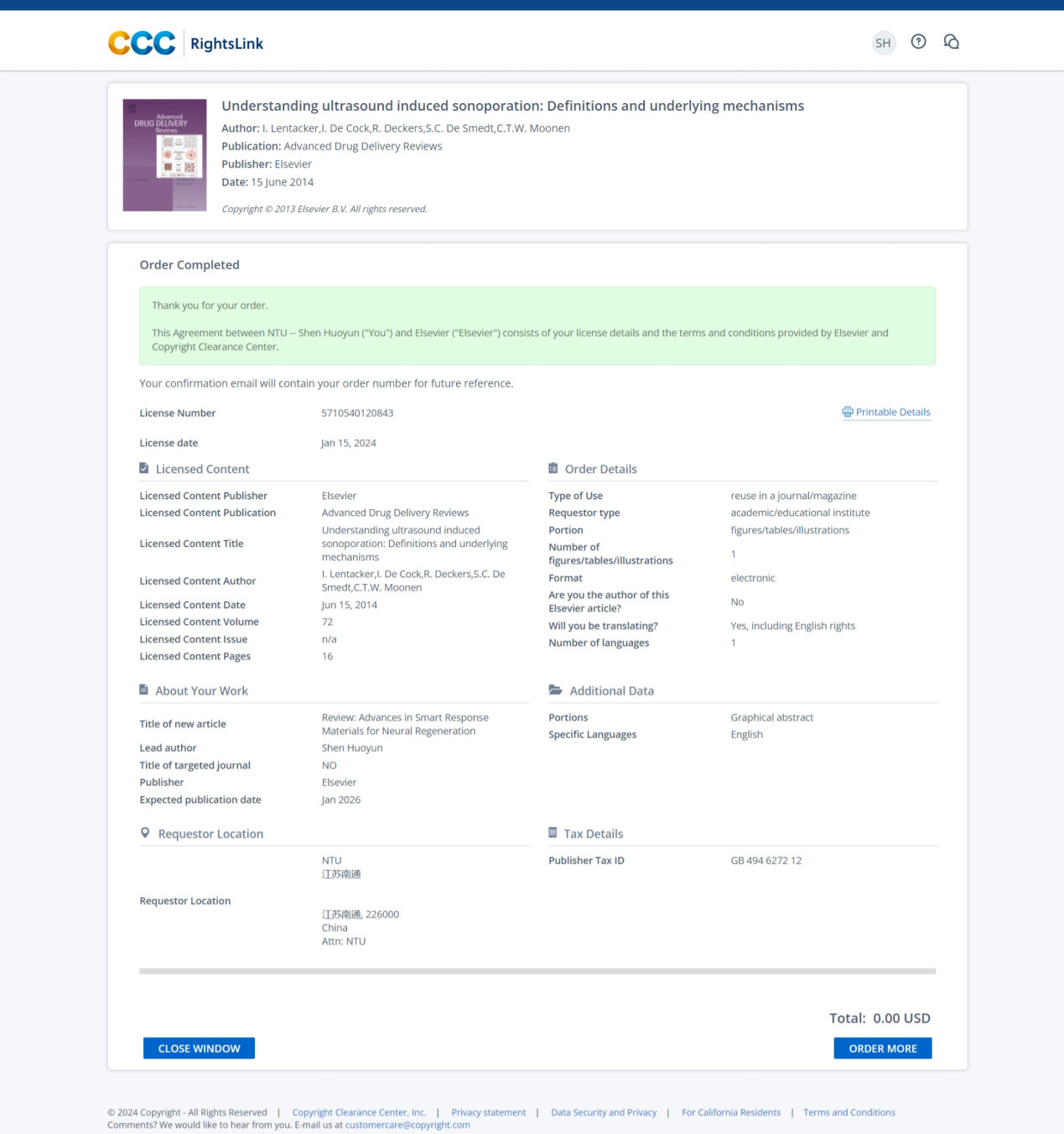


Fig5 D


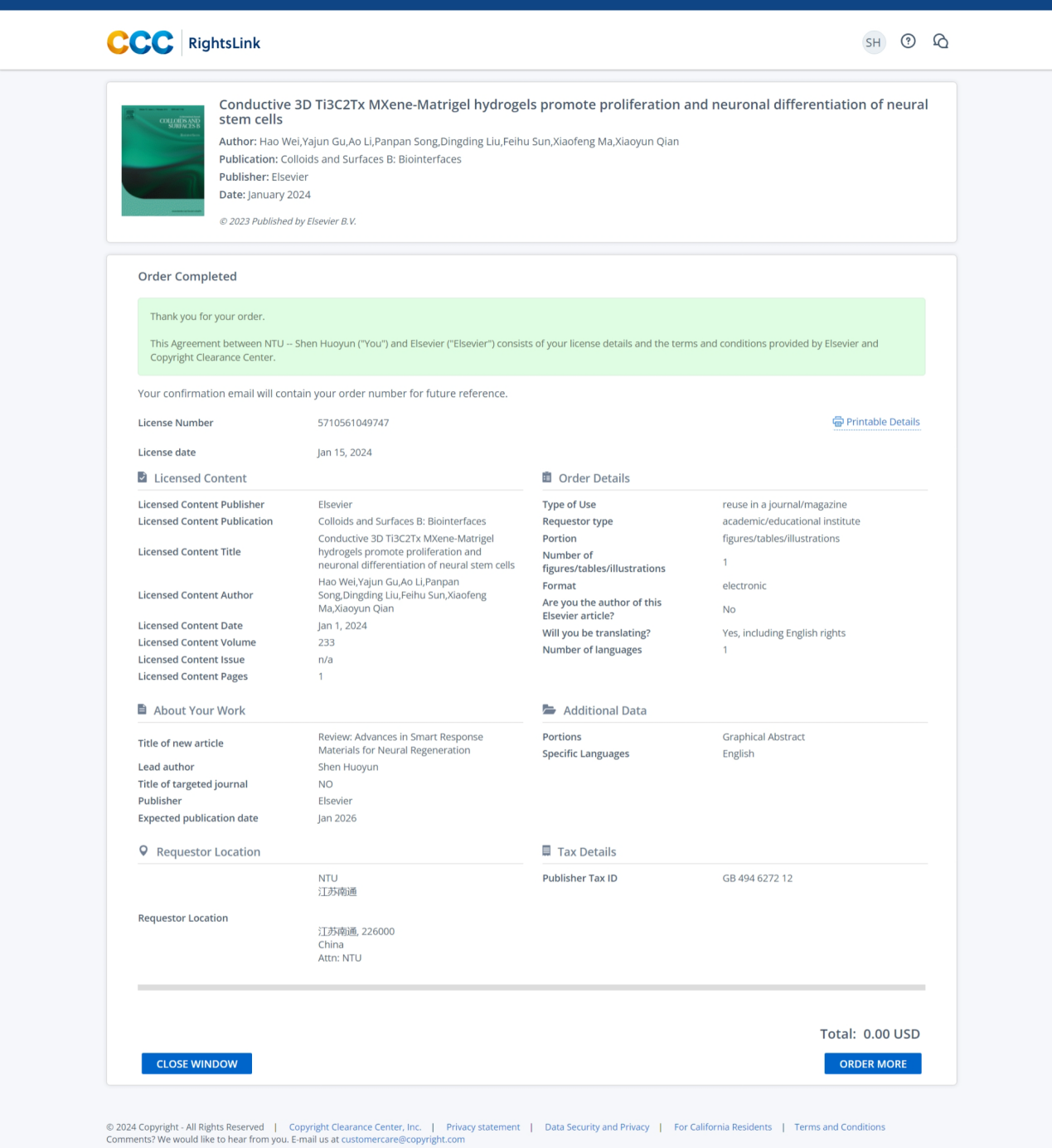


Fig5 E


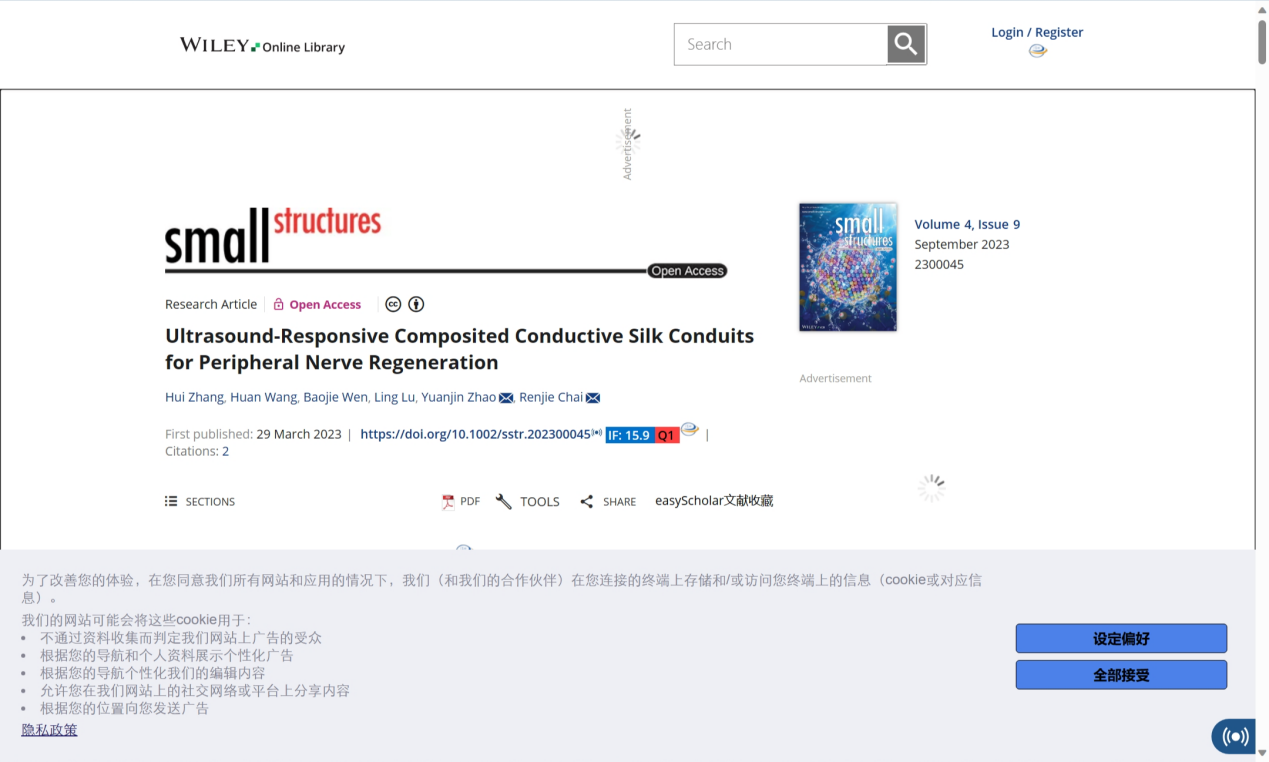


Fig6 A


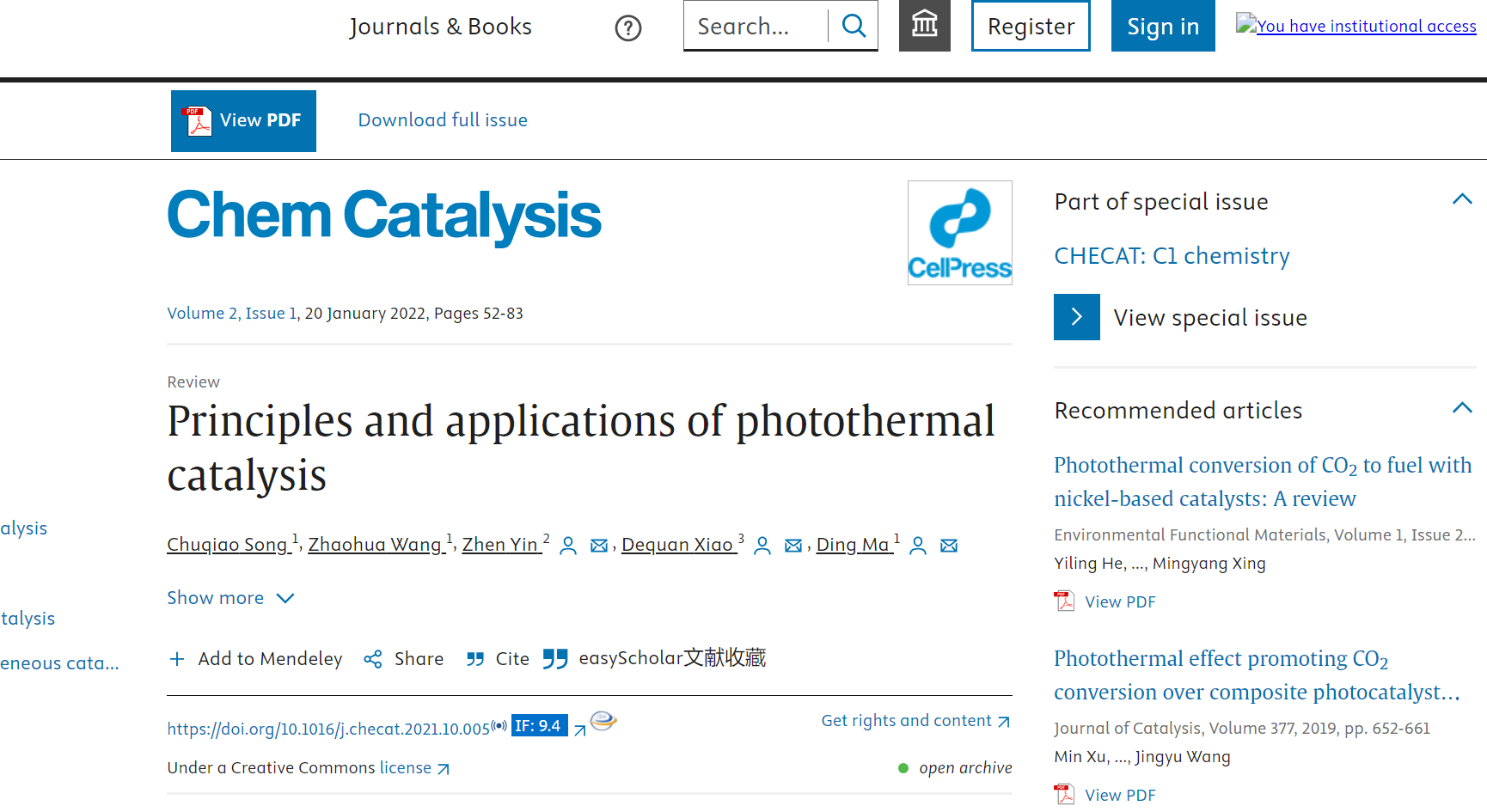


Fig6 B


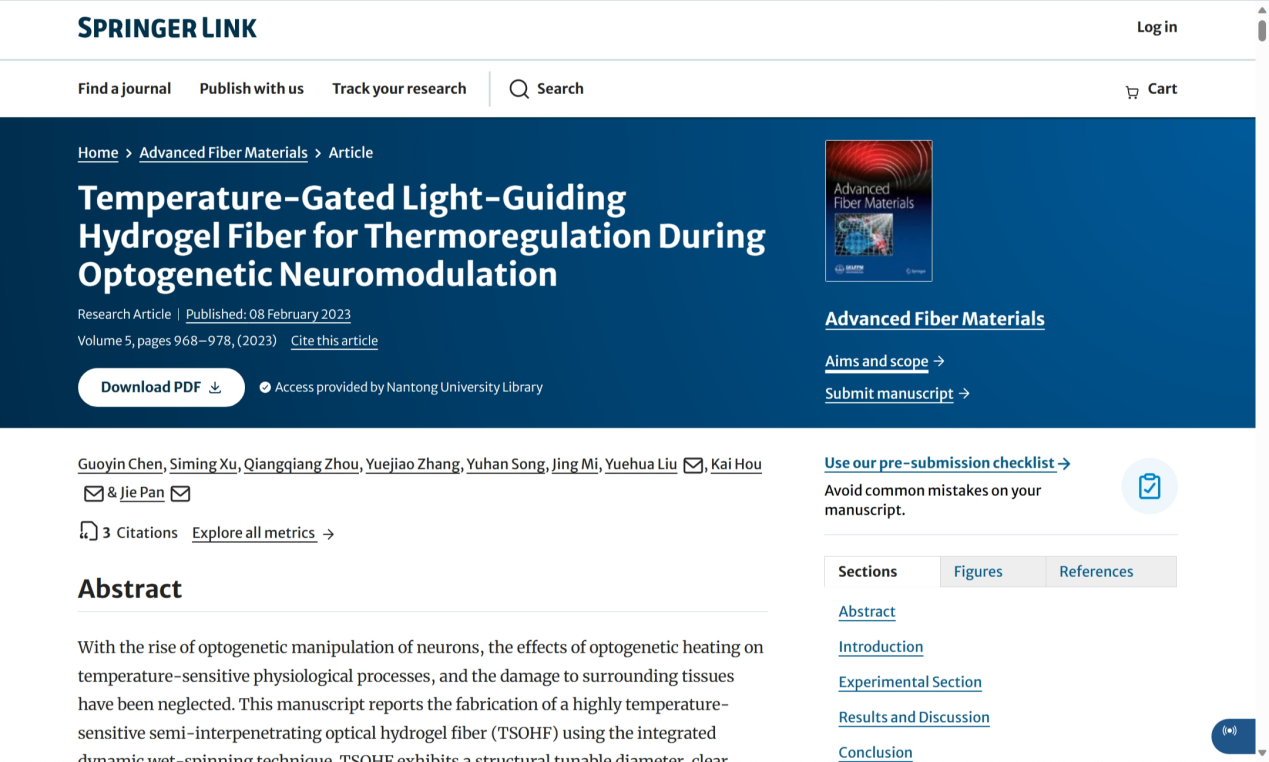


Fig6 C


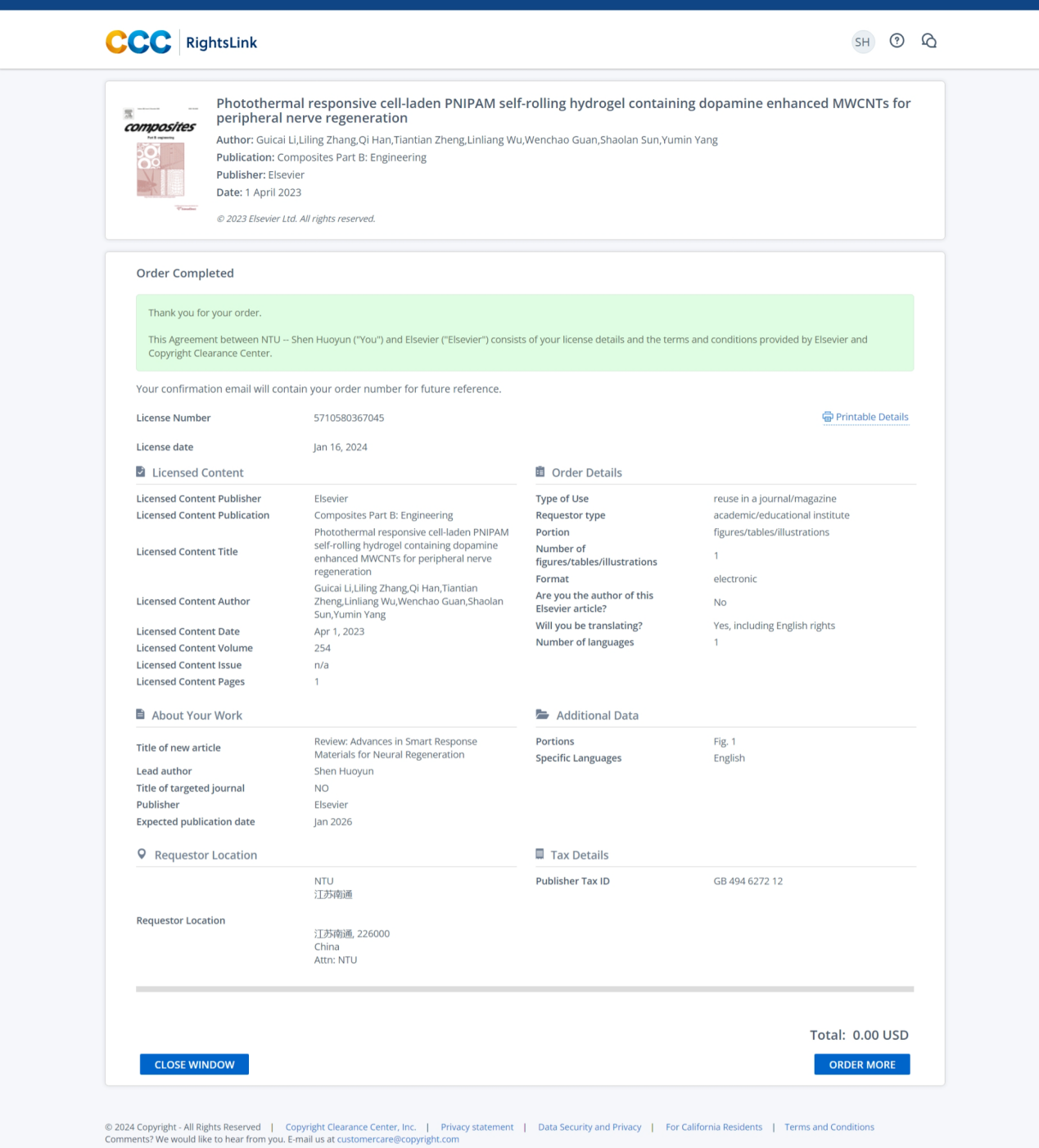


Fig6 D


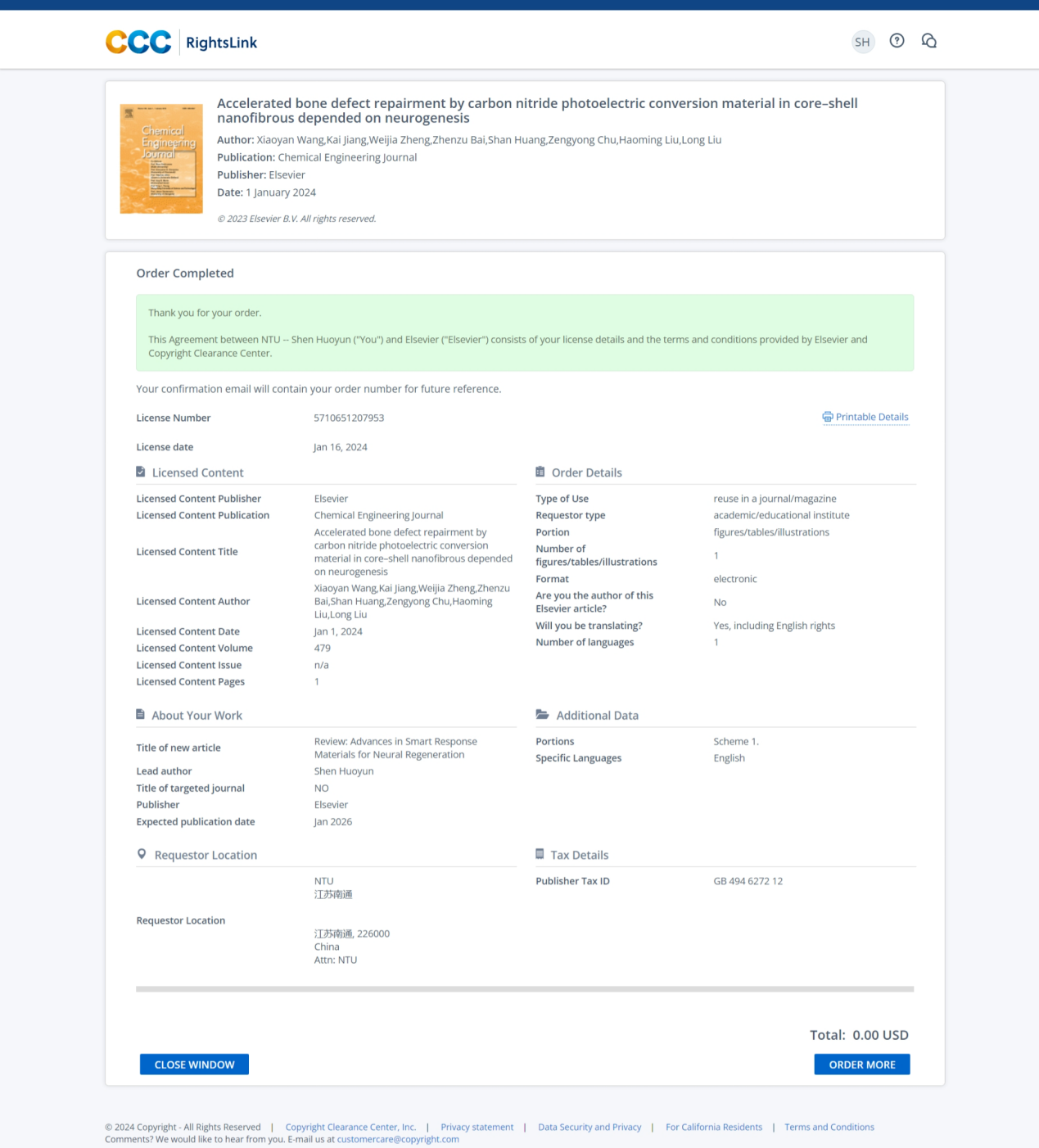


Fig6 E


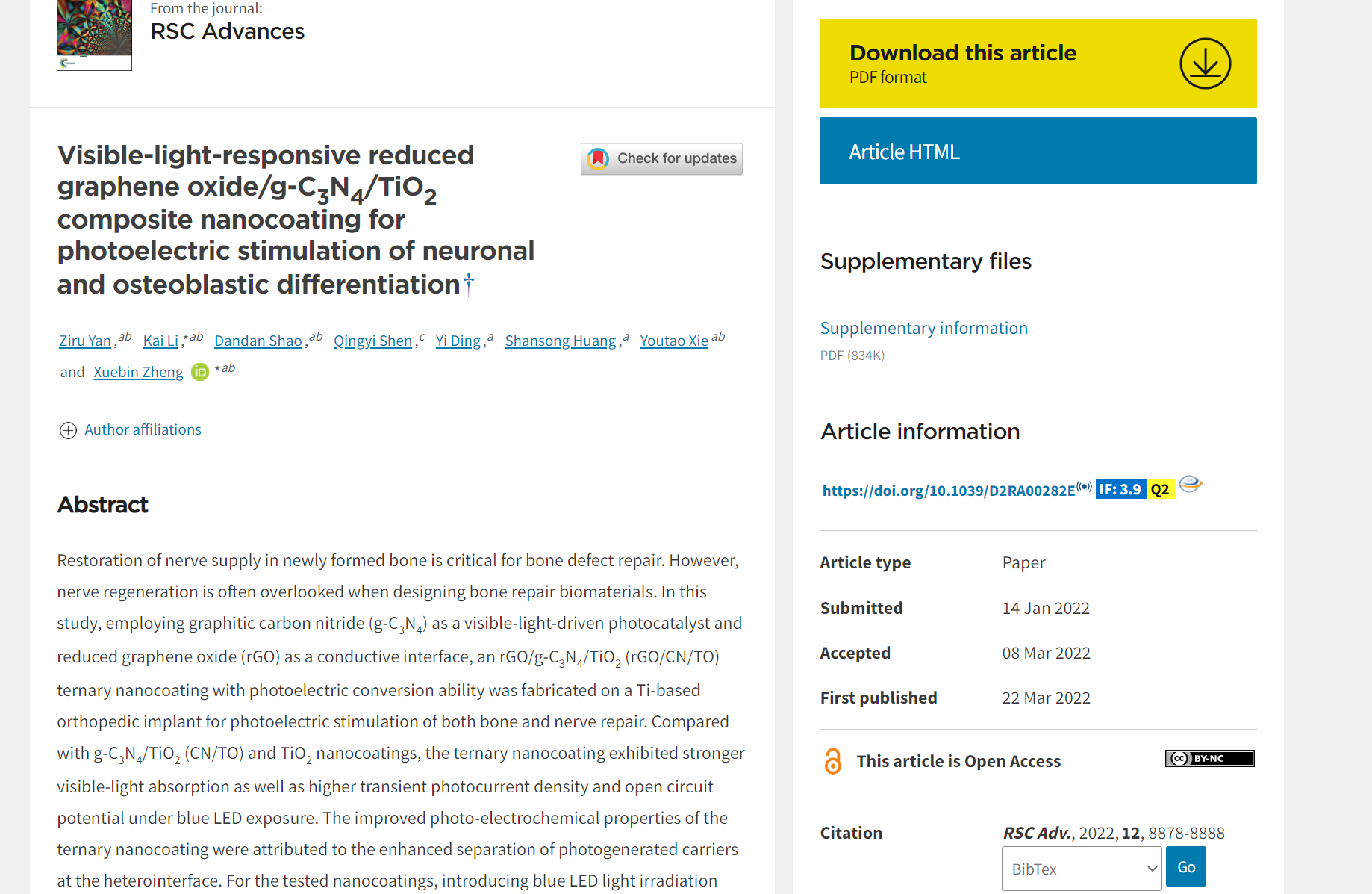


Fig7 A


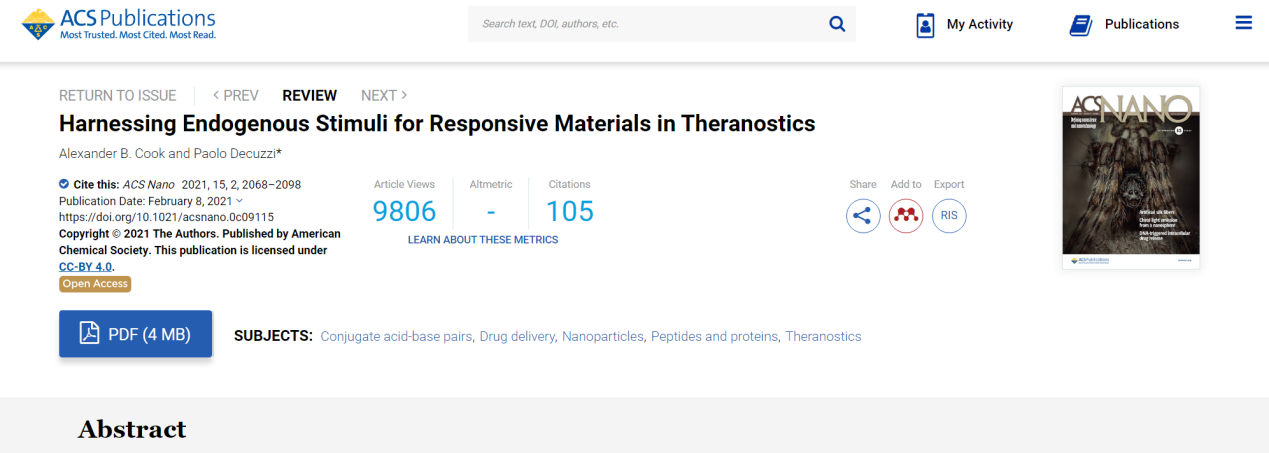


Fig7 B


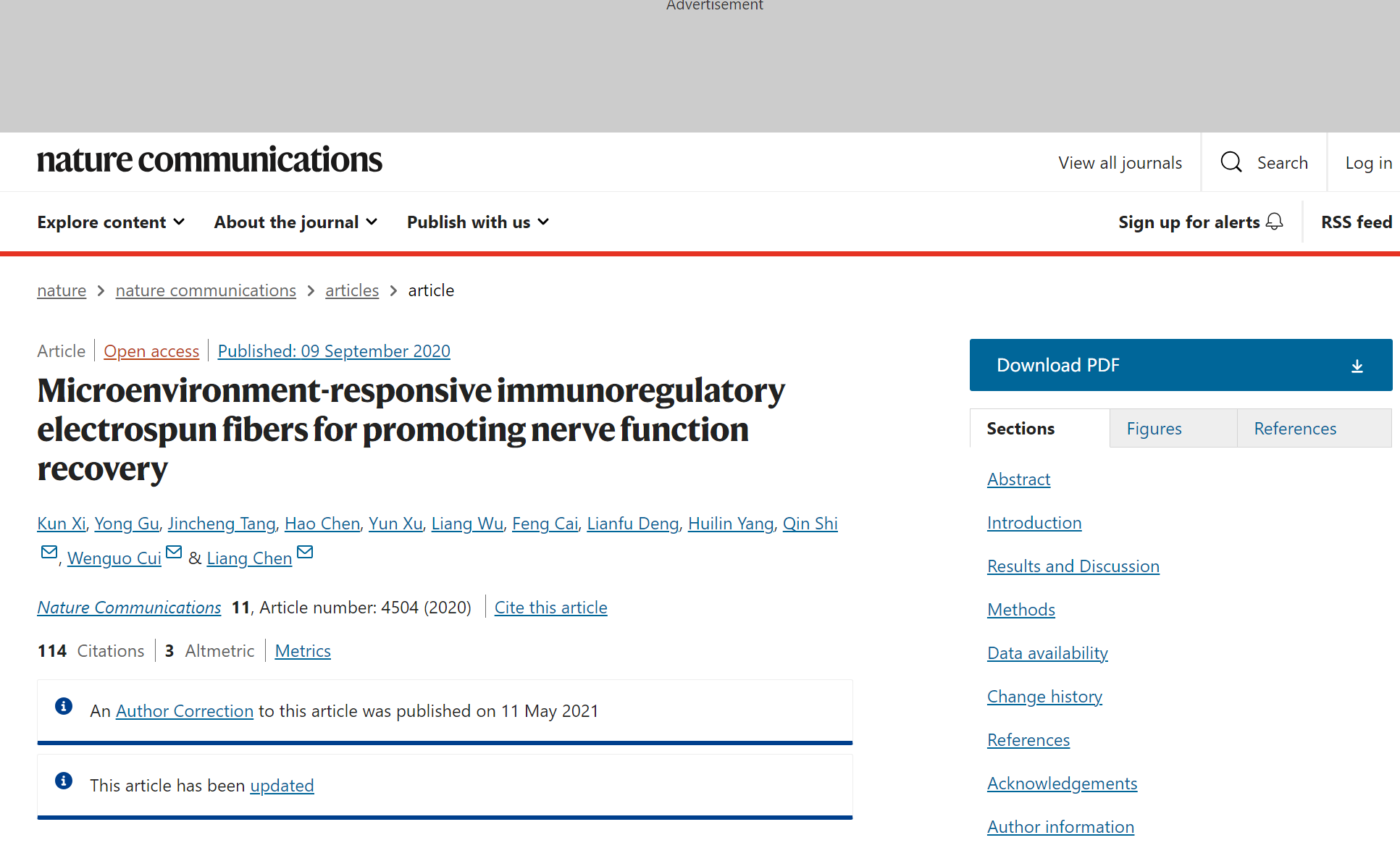


Fig7 C


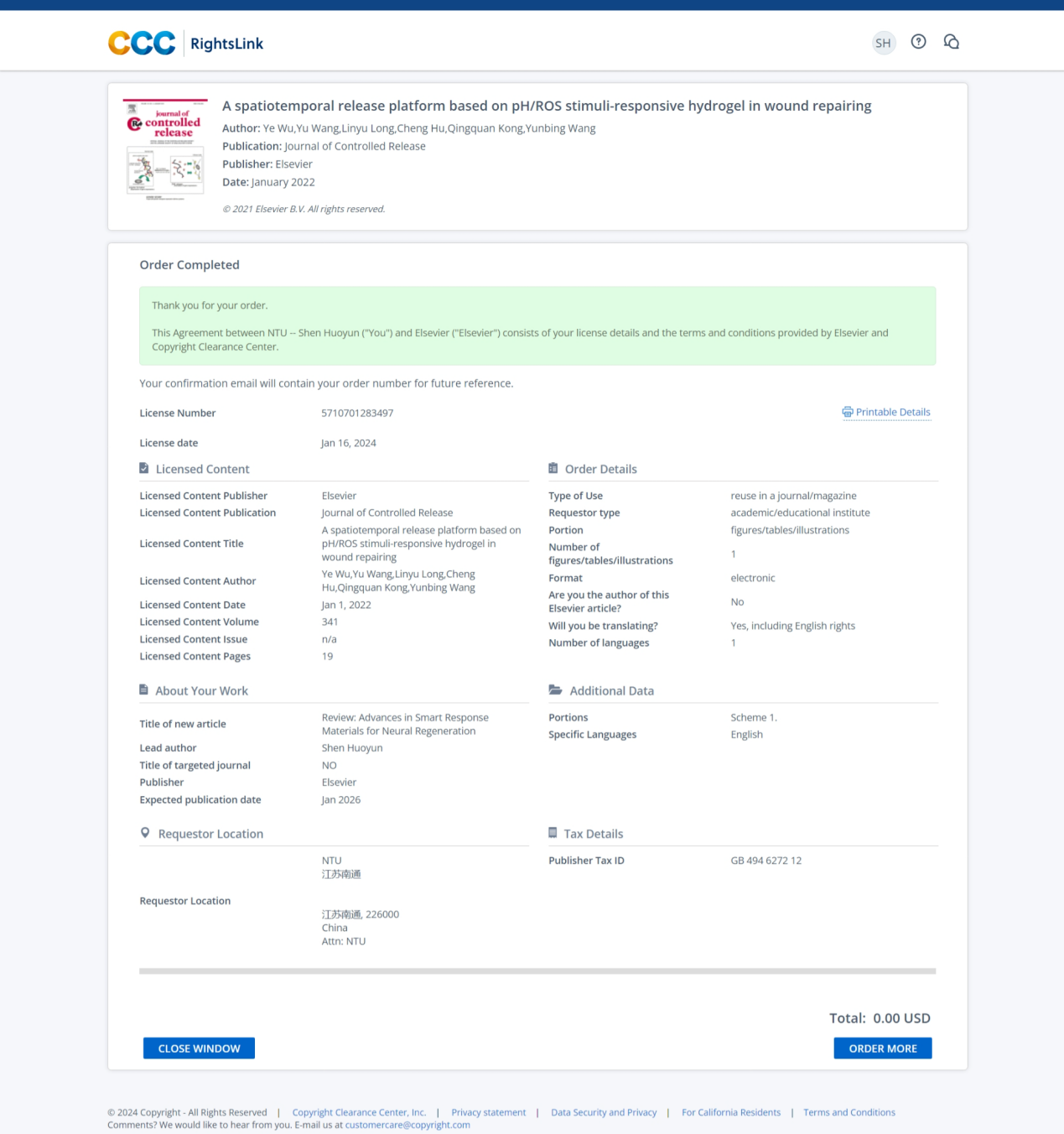


Fig7 D


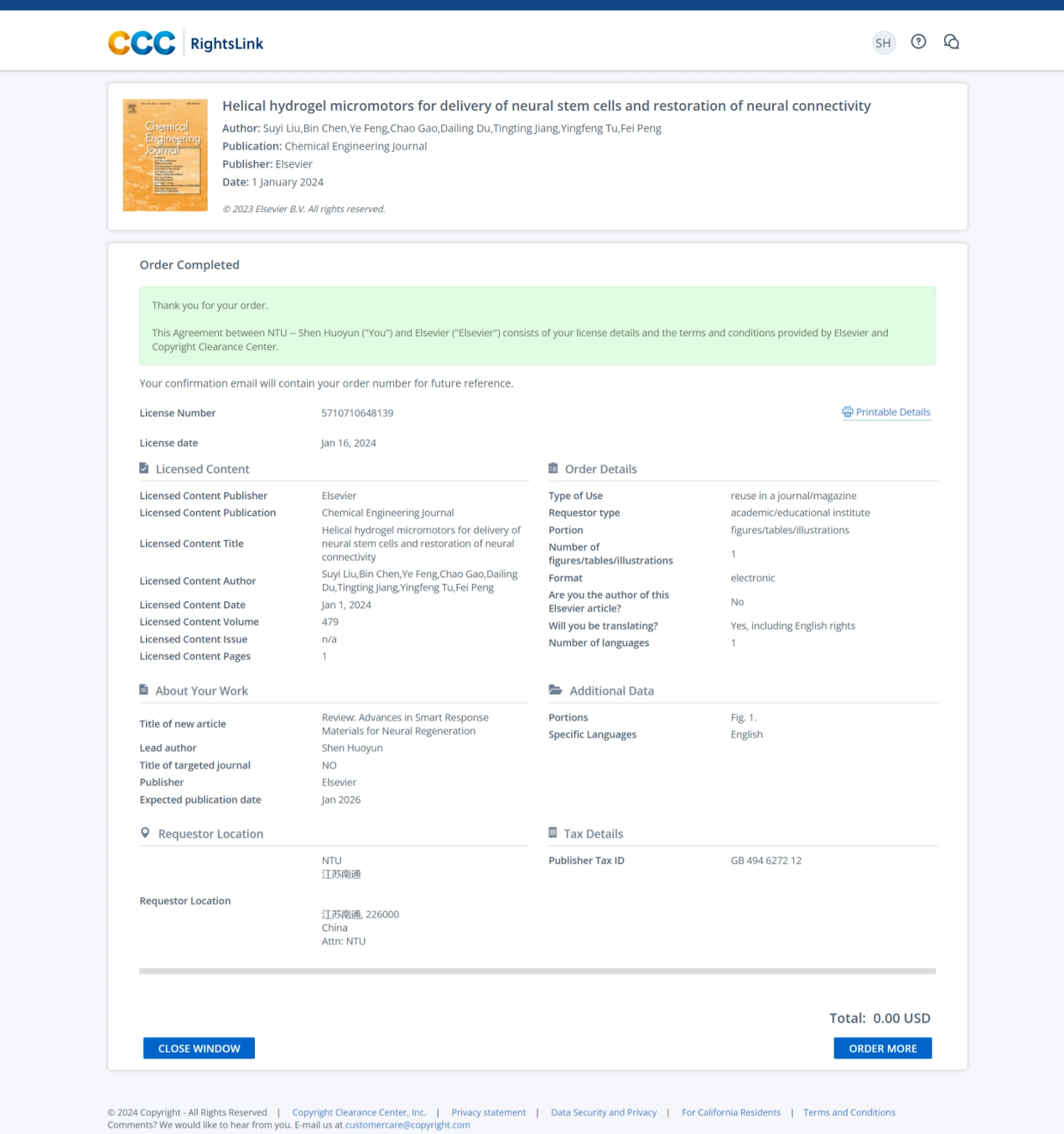


Fig7 E


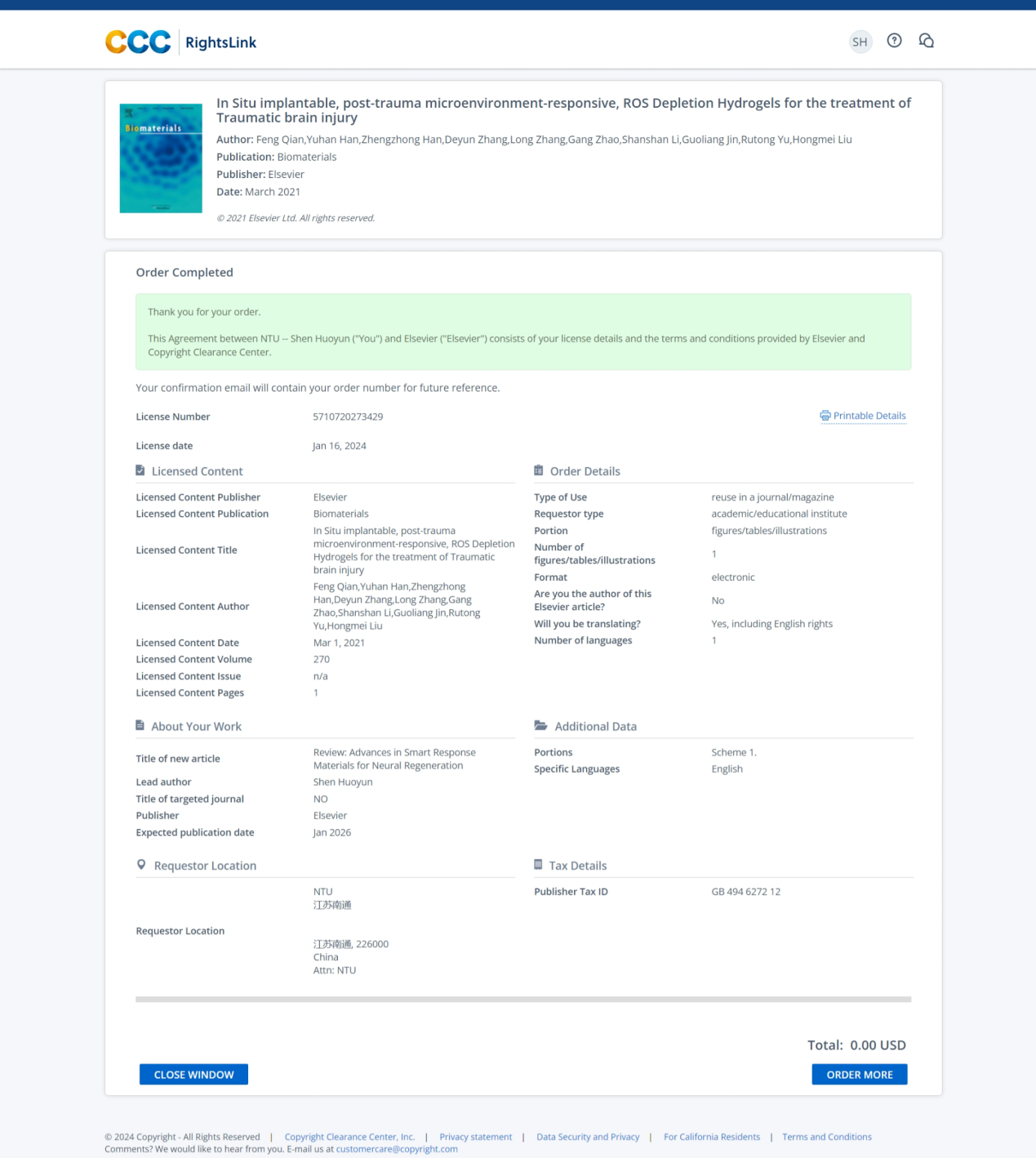


Fig8 A


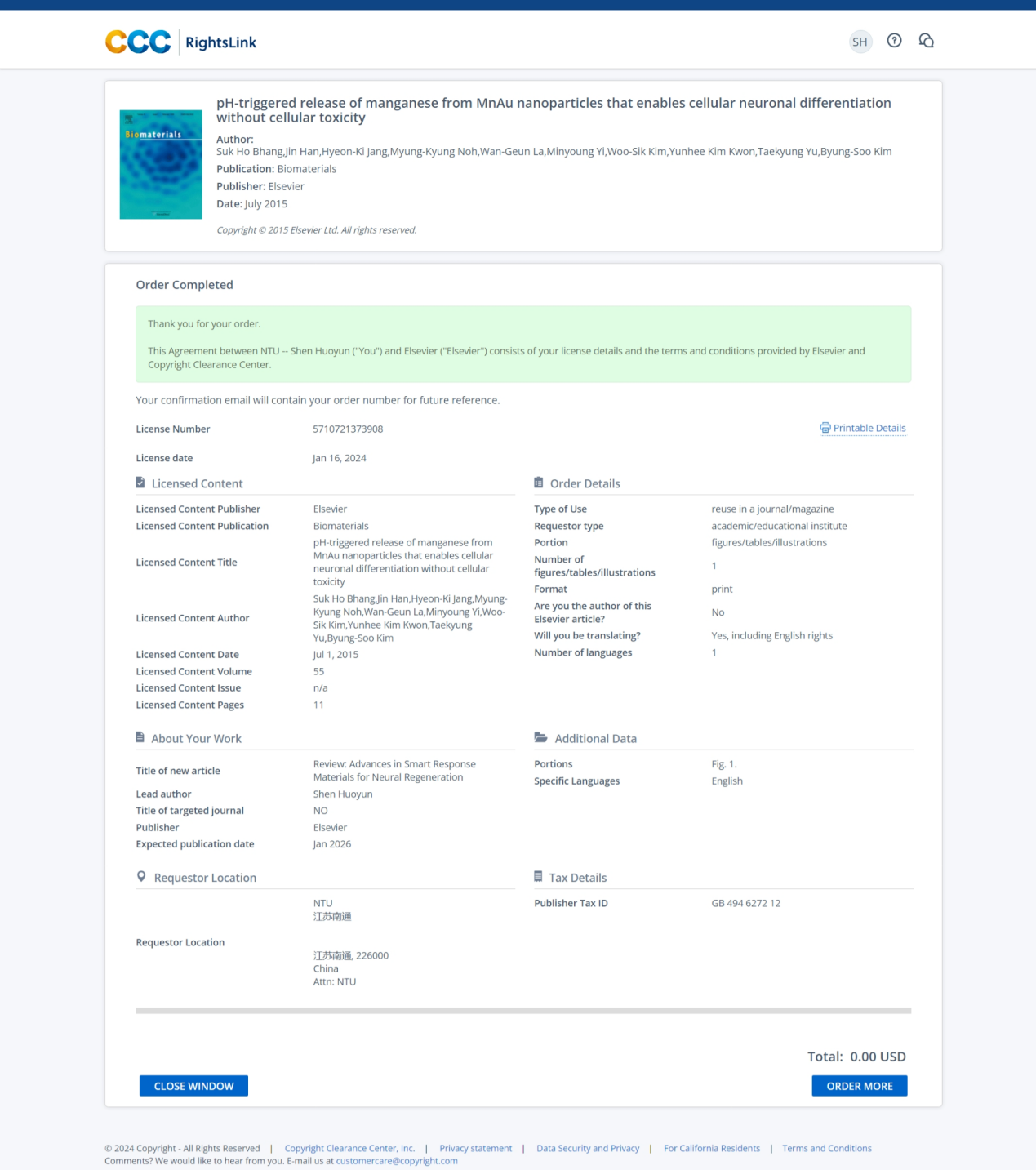


Fig8 B


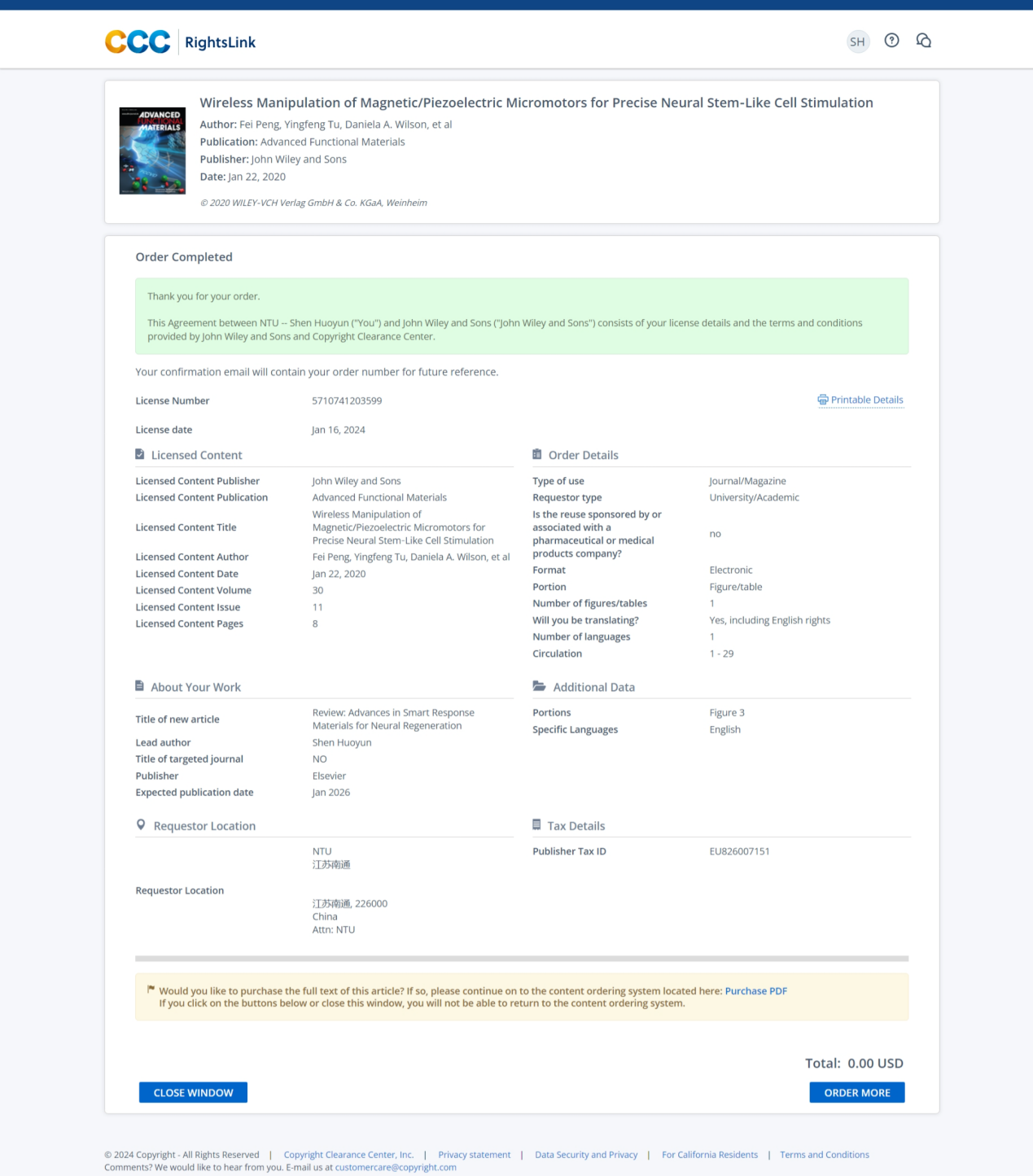


Fig8 C


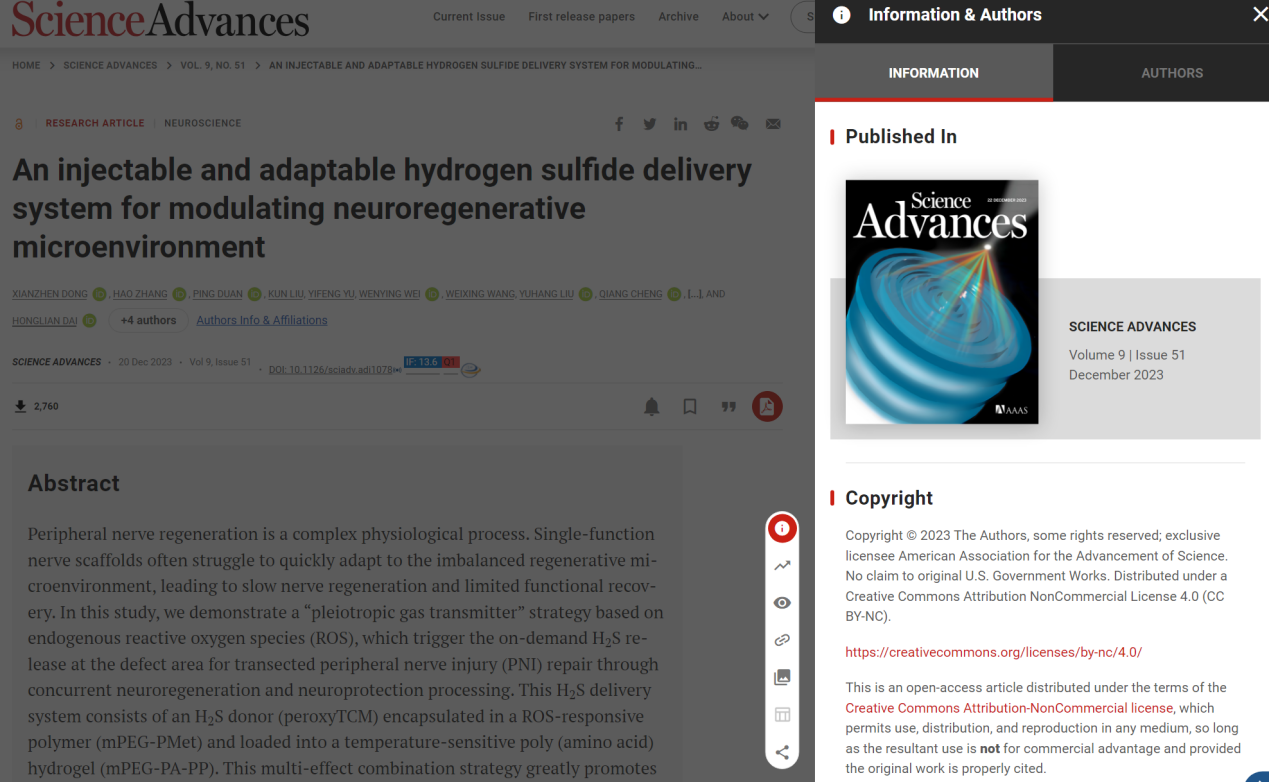

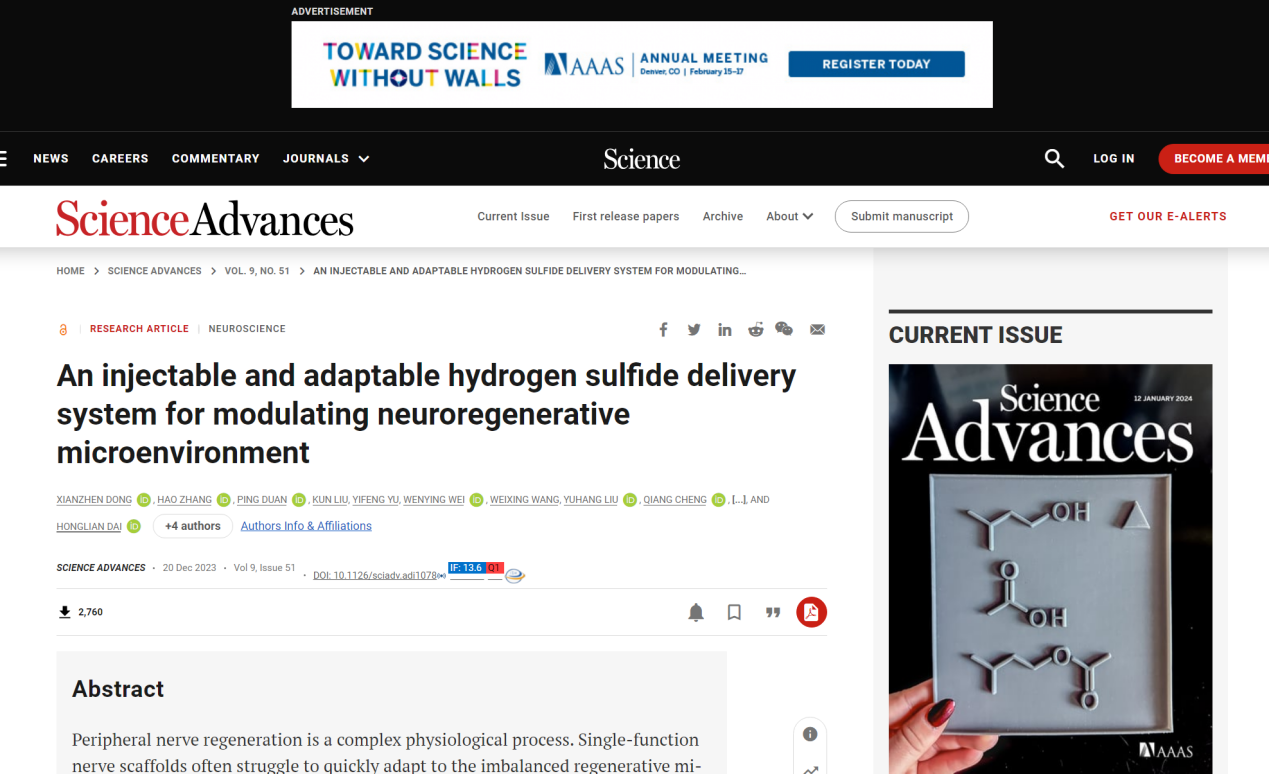

Supplement: Multimedia component 1 [file mmc1.docx]
